# Efficient Replication of over 180 Genetic Associations with Self-Reported Medical Data

**DOI:** 10.1371/journal.pone.0023473

**Published:** 2011-08-17

**Authors:** Joyce Y. Tung, Chuong B. Do, David A. Hinds, Amy K. Kiefer, J. Michael Macpherson, Arnab B. Chowdry, Uta Francke, Brian T. Naughton, Joanna L. Mountain, Anne Wojcicki, Nicholas Eriksson

**Affiliations:** 1 23andMe, Inc., Mountain View, California, United States of America; 2 Department of Genetics, Stanford University, Stanford, California, United States of America; Leiden University Medical Center, The Netherlands

## Abstract

While the cost and speed of generating genomic data have come down dramatically in recent years, the slow pace of collecting medical data for large cohorts continues to hamper genetic research. Here we evaluate a novel online framework for obtaining large amounts of medical information from a recontactable cohort by assessing our ability to replicate genetic associations using these data. Using web-based questionnaires, we gathered self-reported data on 50 medical phenotypes from a generally unselected cohort of over 20,000 genotyped individuals. Of a list of genetic associations curated by NHGRI, we successfully replicated about 75% of the associations that we expected to (based on the number of cases in our cohort and reported odds ratios, and excluding a set of associations with contradictory published evidence). Altogether we replicated over 180 previously reported associations, including many for type 2 diabetes, prostate cancer, cholesterol levels, and multiple sclerosis. We found significant variation across categories of conditions in the percentage of expected associations that we were able to replicate, which may reflect systematic inflation of the effects in some initial reports, or differences across diseases in the likelihood of misdiagnosis or misreport. We also demonstrated that we could improve replication success by taking advantage of our recontactable cohort, offering more in-depth questions to refine self-reported diagnoses. Our data suggest that online collection of self-reported data from a recontactable cohort may be a viable method for both broad and deep phenotyping in large populations.

## Introduction

In the last few years, the cost of collecting genomic data has declined rapidly. However, advances in the collection of phenome data (the set of all phenotypic information from a single organism) have not kept pace [1], [2]. New techniques are needed to complement the wealth of genomic data and build the large cohorts needed for highly-powered genome-wide association studies (GWAS).

The reliability of phenotyping is important for GWAS. Phenotyping error decreases power, which can be problematic as most GWAS are not sufficiently powered to explain a significant fraction of the underlying heritability. Although increasing sample sizes can counteract the problems caused by misclassification, it is the very issue of needing ever larger samples that necessitates more efficient methods for collecting data [3]. A number of associations reported in very large meta-analyses have not been replicated, and may never be, simply because of the difficulty of assembling such a sizeable cohort of patients. There is a need for more straightforward methods to quickly and reliably gather retrospective phenotype information from large cohorts of people, not only to validate existing associations, but to discover new ones.

Although self-report has frequently been used for physical traits, medical records have traditionally been the preferred source of retrospective information on medical conditions. Previous studies have reported good agreement between medical record data and self-reported medical conditions [4]–[8], and include a few common themes. First, there tends to be good concordance for well-defined and easily diagnosed diseases and for chronic conditions that require repeated medical follow-up (kappa ranging from 0.71–0.80 for diabetes, hypertension, myocardial infarction, and stroke) [4]–[9]. Second, a negative self-report is very likely to agree with a negative result from the medical record [5]. Third, self-reports of conditions that are milder, less specific (such as heart failure), or communicated in different ways by physicians (such as high cholesterol) tend to be less consistent with medical records, possibly because the original diagnosis was less certain or because of insufficient physician—patient communication [4], [8], [10]. Fourth, medical records, especially in countries without centralized healthcare, typically only address diseases from a limited portion of a patient's life; self-report can be more accurate for diseases outside this window [5].

To begin to address the phenomics problem, a structure that facilitates both broad and deep phenotyping and maximizes the utility of information gathered while minimizing the burden on participants is needed. In this study, we evaluate a research model in which a large, recontactable cohort is surveyed online across a broad range of phenotypes. Subsets of this cohort with particular characteristics can then be contacted for further research with more in-depth phenotyping on specific topics as appropriate. We have demonstrated previously that this model can be used to discover and replicate associations with non-medical traits [11]. Here, by assessing our ability to replicate previously reported genetic associations across a wide range of conditions, we demonstrate that broad self-reported data collection online is useful for medically-related conditions as well. We show that some classes of conditions lend themselves particularly well to simple self-report, while others are more complex. We also show that the ability to recontact the cohort facilitates rapid refinement of phenotype characterization.

## Results

We sought to replicate associations from the list curated by the National Human Genome Research Institute's Office of Population Genomics (“GWAS catalog”) in a cohort of 20,182 participants of European ancestry who filled out surveys on the 23andMe website [12], [13]. Members of the cohort, drawn from the 23andMe customer base, had been genotyped at approximately 600,000 SNPs, and had access to their raw genetic data as well as health- and ancestry-related interpretations of their data. The majority of the cohort was not selected based on disease status or other characteristics and is roughly a representative sample from the 23andMe customer base; approximately 4,000 members of the cohort had been recruited for a study on Parkinson's disease or a project with the National Senior Games Association (http://www.nsga.com/).

Phenotypes from the GWAS catalog were matched with available phenotypes from the online surveys (see Methods). In order to collect data on a wide range of phenotypes while keeping the time spent answering surveys low, we chose to assess most phenotypes using only single questions of the general form “Have you ever been diagnosed by a doctor with [Condition X]?”. A total of 50 conditions from the GWAS list had direct analogues within the 23andMe database. For each condition, we used only one SNP from each linkage disequilibrium (LD) block (using a threshold of r^2^> = 0.1) and removed SNPs that were not on our platform or did not have a proxy SNP on our platform with LD of r^2^> = 0.5. Ultimately, we attempted to replicate a total of 392 different associations (315 case—control, 77 quantitative) for these 50 phenotypes.

**Figure 1:**
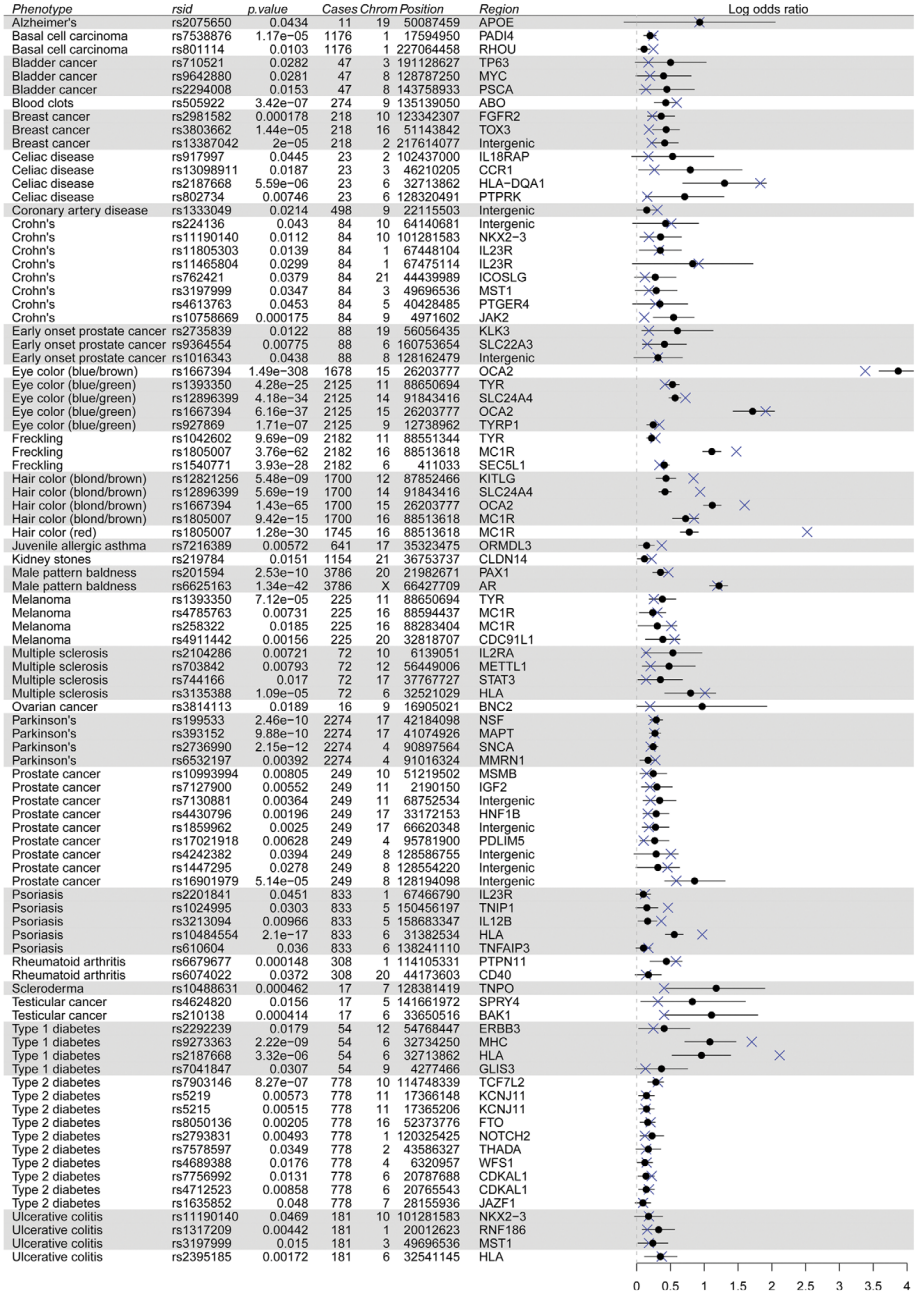
Replicated SNPs for binary traits. Our log ORs and 95% confidence intervals are shown as black circles and lines. Published ORs are shown as blue Xs.

Using a one-sided P<0.05 threshold for significance, we replicated 144 (93 case—control and 51 quantitative) of the 392 attempted associations in 36 of the 50 phenotypes (Figure 1, Table S1, Table S2). For some conditions, the size of our case group was quite small; however, the odds ratios for replicated SNPs were generally in good agreement with the published odds ratios. Of the case—control conditions, 84.6% of the replications had 95% confidence intervals containing the published odds ratio (replications for pigmentary phenotypes such as eye color, hair color, and freckling were not included as the assessment scales are not easy to match across different published reports). As one aspect of 23andMe's Personal Genome Service involves returning genetic data to our customers, we investigated the possibility that customers viewing a result of elevated risk for a certain disease before answering surveys may skew the results towards replication. To address this, we investigated the impact of seeing genetic risk results before versus after answering survey questions on self-reported disease status for a set of 20 conditions for which participants were able to view a personal risk prediction. We observed that in general, the nature of the genetic risk result did not have a consistent or significant effect on the way questions were answered (Table S4).

As many of the known associations were discovered in large case—control studies, we expected to have low power to detect many of them using our mostly unselected cohort. Thus, to further assess our success in replication, we calculated our power to replicate each association for all case—control phenotypes (quantitative phenotypes were skipped in this calculation due to difficulties in matching scales in many of them). Power was calculated using the odds ratios reported in the GWAS catalog. To allow for phenotyping error, the calculations assumed that 5% of our reported cases are actually controls and that the minimum of the prevalence or 10% of our controls are (or will someday be) cases. For diseases with over 10% prevalence, controls were generally chosen to be of sufficient age so that at most 10% of people without the disease at that age would be expected to develop the disease. See Methods for full details.

**Figure 2:**
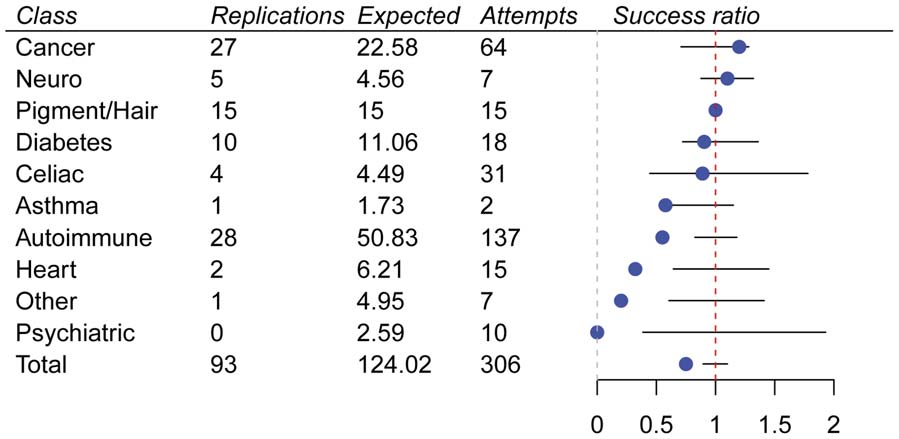
Success rate (versus total power) by disease class. Replications = number of associations we successfully replicated. Expected = number of associations we expected to replicate. Attempts = number of associations we attempted to replicate. The blue dot represents our success ratio (number of successful replications divided by number of expected replications). The black line represents the 95% prediction interval for the success ratio. The nine associations that we had high power to detect but had known conflicting data were not included in this figure (see text and Table 1). Conditions assigned to each class (also see Methods S1): Asthma: childhood asthma; Autoimmune: Crohn's disease, inflammatory bowel disease, lupus, multiple sclerosis, psoriasis, type 1 diabetes, ulcerative colitis; Cancer: basal cell carcinoma, bladder cancer, breast cancer, colorectal cancer, prostate cancer, lung cancer, melanoma, pancreatic cancer, scleroderma, testicular cancer, thyroid cancer; Celiac: celiac disease; Diabetes: type 2 diabetes; Heart: blood clots, coronary artery disease, heart attack; Pigment/Hair: eye color, freckling, hair color, red hair color, male pattern baldness; Neuro: Alzheimer's disease, autism, Parkinson's disease; Other: chronic obstructive pulmonary disease, kidney stones, stroke, osteoarthritis; Psychiatric: alcohol abuse, bipolar disorder, schizophrenia.

We replicated 70% of the number of replications expected (93 replications against 132.7 expected), given our sample sizes (Figure 2). Some of the failed replications can be traced back to the possibility that the reported effect sizes for these associations are inflated or that the associations themselves are false positives. Table 1 shows all associations for which we had at least 80% power to replicate but failed to do so. Of these 19 associations, five have failed to replicate elsewhere despite high power to do so, two have shown significant heterogeneity of odds ratios in meta-analyses, and two exhibited significant signals when the two stages of a multi-stage study were combined but were not interpreted as significant by the authors. Removal of these nine SNPs for which our power may be substantially overestimated increased our replication rate to about 75% (93 out of 124.0). Furthermore, among the remaining 213 SNPs that we did not replicate, we observed the correct directionality of association for 126 out of an expected 172.0 SNPs (using a p-value threshold set to 0.5), yielding a rate of 73.2% relative to expected. Interestingly, an inflammatory bowel disease (IBD) association (rs7517847) that we had high power to replicate also failed to replicate in ulcerative colitis only cases in an Italian study of IBD, suggesting that the association may be specific to Crohn's disease, as opposed to all types of IBD [14]. Overall, our success rates differed vastly for different classes of diseases (Figure 2, Figure S1), suggesting that the difference between theoretical and actual power is to some extent explained by differences in phenotyping (discussed in more detail below).

**Table 1:** Associations with sufficient power for detection (> = 80%) that failed to replicate.

Phenotype	SNP	Pub OR	Rep OR	P-value	Power	Cases	Controls	Replications in the Literature
Alcohol abuse	rs7590720	1.35	0.955	0.875	1	1811	8549	Failed to replicate [30]
Bipolar disorder	rs1012053	1.59	1	0.485	1	366	13030	Failed to replicate [31]–[34]
Bipolar disorder	rs420259	2.08	0.966	0.659	1	366	13030	Failed to replicate [32]–[34]
COPD^a^	rs13180	1.3	1.05	0.26	0.89	403	2306	Replicated [35]
COPD^a^	rs7671167	1.32	1.11	0.0968	0.93	403	2306	Replicated [35]
COPD^a^	rs1828591	1.38	1	0.489	0.97	403	2306	Replicated [36], [37]
Crohn's disease	rs2066847	3.99	1.54	0.151	0.88	84	13288	Replicated [38]
IBD^b^	rs7517847	1.61	0.855	0.954	1	250	12808	Replicated [39] c
Juvenile allergic asthma	rs2786098	1.43	1.07	0.181	1	641	6584	Failed to replicate [40]
Lupus	rs3131379	2.36	1.38	0.133	0.82	52	11675	Not yet replicated
Parkinson's disease	rs17115100	1.25	0.992	0.555	0.97	2274	5336	Not claimed [41] d
Parkinson's disease	rs823128	1.52	1.17	0.0531	1	2274	5336	Not claimed [41] d
Psoriasis	rs20541	1.27	1.09	0.0953	0.92	833	4291	Replicated [42], [43]
Rheumatoid arthritis	rs10499194	1.33	0.927	0.797	0.9	308	12845	Failed to replicate [44]
Rheumatoid arthritis	rs3761847	1.32	1.01	0.437	0.93	308	12845	Between-study heterogeneity [45]
Type 2 diabetes	rs9300039	1.48	0.976	0.595	0.97	778	3273	Between-study heterogeneity [46]
Type 2 diabetes	rs2943641	1.19	1.03	0.328	0.81	778	3273	Not yet replicated
Thyroid cancer	rs965513	1.75	1.37	0.0559	0.83	52	11234	Replicated [47]
Ulcerative colitis	rs11209026	1.79	1.47	0.0577	0.85	181	13100	Replicated [48]

Pub OR = published odds ratio. Rep OR = 23andMe attempted replication odds ratio. Power = estimated power to detect association.

a COPD = Chronic Obstructive Pulmonary Disease. This analysis included smokers only.

b IBD = Inflammatory Bowel Disease.

c This SNP was initially associated with IBD, but replicated only for Crohn's disease [39], which is a subtype of IBD. Latiano et al. also replicates rs7517847 with Crohn's disease, but not with ulcerative colitis, which is the other major subtype of IBD [14].

d This association was curated into the GWAS catalog as significantly associated with Parkinson's disease but was not identified by the authors as significant.

Separate from these calculations, we also attempted to replicate 106 associations with phenotypes in our cohort that were only in weak correspondence with phenotypes in the published papers. Of the 106, we replicated 39 associations. For example, while we did not collect data on gallstones, we did collect data on gall bladder surgery which is often a consequence of gallstones, and were able to replicate one association with gallstones. Likewise, answers to the question “Have you ever been diagnosed by a doctor with high cholesterol (over 200 mg/dl) or hypercholesterolemia?” were sufficient to replicate 19 associations with cholesterol level. Data on receiving an abnormal result on a liver function test result were sufficient to replicate four associations with bilirubin levels. A summary list of these replications can be found in Table 2 (full list in Table S3).

**Table 2:** Replications without strictly matching phenotypes.

23andMe Phenotype	Published Phenotype	# Replications	Genes
Liver test	Bilirubin levels	4	CHUK, GGT1, SAMM50, UGT1A1
High cholesterol	Cholesterol levels (quantitative)^a^	19	ABCG8, APOA1, APOB, CELSR2, CILP2, DNAH11, DOCK7, FADS1, GCKR, HNF1A, LDLR, LIPC (×2), MAFB, NCAN, PCSK9 (×2), TOMM40, TRIB1
Gall bladder removal	Gallstones	1	ABCG8
High blood pressure	Blood pressure (quantitative)^a^	8	ATP2B1, CYP17A1 (×2), CYP1A1, FGF5, SH2B3, ULK4, ZNF652
Osteoporosis	Bone mineral density (quantitative)^a^	5	MEF2C, MEPE, OSX, SOX6, SPTBN1
Macular degeneration	Advanced age-related macular degeneration	2	C2, C3
Nicotine abuse	Nicotine dependence	1	CHRNA3

a These phenotypes were measured quantitatively in the published reports, but the corresponding 23andMe phenotypes listed here were measured qualitatively (yes/no).

## Discussion

Advances in technology have driven down the price and difficulty of genotyping, but until recently, the same has not been true of phenotyping [15]. We propose that web-based collection of self-reported data on medical phenotypes is an efficient and effective method for phenotyping a large cohort of individuals, as evidenced by our ability to replicate a high percentage of associations across a wide range of conditions. Relative to medical record review, internet-based phenotyping is fast (we assessed more than 20,000 people for 50 phenotypes in approximately 12 months using only a small team of people). To our knowledge, this is the largest number of replications across a wide variety of diseases ever reported, demonstrating the value of gathering self-reported data on a large genotyped population.

While many of the associations tested here have been replicated before, there are a few that are, based on our literature review, the first independent replications of these associations in a population of European descent: basal cell carcinoma (*PADI4*, *RHOU*), plasma levels of liver enzymes (*PNPLA3*), and bone mineral density (*MEF2C*, *MEPE*—these have already been replicated in a population of Asian descent). Though our study has been performed in a population of European ancestry, a similar study would be feasible in other populations. Such a study could potentially improve risk prediction in non-European populations as well as further our understanding of disease architecture (e.g., understanding how effect size varies across populations could provide insight into how tightly linked associations are to the causal variants). Furthermore, while it is true that we are able to replicate previously identified associations using our research platform, the reverse is also true—novel discoveries using our method have been independently replicated using other modes of data collection for both traits and medical conditions [16]–[19].

Although most studies use medical records as the gold standard against which self-reported data are compared, there are some inherent challenges to the use of medical records [5]. As very few people have received all their health care from the same provider, the medical records from different stages of their lives are stored at different sites of care. Thus, a childhood diagnosis of asthma might be stored in a record at the pediatrician's office but not be reported in the record at the adult medical practice. In addition, extracting data from medical records often requires either manual curation, which is time-consuming and expensive, or reliance on ICD-9-CM or CPT codes which may have been miscoded. For example, a replication study was carried out using the BioVU DNA databank at Vanderbilt University by applying natural language processing techniques and billing-code queries to electronic medical records [20]. Their algorithms achieved high positive predictive value (as measured by independent record review by two physicians) but required manual review and significant iterative work. Out of 21 SNPs in five phenotypes, they were able to replicate eight associations. In contrast, we were able to examine 50 phenotypes and replicate over 180 associations. For cases in which the information required may be difficult for individuals to report but can be extracted from electronic medical records (such as lab values), these two methods can provide complementary sources of data.

We replicated approximately 75% of the associations we expected to (excluding those for which our power may be substantially overestimated), based on power calculations. There are several possible reasons why we did not replicate all the associations we expected to (see Figure 2 and Figure S1 for instances in which our success ratio did not overlap the 95% prediction interval). One factor is systematic inflation of odds ratios in the initial reports due to the winner's curse—a bias in the effect size estimates from the first publication to report an association, generally occurring when the discovery sample is poorly powered to detect the association [21]. For example, if we were to assume a systematic inflation of 15% in the log-odds ratio, the replication rate would change from 70% to 77% (or 75% to 82% if we again exclude the nine associations that are not clearly true positives). This amount of inflation is entirely within the confidence intervals for most studies: it corresponds to an estimated odds ratio of 1.3 where the true odds ratio was 1.25 or an estimate of 1.5 where the true odds ratio was 1.41. There are more sophisticated methods to perform bias correction for odds ratios but these require an analysis of the original experimental design that is beyond the scope of this paper [22].

While winner's curse probably explains part of the deviation from expected, some classes of diseases were likely not well phenotyped in this study, through some combination of misdiagnosis and misreport. For example, autoimmune diseases are more challenging because they may be of low prevalence, have non-specific symptoms, and a high rate of misdiagnosis. In a study of rheumatoid arthritis diagnoses by non-rheumatologists, 23–82% were judged to be misdiagnoses [23], while another study showed that relative to assessment in a specialist setting, patients in a community setting who received a diagnosis of celiac disease were actually misdiagnosed more than 50% of the time [24]. Some of the underperformance of this approach for autoimmune diseases is therefore likely due to patients reporting a mistaken diagnosis by a non-specialist.

Because we chose to keep the burden of answering surveys low for our participants, many of the conditions in this study were assessed with single questions such as “Have you ever been diagnosed by a doctor with schizophrenia?” This assessment likely led to reporting errors for some diseases. For example, psychiatric diseases or mental disorders such as Alzheimer's disease, for which diagnosis requires a somewhat subjective clinical evaluation of a patient's symptoms or an autopsy, were each assessed via a single question in this study. More questions are needed here to gather information about the clinical features that led to the diagnosis. In addition, in some cases it may make more sense to have a family member, friend, or caregiver provide information for an individual.

On occasion, the nature of people's answers to such single questions necessitated making judgment calls on how to define a phenotype. Because some people may have type 2 diabetes but are only aware of having high blood sugar, we included people who self-reported having hyperglycemia as type 2 diabetes cases. For chronic obstructive pulmonary disease (COPD), we included individuals who reported having emphysema or chronic bronchitis. However, there are likely to be individuals who repeatedly get bronchitis associated with a cold or flu and reported having “chronic bronchitis”, not knowing that the clinical definition of this condition is developing bronchitis lasting at least three months in two consecutive years. This confusion may have reduced our power to replicate associations with COPD. In other cases, we were unable to come up with an acceptable match for a condition. For example, most GWAS of age-related macular degeneration (AMD) have focused on advanced AMD and generally only included cases with large drusen, geographic atrophy, and/or neovascularization. Our question asked only about AMD without assessing severity and thus our study may have included individuals with small or intermediate drusen and/or pigmentary abnormalities as cases. Such phenotypes from the GWAS catalog without direct analogs in our database were skipped for the main calculations in this paper. For all such conditions, more in-depth questions will be necessary to collect data more accurately.

These in-depth questions, which will be important when attempting to unravel the complex biological underpinnings of most phenotypes, can be asked up front for phenotypes that we suspect a priori may be challenging to assess. However, having a recontactable cohort makes the process of refinement possible when more information must be gathered. For celiac disease, starting with the question “Have you ever been diagnosed by a doctor with celiac disease?”, we replicated only one association out of almost six expected. As the prevalence of celiac disease in our cohort appeared to be somewhat higher than the reported prevalence in the United States [25], we chose to return to our customer database with a refined question of “Have you ever been diagnosed with celiac disease, as confirmed by a biopsy of the small intestine? If your diagnosis was not confirmed by a biopsy, please select no.” As a result, with a much smaller number of cases (which also reduced the number of associations we expected to replicate), we successfully replicated 4 out of 4.5 associations expected for celiac disease. This approach could also be used to examine endophenotypes or to divide broad phenotypes into subclasses with more defined characteristics.

The trend in GWAS research has been towards ever increasing sample sizes and reuse of previously genotyped cohorts whenever possible. Because it is relatively straightforward for our participants to provide information that is relevant for a variety of studies, any given individual can be a case or a control in multiple analyses at once. This could potentially reduce the total amount of work for the patient (sample collection needs to occur only once to participate in many studies) as well as potentially reducing the total number of people an investigator needs to genotype. In addition, for most conditions, this framework leads to a much larger number of controls than cases, which increases the study's power up to a certain point. Though self-report may lead to a slight increase in phenotyping error, in many cases, the lower phenotyping cost may lead to a more powerful study. For example, a study with 3,000 cases and 3,000 controls and a phenotyping error rate of 5% would have 77% power to detect a SNP at a minor allele frequency of 30% and an odds ratio of 1.3 with a p-value threshold of 10^−7^. But a study with 5,000 cases and 5,000 controls with a phenotyping error rate of 10% would have 95% power to detect such an association. Even if the error rate were 15%, the 10,000 person study would have 77% power and would have many more people to follow up with. Although more data are needed to evaluate the true costs of this model relative to other models, we believe that this method has the potential to collect high-quality phenotype data in an efficient manner.

The framework described here, in which additional questions can be directed at participants at any time with relatively low marginal effort, facilitates follow-up on specific topics as shown in the celiac disease example. Thus, one possible model for large-scale phenotyping could start with broad but shallow phenotyping by self-report on a very large cohort of individuals, followed by targeted recontact of specific subsets of individuals for deeper phenotyping based on the initial information gathered. The additional phenotyping could involve more in-depth questions to the participants or a completely different type of data collection that may require an in-person visit. A platform like this one that maintains an ongoing relationship with the participants, including sharing data with them, may motivate individuals to participate and stay active in research (for example, more than 80% of our research participants have taken more than one research survey).

There are many benefits to having a large, recontactable cohort. Testing new hypotheses, following up on initial data, and assessing the accuracy of different risk prediction models are easier when the need to assemble a new cohort every time is obviated. This raises the question, how large of a cohort is needed? With 20,000 generally unselected people, we expected to replicate approximately 40% of the associations that we tested. Only a 10× increase to 200,000 individuals would raise the expected proportion of replications to 80%, and with a million the expected replication rate would be more than 97%. A simple sum of the initial sample sizes in the papers reported in the GWAS catalog totals nearly 1,400,000. This is clearly an overestimate of the number of genotyped individuals as certain cohorts are reported in more than one study, but even if only 70% of these individuals are unique, this would constitute a resource of a million individuals with genome-wide genotype data who may be interested in participating in further research if given the opportunity. Unfortunately, because of the way research is currently done, these individuals come from dozens of different cohorts and it would be impractical if not impossible to recontact them all. As we move into studies that require ever larger sample sizes, such as those investigating gene—gene or gene—environment interactions, developing more efficient methods of conducting this type of research will become a necessity. We believe that this model in which investigators maintain long-term relationships with research participants and facilitate their participation through online tools is a significant step in that direction.

## Methods

### Cohort, Genotyping, and Phenotyping

Participants of European ancestry were drawn from the customer base of 23andMe. This group is almost 58% male, with an average age of 46 (approximately 95% of the group is between the ages of 20 and 80). Most of our participants are from the United States, with the next largest groups from Canada and Europe. Genotyping was performed on the Illumina HumanHap550+ BeadChip and all SNPs tested had a call rate of at least 99%. All individuals provided informed consent and answered surveys online according to our human subjects protocol, which was reviewed and approved by Independent Review Consulting, now part of Ethical & Independent Review Services, a private institutional review board (http://www.eandireview.com). A number of the surveys were based on existing instruments in the literature; the remainder was developed by 23andMe scientists. In general, the new questionnaires were designed in collaboration with a medical professional and reviewed by an external scientific group. All surveys were accessible to customers who had logged in to their 23andMe accounts on a page labeled “Research Surveys”. Each survey was labeled with a descriptive title and a link to a short explanation of the content and purpose of the survey. Surveys could be taken in any order and at any time. Because of this, the response rate (number of people who answered the survey divided by all genotyped users who had consented to participate in research, which is likely greater than the number of people who ever viewed the survey) varied from survey to survey but was generally in the range of 15–40%. The “Your Medical History” survey, from which the majority of the phenotype data used in the study were collected, had a response rate of 39%. We used data in our analysis that were collected prior to October 20, 2010.

### Identification of SNPs for replication studies

The GWAS catalog is a list of genome-wide association studies curated by the National Human Genome Research Institute's Office of Population Genomics [12], [13], and is a relatively complete catalog of SNPs found in published GWAS. We accessed the catalog on May 10, 2010 and sought to replicate as many SNP associations as possible from that list. We removed any SNPs from the list with reported p-values greater than 10^−7^ to limit the number of false positives we were attempting to replicate. In addition, we required that the entry had a reported odds ratio or regression coefficient and that the associations were to single SNPs, rather than haplotypes. For this analysis, as most GWAS are performed in populations of European descent, we restricted our attention to those associations reported in European populations to maximize the total number of associations we could test. We removed duplicate associations from the list, attempting to use the study with the largest total number of cases. To further avoid testing the same association twice, for SNPs that were in LD with each other (using a threshold of r^2^> = 0.1), we only picked one association, again attempting to use the study with the largest total number of cases. Where papers reported multiple SNPs in LD with each other, we chose the SNP with the smallest p-value. In cases where we did not have the reported SNP on our platform or where the SNP was not called in over 99% of our subjects, we used a proxy SNP if there was one with r^2^> = 0.5. We did not use results reported from papers that included the 23andMe database. Original data from the GWAS catalog can be found for all attempted replications with well-matching phenotypes in Table S7, and for all successful replications with less strictly matching phenotypes in Table S8.

Not all papers used the same stranding conventions, and some papers have misreported the risk allele. Therefore, we checked the stranding of the reported associations using a multi-step process. First, we confirmed that the CEU HapMap frequency information roughly matched the risk allele frequency reported in controls for all SNPs. Specifically, if the reported risk allele frequency and the HapMap frequency were both less than 0.35 or greater than 0.65, the frequency was judged to match. Unambiguous SNPs (i.e., SNPs whose two alleles are not reverse complements) with matching frequencies were judged to be correctly reported. All ambiguous SNPs and SNPs without HapMap data were checked manually in the original papers. This process turned up at least one SNP whose risk allele could not be determined from the original data (rs6457620 with rheumatoid arthritis, not replicated here).

Finally, we required that we could define cases in essentially the same manner as the original paper (using self-reported data for clinical data, where applicable). For example, we restricted our test to people in our database reporting disease onset before the age of 18 for associations to juvenile onset conditions. We also attempted to match smoking status and sex when applicable. In several cases the matching of diseases was a judgment call (for example, self-reports of hyperglycemia and type 2 diabetes were both coded as type 2 diabetes cases). For several common diseases, in an attempt to maximize power, we restricted our set of controls using incidence data for the disease. Specifically, we required controls to be at an age advanced enough that 90% of the controls would be expected never to develop the disease. See Methods S1 and Table S6 for how phenotypes were defined.

### Power calculations

We calculated power only for binary traits, using the model from Freidlin et al. [26], modified to calculate power under a one-sided test and to allow for phenotyping error (specified as the percentage of cases incorrectly classified as controls, and vice versa). We set error rates at 5% for cases, based on general evidence that misdiagnosis rates are often over 5%. For example, misdiagnosis rates have been estimated to be 30–45% for celiac disease [24], 5% for multiple sclerosis [27], between 23% and 82% for rheumatoid arthritis diagnosed by a non-rheumatologist [23], and even for cancer with biopsy there are 1.4% discrepant diagnoses when comparing the original diagnosis with a second opinion [28]. We took the error rate for controls to be the minimum of the disease prevalence and 10%. For associations where we used a proxy SNP not in complete linkage disequilibrium with the original reported SNP, the total sample size was scaled by r^2^ in the power calculation [29].

### Statistical methods

We calculated the p-values for binary associations using the score test for a logistic regression (also known as the Armitage test). Odds ratio (OR) and effect sizes are specified for the risk allele reported in the GWAS catalog [12], [13]. For non-binary traits, we used the Wald test for a linear regression. We used a threshold of 0.05 for significance of any individual test. All tests were one-sided in the direction of the published OR. Using different thresholds did not change the results substantially (Table S5). There is no substantial multiple testing burden in this study, as the vast majority of the associations are probably true signals.

For the prediction intervals in Figure 2, we used a model in which each attempted replication was considered to be an independent Bernoulli event with success probability equal to our estimated power for replicating that association. Using a dynamic programming recurrence, we explicitly computed the probability distribution over the total number of successful replications based on this model. We then determined a 95% prediction interval [L, U] for the total number of successful replications by finding the largest L such that the probability of observing fewer than L replications (or analogously, the smallest U such that the probability of observing greater than U replications) was at most 2.5%. Finally, we determined the reported prediction intervals by dividing these lower and upper bounds by the expected total number of successful replications.

To test whether having seen personal risk estimates for a disease had an effect on self-report of that disease, we looked for an interaction between reported disease risk and whether the individual had possibly seen their report before answering the question (Table S4). This was possible as many people filled out surveys before their results became available. More precisely, we regressed reported phenotype on predicted risk, a “results available” indicator variable, the interaction of these two variables, and age, sex and five principal components of ancestry, and tested the interaction term for significance.

## Supporting Information

Figure S1 **Success rate (versus total power) by disease.** Replications = number of associations we successfully replicated. Expected = number of associations we expected to replicate. Attempts = number of associations we attempted to replicate. The blue dot represents our success ratio (number of successful replications divided by number of expected replications). The black line represents the 95% prediction interval for the success ratio. For ovarian cancer, the success ratio is 12.8 (not within the scale of the graph).
(DOCX)

Methods S1 **Survey text.** Unless otherwise noted, if multiple questions were asked, any subject who answered positively (bolded answer) to at least one was included as a case. Controls were those who answered negatively to these questions. Individuals who gave neither an affirmative nor a negative reply (such as “I'm not sure” or “Decline to state”) were not included in the analysis. A subject who answered questions inconsistently (for example, yes to one breast cancer question, no to another) was removed from that analysis. Questions marked with “RS” were asked as Research Snippets, which are questions that are asked singly, as opposed to being part of a larger survey. For additional parameters, see Table S6.
(DOCX)

Table S1 **All binary replications attempted.** Risk = risk allele for original SNP. Chr = chromosome. log(OR) = 23andMe log odds ratio. Pub log(OR) = Published log odds ratio. Rep = replicated.
(DOCX)

Table S2 **All quantitative replications attempted.** Risk = risk allele for original SNP. Size = number of participants. Chr = chromosome. Beta = 23andMe beta (effect size). Rep = replicated.
(DOCX)

Table S3 **All successful replications without strictly matching phenotypes.** Phenotype = 23andMe phenotype. Published = published phenotype. OR = 23andMe odds ratio. ^a^ Note that this replication is in the opposite direction from that reported in the GWAS catalog. However, a close reading of the original report shows that the direction of the effect was misreported in the GWAS catalog.
(DOCX)

Table S4 **Effect of viewing genetic risk data on reported disease status.** Because one aspect of 23andMe's Personal Genome Service involves returning genetic data to our customers, it is possible that viewing a result of elevated risk for a certain disease may make it more likely for an individual to recall a previous diagnosis of that disease, thus potentially skewing the results towards replication. To address this, we investigated the impact of seeing genetic risk results before versus after answering survey questions on self-reported disease status for a set of 20 conditions for which participants were able to view a personal risk prediction. Only for psoriasis was there a statistically significant impact of seeing one's results on self-report of disease status, and this impact was no longer observed once the direction of the estimated risk (increased or decreased) was taken into account, suggesting that in general, the nature of the genetic risk result did not have a consistent or significant effect on the way questions were answered. Estimated Risk = p-value for association of estimated risk with reported disease status. We expect to see an association with any risk model that is reasonably predictive. Saw Data First = p-value for association of viewing genetic risk results before answering survey questions with reported disease status. Estimated Risk * Saw Data First = p-value for association of interaction between estimated risk and viewing this risk before answering survey questions with reported disease status.
(DOCX)

Table S5 **Success rate by replication p-value threshold.** Alpha = p-value threshold for replication. Replications = number of associations successfully replicated. Expected = number of associations we expected to replicate. Ratio = expected / replications. These calculations do not include the nine associations for which our power may be substantially overestimated.
(DOCX)

Table S6 **Additional parameters for phenotype classification.**
(DOCX)

Table S7 **Data from GWAS catalog for associations with strictly matching phenotypes.** PMID: PubMed ID. RAF = risk allele frequency. OR/Beta = odds ratio or beta (effect size). CI = confidence interval.
(DOCX)

Table S8 **Data from GWAS catalog for successful associations without strictly matching phenotypes.** PMID: PubMed ID. RAF = risk allele frequency. OR/Beta = odds ratio or beta (effect size). CI = confidence interval.
(DOCX)
