# Supplementary figures and images for: Efficient Replication of over 180 Genetic Associations with Self-Reported Medical Data
Source: PLoS One. 2011 Aug 17;6(8):e23473. doi: 10.1371/journal.pone.0023473 (PMC3157390; doi:10.1371/journal.pone.0023473)

**Figure S1**


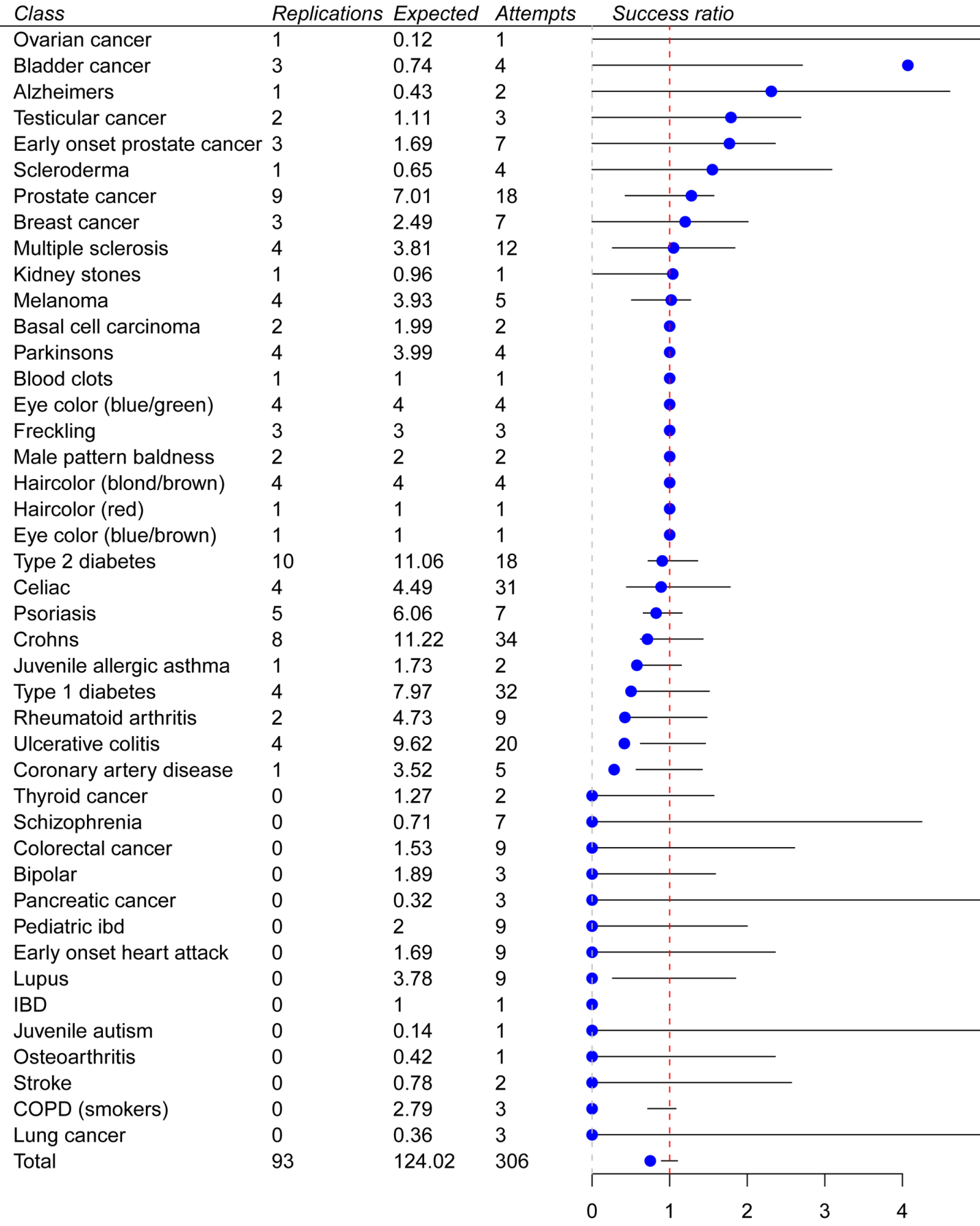

Supplement: Figure S1 — Success rate (versus total power) by disease. Replications = number of associations we successfully replicated. Expected = number of associations we expected to replicate. Attempts = number of associations we attempted to replicate. The blue dot represents our success ratio (number of successful replications divided by number of expected replications). The black line represents the 95% prediction interval for the success ratio. For ovarian cancer, the success ratio is 12.8 (not within the scale of the graph). (DOCX) [file pone.0023473.s001.docx]
