# Supplementary material for: Efficient Replication of over 180 Genetic Associations with Self-Reported Medical Data
Source: PLoS One. 2011 Aug 17;6(8):e23473. doi: 10.1371/journal.pone.0023473 (PMC3157390; doi:10.1371/journal.pone.0023473)
Supplement: Methods S1 — Survey text. Unless otherwise noted, if multiple questions were asked, any subject who answered positively (bolded answer) to at least one was included as a case. Controls were those who answered negatively to these questions. Individuals who gave neither an affirmative nor a negative reply (such as “I'm not sure” or “Decline to state”) were not included in the analysis. A subject who answered questions inconsistently (for example, yes to one breast cancer question, no to another) was removed from that analysis. Questions marked with “RS” were asked as Research Snippets, which are questions that are asked singly, as opposed to being part of a larger survey. For additional parameters, see Table S6. (DOCX) [file pone.0023473.s002.docx]

**SUPPORTING METHODS S1**

**Survey text**

Alcohol abuse

- Have you ever had any of the following types of *substance abuse* problems? –Alcoholism
  - **Yes**
  - No
  - I’m not sure
- How often do/did you drink? (One drink = one can of beer, one glass of wine, one shot, or one mixed drink)
  - 0-3 drinks/week
  - 4-6 drinks/week
  - 1-2 drinks/day
  - **3-6 drinks/day**
  - **More than 6 drinks/day)**
- Was there ever a 12 month period in your life in which you drank two or more drinks a day, seven days a week?
  - **Yes**
  - No
  - Decline to state

Allergic asthma, juvenile onset

- Have you ever had an asthma attack?
  - **Yes**
  - No
  - I’m not sure
- When did you experience your asthma attack(s)?
  - **In childhood only (only experienced asthma at age 17 or younger, then grew out of it)**
  - In adulthood only (only developed asthma at age 18 or older)
  - **In both childhood and adulthood**
- What triggered your asthma attack(s)?
  - Air pollution
  - Cigarette smoke
  - **Cleaning products**
  - Cold, flu, bronchitis, or sinus infection
  - **Dust**
  - Exercise
  - **Food**
  - Heartburn or acid reflux
  - **Perfumes**
  - **Seasonal asthma**
  - **Weather changes**
  - Something else
  - I’m not sure
- *Scoring: Cases = must respond positively to all three questions*

Alzheimer’s disease

- Have you ever been diagnosed by a doctor with any of the following *neurological* conditions? –Alzheimer’s disease
  - **Yes**
  - No
  - I’m not sure

Autism

- Have you ever been diagnosed with any of the following *psychiatric* conditions? –Autism-spectrum disorder (for example, Asperger syndrome, pervasive developmental disorder, or autism)
  - **Yes**
  - No
  - I’m not sure
- At what age were you first diagnosed with an autism-spectrum disorder (for example, Asperger syndrome, pervasive developmental disorder, or autism)? Your best guess is fine. (**age <= 18**)
- *Scoring: Cases = must respond positively to both questions*

Basal cell carcinoma

- Have you ever been diagnosed by a doctor with any of the following *common cancers*? –Basal cell carcinoma
  - **Yes**
  - No
  - I’m not sure
- RS: Have you ever been diagnosed with basal cell carcinoma?
  - **Yes**
  - No
  - I’m not sure

Bipolar disorder

- Have you ever been diagnosed by a doctor with any of the following *psychiatric* conditions? –Bipolar disorder (manic depression)
  - **Yes**
  - No
  - I’m not sure
- RS: Have you ever been diagnosed with manic depression (sometimes also called bipolar disorder)?
  - **Yes**
  - No
  - I’m not sure

Birth weight

- How much did you weigh at birth? (*Dropdown* a*nswer in pounds and ounces, or grams*)

Bladder cancer

- Have you ever been diagnosed by a doctor with any of the following *common cancers*? –Bladder cancer
  - **Yes**
  - No
  - I’m not sure
- RS: Have you ever been diagnosed with bladder cancer?
  - **Yes**
  - No
  - I’m not sure

Blood clots

- Have you ever been diagnosed by a doctor with any of the following *blood* conditions? –Blood clots
  - **Yes**
  - No
  - I’m not sure

Body mass index (BMI)

- Height: (*Dropdown with feet and inches, or meters*); Weight (in pounds): (*Free fill integer field*)
- *Scoring: BMI = (weight / 2.2) / (height / 39.37)^2^*

Breast cancer

- Have you ever been diagnosed with any of the following *common cancers*? –Breast cancer
  - **Yes**
  - No
  - I’m not sure
- RS: Have you ever been diagnosed with breast cancer?
  - **Yes**
  - No
  - I’m not sure

Coronary artery disease

- Have you ever been diagnosed by a doctor with any of the following *cardiovascular* conditions? –Coronary artery disease/heart disease
  - **Yes**
  - No
  - I’m not sure

Celiac disease

- RS: Have you ever been diagnosed with celiac disease, as confirmed by a biopsy of the small intestine? If your diagnosis was not confirmed by a biopsy, please select no.
  - **Yes**
  - No
  - I’m not sure

Colorectal cancer

- Have you ever been diagnosed with any of the following *common cancers*? –Colorectal cancer
  - **Yes**
  - No
  - I’m not sure
- RS: Have you ever been diagnosed with colorectal cancer (colon cancer and/or rectal cancer)?
  - **Yes**
  - No
  - I’m not sure

Chronic obstructive pulmonary disease (COPD)

- Have you ever been diagnosed by a doctor with any of the following *infectious* conditions? –Chronic bronchitis
  - **Yes**
  - No
  - I’m not sure
- Have you ever been diagnosed with any of the following *other* conditions? –Emphysema
  - **Yes**
  - No
  - I’m not sure

Crohn’s disease

- Have you ever been diagnosed with any of the following *intestinal* conditions? –Crohn’s disease
  - **Yes**
  - No
  - I’m not sure
- RS: Have you ever been diagnosed with Crohn’s disease?
  - **Yes**
  - No
  - I’m not sure

Eye color (blue v brown)

- Look very closely at the color of your eyes, preferably under bright natural light. Then complete the following – the image that most closely resembles the color of my eyes is:
  -
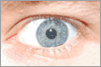
 (*coded as blue*)
  -
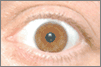
 (*coded as brown*)
  -
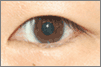
 (*coded as brown*)

Freckling

- The freckling (not moles – moles can be present at birth and can form even in parts of the body not exposed to the sun) on my face is most similar to:
  -
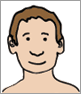
 (*coded as 0)*
  - *
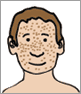
 (coded as 1)*
  - *
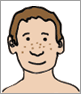
 (coded as 2)*
  - *
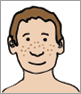
 (coded as 3)*
  - *
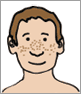
 (coded as 4)*
  - *
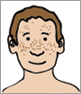
 (coded as 5)*
  - *
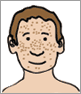
 (coded as 6)*
- The freckling (not moles) on my arms is most similar to:
  -
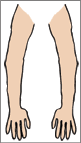
 *(coded as 0)*
  -
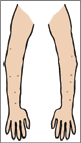
 *(coded as 1)*
  -
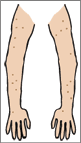
 *(coded as 2)*
  -
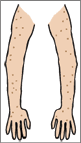
 *(coded as 3)*
  -
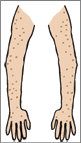
 *(coded as 4)*
  -
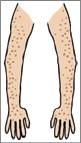
 *(coded as 5)*
- The freckling (not moles) on my shoulders is most similar to:
  -
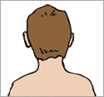
 *(coded as 0)*
  -
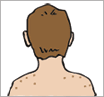
 *(coded as 1)*
  -
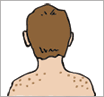
 *(coded as 2)*
  -
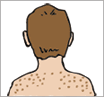
 (*coded as 3)*
  -
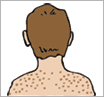
 *(coded as 4)*
  -
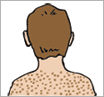
 *(coded as 5)*
- *Scoring: scores from the three questions were summed. Case = 3 and above; Control = 0 – 2*

Gall bladder removal

- Have you ever had any of the following *gastrointestinal* surgeries? –Gall bladder removal
  - **Yes**
  - No
  - I’m not sure

Hair curl

- Is your hair naturally straight or curly?
  -
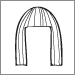
 *(coded as 0)*
  -
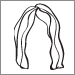
 *(coded as 1)*
  -
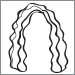
 *(coded as 2)*
  -
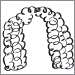
 *(coded as 3)*
  -
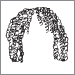
 *(coded as 4)*
  -
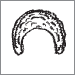
 *(coded as 5)*

Hair color (blond v brown)

- The natural color of my hair (before I went gray, if I am gray now) is most similar to:
  -
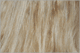
 *(coded as blond)*
  -
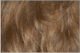
 *(coded as blond)*
  -
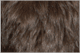
 *(coded as brown)*
  -
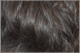
 *(coded as blond)*

Hair color (quantitative, including all colors)

- The natural color of my hair (before I went gray, if I am gray now) is most similar to:
  -
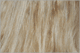
 *(coded as 1)*
  - *
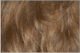
 (coded as 3)*
  - *
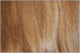
 (coded as 2)*
  - *
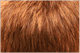
 (coded as 0)*
  - *
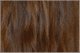
 (coded as 5)*
  - *
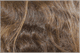
 (coded as 4)*
  - *
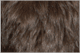
 (coded as 6)*
  - *
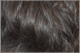
 (coded as 7)*
  - *
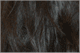
 (coded as 8)*

Hair color (quantitative, not including red)

- The natural color of my hair (before I went gray, if I am gray now) is most similar to:
  -
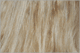
 *(coded as 0)*
  - *
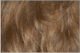
 (coded as 1)*
  - *
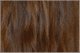
 (coded as 3)*
  - *
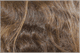
 (coded as 2)*
  - *
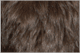
 (coded as 4)*
  - *
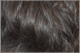
 (coded as 5)*
  - *
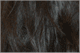
 (coded as 6)*

Hair color (red v not red)

- The natural color of my hair (before I went gray, if I am gray now) is most similar to:
  -
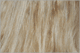
 *(coded as not red)*
  - *
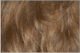
 (coded as not red)*
  - *
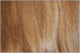
 (coded as red)*
  - *
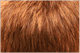
 (coded as red)*
  - *
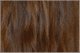
 (coded as red)*
  - *
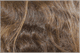
 (coded as not red)*
  - *
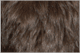
 (coded as not red)*
  - *
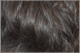
 (coded as not red)*
  - *
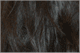
 (coded as not red)*

Height

- Height: (*Dropdown with feet and inches or meters*)

Heart attack

- Have you ever been diagnosed by a doctor with any of the following *cardiovascular* conditions? –Heart attack
  - **Yes**
  - No
  - I’m not sure

High blood pressure

- Have you ever been diagnosed by a doctor with any of the following *cardiovascular* conditions? –High blood pressure
  - **Yes**
  - No
  - I’m not sure

High cholesterol

- Have you ever been diagnosed by a doctor with any of the following *cardiovascular* conditions? –High cholesterol (over 200mg/dl) or Hypercholesterolemia
  - **Yes**
  - No
  - I’m not sure

Inflammatory bowel disease (IBD)

- Have you ever been diagnosed with any of the following *intestinal* conditions? –Crohn’s disease
  - **Yes**
  - No
  - I’m not sure
- Have you ever been diagnosed with any of the following *intestinal* conditions? –Ulcerative colitis
  - **Yes**
  - No
  - I’m not sure

Kidney stones

- Have you ever been diagnosed by a doctor with any of the following *kidney or urinary tract* conditions? –Kidney stones
  - **Yes**
  - No
  - I’m not sure
- RS: Have you ever been diagnosed with kidney stones?
  - **Yes**
  - No
  - I’m not sure

Liver test

- Have you ever been diagnosed by a doctor with any of the following *liver* conditions? –Elevated result on liver function test
  - **Yes**
  - No
  - I’m not sure

Lung cancer

- Have you ever been diagnosed by a doctor with any of the following *common cancers*? –Lung cancer
  - **Yes**
  - No
  - I’m not sure

Lupus (Systemic lupus erythematosus)

- Have you ever been diagnosed by a doctor with any of the following *autoimmune* conditions? –Lupus
  - **Yes**
  - No
  - I’m not sure
- RS: Have you ever been diagnosed with systemic lupus erythematosus (SLE)?
  - **Yes**
  - No
  - I’m not sure

Macular degeneration

- Have you ever been diagnosed by a doctor with any of the following *vision* conditions? –Macular degeneration
  - **Yes**
  - No
  - I’m not sure

Male pattern baldness

- Have you experienced hair loss or thinning?
  - **Yes**
  - No
  - I’m not sure
  - Decline to state
- Please choose the image that best captures your hair’s pattern and density. If your head is shaved, please answer for how your hair looks when grown out. If none of these images are similar to your hair’s pattern and density, choose none of the above.


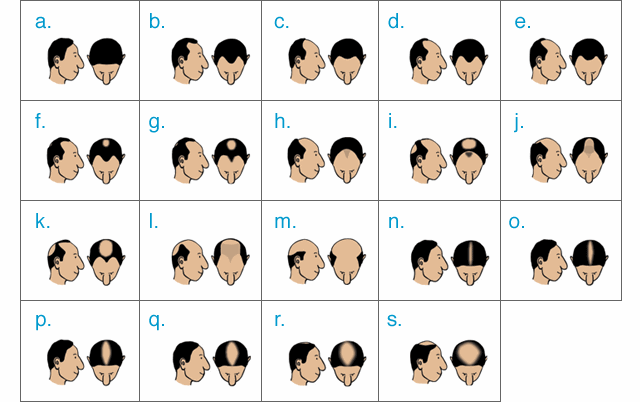


- *Scoring: Case = yes have experienced hair loss or thinning and chose an image from the set {b, m}; Control = no have not experienced hair loss or thinning and chose image a.*

Melanoma

- Have you ever been diagnosed by a doctor with any of the following *common cancers*? –Melanoma
  - **Yes**
  - No
  - I’m not sure
- RS: Have you ever been diagnosed with melanoma?
  - **Yes**
  - No
  - I’m not sure

Multiple sclerosis

- Have you ever been diagnosed by a doctor with any of the following *autoimmune* conditions? –Multiple sclerosis
  - **Yes**
  - No
  - I’m not sure
- RS: Do you have multiple sclerosis?
  - **Yes**
  - No
  - I’m not sure

Nicotine abuse

- Have you ever had any of the following types of *substance abuse* problems? –Nicotine abuse
  - **Yes**
  - No
  - I’m not sure

Osteoarthritis

- Have you ever been diagnosed by a doctor with any of the following *bone or joint* conditions? –Osteoarthritis
  - **Yes**
  - No
  - I’m not sure

Osteoporosis

- Have you ever been diagnosed by a doctor with any of the following *bone or joint* conditions? –Osteoporosis
  - **Yes**
  - No
  - I’m not sure

Ovarian cancer

- Have you ever been diagnosed by a doctor with any of the following *women-only cancers*? –Ovarian cancer
  - **Yes**
  - No
  - I’m not sure

Pancreatic cancer

- Have you ever been diagnosed by a doctor with any of the following *common cancers*? –Pancreatic cancer
  - **Yes**
  - No
  - I’m not sure

Parkinson’s disease

- (1) Have you ever been diagnosed by a doctor with any of the following *neurological* conditions? –Parkinson’s disease
  - Yes
  - No
  - I’m not sure
- (2) Has a doctor ever diagnosed you with Parkinson’s disease?
  - Yes
  - No
  - I’m not sure/I don’t remember
- (3) Has a doctor ever diagnosed you with any of the following? Please check all that apply.
  - Another form of parkinsonism (other than Parkinson’s disease)
  - Dementia, cognitive impairment, or senility
  - A tremor disorder
  - None of the above
- (4) Is your diagnosis still Parkinson’s disease or has your diagnosis changed?
  - My diagnosis is still Parkinson’s disease
  - My diagnosis changed
  - I’m not sure/I don’t remember
- (5) Which of the following best describes how your diagnosis changed?
  - I no longer have the symptoms that led to the Parkinson’s disease diagnosis
  - I still have the symptoms, but the cause is unknown
  - I was diagnosed with a different disease
- (6) Which of the following have you been diagnosed with? Please check all that apply.
  - Progressive supranuclear palsy (PSP)
  - Multiple system atrophy (MSA), Shy-Drager syndrome, or striatonigral degeneration
  - Spinal cerebellar ataxia (SCA), cerebellar degeneration, olivopontocerebellar atrophy (OPCA)
  - Cortical basal ganglionic degeneration
  - Atypical parkinsonism or Parkinson's plus
  - Genetic or familial parkinsonism
  - Parkinsonism due to medications, drug-induced parkinsonism
  - Alzheimer's disease with parkinsonism
  - Frontotemporal dementia with parkinsonism
  - Parkinson's disease dementia (PDD)
  - Dementia with Lewy bodies (DLB), Lewy body dementia or cortical Lewy body disease
  - Another form of dementia
  - Vascular parkinsonism
  - None of the above
- (7) Which of the following have you been diagnosed with? Please check all that apply.
  - Alzheimer's disease
  - Dementia with Lewy bodies (DLB), Lewy body dementia or cortical Lewy body disease
  - Frontotemporal dementia (FTD)
  - Parkinson's disease dementia (PDD)
  - Vascular dementia
  - Picks disease
  - Mild cognitive impairment (MCI)
  - Another form of dementia
  - None of the above
- (8) Which of the following have you been diagnosed with? Please check all that apply.
  - Essential tremor (ET), benign tremor, or senile tremor
  - Dystonic tremor
  - Rubral tremor
  - Primary writing tremor
  - Tremor due to a stroke
  - Cerebellar tremor
  - Multiple sclerosis (MS)
  - Another tremor disorder
  - None of the above
- (9) Did your diagnosis change to any of the following? Please check all that apply.
  - Progressive supranuclear palsy (PSP)
  - Multiple system atrophy (MSA), Shy-Drager syndrome, or striatonigral degeneration
  - Spinal cerebellar ataxia (SCA), cerebellar degeneration, olivopontocerebellar atrophy (OPCA)
  - Cortical basal ganglionic degeneration
  - Atypical parkinsonism or Parkinson's plus
  - Genetic or familial parkinsonism
  - Parkinsonism due to medications, drug-induced parkinsonism
  - Alzheimer's disease with parkinsonism
  - Frontotemporal dementia with parkinsonism
  - Parkinson's disease dementia (PDD)
  - Dementia with Lewy bodies (DLB), Lewy body dementia or cortical Lewy body disease
  - Another form of dementia
  - Vascular parkinsonism
  - None of the above
- (10) Did your diagnosis change to any of the following? Please check all that apply.
  - Alzheimer's disease
  - Dementia with Lewy bodies (DLB), Lewy body dementia or cortical Lewy body disease
  - Frontotemporal dementia (FTD)
  - Parkinson's disease dementia (PDD)
  - Vascular dementia
  - Picks disease
  - Mild cognitive impairment (MCI)
  - Another form of dementia
  - None of the above
- (11) Did your diagnosis change to any of the following? Please check all that apply.
  - Essential tremor (ET), benign tremor, or senile tremor
  - Dystonic tremor
  - Rubral tremor
  - Primary writing tremor
  - Tremor due to a stroke
  - Cerebellar tremor
  - Multiple sclerosis (MS)
  - Another tremor disorder
  - None of the above
- (12) Did your diagnosis change to any of the following? Please check all that apply.
  - Motor neuron disease (MND), amyotrophic lateral sclerosis (ALS), Lou Gehrig's disease
  - Spinal muscular atrophy (SMA), Progressive muscular atrophy (PMA), primary lateral sclerosis (PLS)
  - Spinal cerebellar ataxia (SCA), cerebellar degeneration, olivopontocerebellar atrophy (OPCA)
  - Multiple sclerosis (MS)
  - Stroke or cerebrovascular accident (CVA)
  - Epilepsy, seizure disorder
  - Myoclonus
  - Dystonia
  - Tourette syndrome, tic disorder
  - Another brain or neurological disorder
  - Parkinsonism due to medications, drug-induced parkinsonism
  - Depression
  - Thyroid disease
  - Arthritis
  - Benign positional vertigo, vertigo, balance problems due to the inner ear, or vestibular disease
  - Another medical problem
  - None of the above
- (13) Have you ever been diagnosed with any of the following *neurological* conditions? (Alzheimer’s disease)
- (14) Have you ever been diagnosed with any of the following *neurological* conditions? (Memory loss)
- (15) Do you have any trembling or shaking of any part of your body?
  - Yes *(coded as +1)*
  - No
  - I’m not sure/I don’t remember
- (16) Have you noticed any of the following changes in your handwriting? Please check all that apply.
  - Handwriting became slower *(coded as +1)*
  - Handwriting became smaller *(coded as +1)*
  - Handwriting became shakier *(coded as +1)*
- (17) Have you noticed or have people told you that your speech or voice has become softer than before?
  - Yes *(coded as +1)*
  - No
  - I’m not sure/I don’t remember
- (18) Have you noticed any of the following changes in the way your walk? Please check all that apply.
  - Dragging one or both feet *(coded as +1)*
  - Feet shuffling *(coded as +1)*
  - Walking more slowly *(coded as +1)*
  - Taking smaller steps than before *(coded as +1)*
  - Steps becoming faster and faster *(coded as +1)*
  - Feet getting stuck as if glued to the floor *(coded as +1)*
  - None of the above
- (19) When walking, do you swing your arms less than you used to?
  - Yes *(coded as +1)*
  - No
  - I’m not sure/I don’t remember
- (20) When standing or walking do you stoop or bend forward more than you used to?
  - Yes *(coded as +1)*
  - No
  - I’m not sure/I don’t remember
- (21) Do you have trouble with your balance or fall sometimes?
  - Yes *(coded as +1)*
  - No
  - I’m not sure/I don’t remember
- (22) Has a doctor ever diagnosed any of your *biological* family members with Parkinson’s disease? Please check all that apply.
  - Mother
  - Father
  - Sister
  - Brother
  - Daughter
  - Son
  - Maternal grandparent
  - Paternal grandparent
  - Maternal aunt or uncle
  - Paternal aunt or uncle
  - Maternal first cousin
  - Paternal first cousin
  - None of the above
- *Scoring: Because many of the individuals with Parkinson’s disease were recruited as part of a special Parkinson’s disease research community project, most of them were given a special tag in the database (“PD”) to record how they were recruited. Family members of these individuals were also specially tagged (“pd.family”). Cases = Anyone who reported a diagnosis of Parkinson’s disease in question 1 or 2 and did not say no to either question 1 or 2. Anyone who said his/her diagnosis changed (question 4) because they no longer have symptoms or the cause is unknown (question 5) was not included as a case. Anyone who said his/her diagnosis changed to a different disease, said yes to question 1 and I’m not sure to question 2, and reported a diagnosis of progressive supranuclear palsy, multiple system atrophy (or Shy-Drager syndrome or striatonigral degeneration), spinal cerebellar ataxia (or cerebellar degeneration or olivopontocerebellar atrophy), cortical basal ganglionic degeneration, parkinsonism due to medications (or drug-induced parkinsonism), frontotemporal dementia with parkinsonism, frontotemporal dementia, Picks disease, dystonic tremor, rubral tremor, primary writing tremor, cerebellar tremor, or multiple sclerosis (questions 6, 7, and 8) was not included as a case. Anyone who said his/her diagnosis changed (question 4) and reported a new diagnosis of progressive supranuclear palsy, multiple system atrophy (or Shy-Drager syndrome or striatonigral degeneration), spinal cerebellar ataxia (or cerebellar degeneration or olivopontocerebellar atrophy), cortical basal ganglionic degeneration, parkinsonism due to medications (or drug-induced parkinsonism), frontotemporal dementia with parkinsonism, frontotemporal dementia, Picks disease, dystonic tremor, rubral tremor, primary writing tremor, cerebellar tremor, multiple sclerosis, motor neuron disease (or amyotrophic lateral sclerosis or Lou Gehrig’s disease), spinal muscular atrophy (or progressive muscular atrophy or primary lateral sclerosis), epilepsy (or seizure disorder), myoclonus, dystonia, or Tourette syndrome (or tic disorder) (questions 9, 10, 11, and 12) was not included as a case. Controls = Anyone who: did not have a PD tag or a pd.family tag, affirmatively reported no diagnosis of Parkinson’s disease in question 1 and question 2, did not report a diagnosis of parkinsonism or dementia or tremor in question 3, did not report a diagnosis of Alzheimer’s disease or memory loss in questions 13 and 14, did not report any family history of Parkinson’s disease (question 22), and was age 50 or above. In addition, the number of Parkinson’s disease-like symptoms was summed across questions 15-21 and anyone with a symptom score of 3 or greater was not included as a control.*

Prostate cancer

- Have you ever been diagnosed with any of the following *men-only cancers*? –Prostate cancer
  - **Yes**
  - No
  - I’m not sure
- RS: Have you ever been diagnosed with prostate cancer?
  - **Yes**
  - No
  - I’m not sure

Psoriasis

- Have you ever been diagnosed by a doctor with any of the following *autoimmune* conditions? –Psoriasis
  - **Yes**
  - No
  - I’m not sure
- RS: Have you ever been diagnosed with psoriasis?
  - **Yes**
  - No
  - I’m not sure

Rheumatoid arthritis

- Have you ever been diagnosed by a doctor with any of the following *autoimmune* conditions? –Rheumatoid arthritis
  - **Yes**
  - No
  - I’m not sure
- RS: Have you ever been diagnosed with rheumatoid arthritis?
  - **Yes**
  - No
  - I’m not sure

Schizophrenia

- Have you ever been diagnosed by a doctor with any of the following *psychiatric* conditions? –Schizophrenia
  - **Yes**
  - No
  - I’m not sure

Scleroderma

- Have you ever been diagnosed by a doctor with any of the following *autoimmune* conditions? –Scleroderma
  - **Yes**
  - No
  - I’m not sure

Stroke

- Have you ever been diagnosed by a doctor with any of the following *cardiovascular* conditions? –Stroke
  - **Yes**
  - No
  - I’m not sure

Type 1 diabetes

- Have you ever been diagnosed by a doctor with any of the following *diabetes and diabetes-related* conditions? –Type 1 diabetes
  - **Yes**
  - No
  - I’m not sure
- RS: Do you have type 1 diabetes (sometimes called juvenile diabetes)?
  - **Yes**
  - No
  - I’m not sure

Type 2 diabetes

- Have you ever been diagnosed by a doctor with any of the following *diabetes and diabetes-related* conditions? –Type 2 diabetes
  - **Yes**
  - No
  - I’m not sure
- Have you ever been diagnosed by a doctor with any of the following *diabetes and diabetes-related* conditions? –Gestational diabetes
  - **Yes**
  - No
  - I’m not sure
- Have you ever been diagnosed by a doctor with any of the following *diabetes and diabetes-related* conditions? –High blood sugar (hyperglycemia)
  - **Yes**
  - No
  - I’m not sure

Tanning

- Does your skin become tan after prolonged sun exposure without sun protection?
  - No, my skin always or almost always burns and never tans *(coded as 1)*
  - Yes, my skin typically burns during the first or first few exposures, and then tans *(coded as 2)*
  - Yes, my skin tans a little *(coded as 3)*
  - Yes, my skin tans moderately *(coded as 4)*
  - Yes, my skin tans easily *(coded as 5)*
  - I'm not sure
- How does your skin initially react to prolonged sun exposure without sun protection?
  - Painful, red blistering, then peeling *(coded as 1)*
  - Blistering followed by peeling *(coded as 2)*
  - Burns sometimes followed by peeling *(coded as 3)*
  - Rarely burns *(coded as 4)*
  - Never burns *(coded as 5)*
  - I'm not sure
- What is the darkest your skin will get from repeated sun exposure without sun protection?
  - Little or no change *(coded as 1)*
  - Light color tan *(coded as 2)*
  - Medium tan *(coded as 3)*
  - Dark tan *(coded as 4)*
  - I'm not sure
- If you don't already have a tan, will your skin become tan within several hours of being exposed to the sun?
  - No, never *(coded as 1)*
  - Yes, but rarely *(coded as 2)*
  - Yes, sometimes *(coded as 3)*
  - Yes, frequently *(coded as 4)*
  - Yes, always or almost always *(coded as 5)*
  - I'm not sure
- *Scoring: scores from summed across all four questions (higher number means more tanning, less burning)*

Testicular cancer

- Have you ever been diagnosed by a doctor with any of the following *men-only* cancers? –Testicular cancer
  - **Yes**
  - No
  - I’m not sure

Thyroid cancer

- Have you ever been diagnosed by a doctor with any of the following *common cancers*? –Thyroid cancer
  - **Yes**
  - No
  - I’m not sure
- RS: Have you ever been diagnosed with thyroid cancer?
  - **Yes**
  - No
  - I’m not sure

Ulcerative colitis

- Have you ever been diagnosed by a doctor with any of the following *intestinal* conditions? –Ulcerative colitis
  - **Yes**
  - No
  - I’m not sure
- RS: Have you ever been diagnosed with ulcerative colitis?
  - **Yes**
  - No
  - I’m not sure

**Disease class assignments**

**Asthma**: childhood asthma

**Autoimmune**: Crohn’s disease, inflammatory bowel disease, lupus, multiple sclerosis, psoriasis, type 1 diabetes, ulcerative colitis

**Cancer** : basal cell carcinoma, bladder cancer, breast cancer, colorectal cancer, prostate cancer, lung cancer, melanoma, pancreatic cancer, scleroderma, testicular cancer, thyroid cancer

**Celiac**: celiac disease

**Diabetes**: type 2 diabetes

**Heart**: blood clots, coronary artery disease, heart attack

**Pigment/Hair**: eye color, freckling, hair color, red hair color, male pattern baldness

**Neuro** : Alzheimer’s disease, autism, Parkinson’s disease

**Other**: chronic obstructive pulmonary disease, kidney stones, stroke, osteoarthritis

**Psychiatric**: alcohol abuse, bipolar disorder, schizophrenia
