# Supplementary material for: Efficient Replication of over 180 Genetic Associations with Self-Reported Medical Data
Source: PLoS One. 2011 Aug 17;6(8):e23473. doi: 10.1371/journal.pone.0023473 (PMC3157390; doi:10.1371/journal.pone.0023473)
Supplement: Table S1 — All binary replications attempted. Risk = risk allele for original SNP. Chr = chromosome. log(OR) = 23andMe log odds ratio. Pub log(OR) = Published log odds ratio. Rep = replicated. (DOCX) [file pone.0023473.s003.docx]

**Table S1**

| **Phenotype** | **SNP** | **Risk** | **Proxy SNP** | **P-value** | **Cases** | **Cont** | **Chr** | **Region** | **log (OR)** | **Lower CI** | **Upper CI** | **Pub log (OR)** | **Rep** |
| --- | --- | --- | --- | --- | --- | --- | --- | --- | --- | --- | --- | --- | --- |
| Alcohol abuse | rs7590720 | G | n/a | 0.875 | 1811 | 8549 | 2 | PECR | -0.046 | -0.128 | 0.030 | 0.300 | No |
| Alzheimer's disease | rs2075650 | G | n/a | 0.0434 | 11 | 82 | 19 | APOE | 0.933 | -0.186 | 2.050 | 0.928 | Yes |
| Alzheimer's disease | rs3851179 | C | n/a | 0.66 | 11 | 82 | 11 | PICALM | -0.182 | -1.089 | 0.734 | 0.148 | No |
| Basal cell carcinoma | rs7538876 | A | n/a | 1.17E-05 | 1176 | 4826 | 1 | PADI4 | 0.199 | 0.105 | 0.288 | 0.247 | Yes |
| Basal cell carcinoma | rs801114 | G | n/a | 0.0103 | 1176 | 4826 | 1 | RHOU | 0.111 | 0.020 | 0.198 | 0.247 | Yes |
| Bipolar disorder | rs10994336 | T | rs4948418 | 0.297 | 366 | 13030 | 10 | ANK3 | 0.080 | -0.211 | 0.372 | 0.372 | No |
| Bipolar disorder | rs1006737 | A | rs2159100 | 0.475 | 366 | 13030 | 12 | CACNA1C | 0.005 | -0.151 | 0.166 | 0.166 | No |
| Bipolar disorder | rs1012053 | A | n/a | 0.485 | 366 | 13030 | 13 | DGKH | 0.004 | -0.199 | 0.198 | 0.464 | No |
| Bipolar disorder | rs420259 | A | n/a | 0.659 | 366 | 13030 | 16 | PALB2 | -0.034 | -0.199 | 0.128 | 0.732 | No |
| Bipolar disorder | rs17418283 | C | rs166241 | 0.764 | 366 | 13030 | 5 | MCTP1 | -0.059 | -0.223 | 0.104 | 0.191 | No |
| Bladder cancer | rs710521 | T | n/a | 0.0282 | 47 | 11235 | 3 | TP63 | 0.502 | -0.020 | 1.030 | 0.174 | Yes |
| Bladder cancer | rs9642880 | T | n/a | 0.0281 | 47 | 11235 | 8 | MYC | 0.398 | -0.010 | 0.806 | 0.199 | Yes |
| Bladder cancer | rs2294008 | T | n/a | 0.0153 | 47 | 11235 | 8 | PSCA | 0.447 | 0.039 | 0.854 | 0.140 | Yes |
| Bladder cancer | rs798766 | T | n/a | 0.407 | 47 | 11235 | 4 | TACC3 | 0.059 | -0.431 | 0.554 | 0.215 | No |
| Blood clots | rs505922 | C | n/a | 3.42E-07 | 274 | 10838 | 9 | ABO | 0.431 | 0.261 | 0.598 | 0.593 | Yes |
| Breast cancer | rs2981582 | A | n/a | 0.000178 | 218 | 2382 | 10 | FGFR2 | 0.364 | 0.163 | 0.562 | 0.231 | Yes |
| Breast cancer | rs3803662 | A | n/a | 1.44E-05 | 218 | 2382 | 16 | TOX3 | 0.437 | 0.236 | 0.635 | 0.182 | Yes |
| Breast cancer | rs13387042 | A | n/a | 2.00E-05 | 218 | 2382 | 2 | Intergenic | 0.415 | 0.211 | 0.616 | 0.223 | Yes |
| Breast cancer | rs3817198 | C | n/a | 0.075 | 218 | 2382 | 11 | LSP1 | 0.149 | -0.058 | 0.357 | 0.068 | No |
| Breast cancer | rs11249433 | G | n/a | 0.223 | 218 | 2382 | 1 | Intergenic | 0.076 | -0.128 | 0.278 | 0.148 | No |
| Breast cancer | rs889312 | A | n/a | 0.947 | 218 | 2382 | 5 | MAP3K1 | -0.176 | -0.385 | 0.041 | 0.122 | No |
| Breast cancer | rs13281615 | A | n/a | 0.783 | 218 | 2382 | 8 | Intergenic | -0.079 | -0.278 | 0.117 | 0.077 | No |
| Celiac disease | rs917997 | T | n/a | 0.0445 | 23 | 4819 | 2 | IL18RAP | 0.532 | -0.073 | 1.141 | 0.174 | Yes |
| Celiac disease | rs13098911 | T | n/a | 0.0187 | 23 | 4819 | 3 | CCR1 | 0.794 | 0.030 | 1.560 | 0.262 | Yes |
| Celiac disease | rs2187668 | T | n/a | 5.59E-06 | 23 | 4819 | 6 | HLA-DQA1 | 1.301 | 0.683 | 1.920 | 1.829 | Yes |
| Celiac disease | rs802734 | G | n/a | 0.00746 | 23 | 4819 | 6 | PTPRK | 0.711 | 0.131 | 1.292 | 0.157 | Yes |
| Celiac disease | rs1250552 | A | n/a | 0.877 | 23 | 4819 | 10 | ZMIZ1 | -0.347 | -0.928 | 0.236 | 0.113 | No |
| Celiac disease | rs11221332 | T | n/a | 0.391 | 23 | 4819 | 11 | ETS1 | 0.096 | -0.580 | 0.775 | 0.191 | No |
| Celiac disease | rs653178 | C | n/a | 0.647 | 23 | 4819 | 12 | SH2B3 | -0.111 | -0.693 | 0.462 | 0.182 | No |
| Celiac disease | rs12928822 | C | n/a | 0.597 | 23 | 4819 | 16 | CIITA | -0.096 | -0.859 | 0.673 | 0.148 | No |
| Celiac disease | rs1893217 | G | n/a | 0.957 | 23 | 4819 | 18 | PTPN2 | -0.985 | -2.120 | 0.191 | 0.157 | No |
| Celiac disease | rs6691768 | A | n/a | 0.402 | 23 | 4819 | 1 | NFIA | 0.075 | -0.519 | 0.673 | 0.104 | No |
| Celiac disease | rs10903122 | G | n/a | 0.627 | 23 | 4819 | 1 | RUNX3 | -0.098 | -0.673 | 0.482 | 0.113 | No |
| Celiac disease | rs12727642 | A | n/a | 0.524 | 23 | 4819 | 1 | PARK7 | -0.024 | -0.833 | 0.777 | 0.131 | No |
| Celiac disease | rs3748816 | A | n/a | 0.327 | 23 | 4819 | 1 | TNFRSF14 | 0.145 | -0.482 | 0.777 | 0.113 | No |
| Celiac disease | rs2816316 | A | n/a | 0.449 | 23 | 4819 | 1 | RGS1 | 0.050 | -0.713 | 0.821 | 0.223 | No |
| Celiac disease | rs296547 | C | n/a | 0.765 | 23 | 4819 | 1 | Intergenic | -0.213 | -0.802 | 0.371 | 0.113 | No |
| Celiac disease | rs4819388 | C | n/a | 0.959 | 23 | 4819 | 21 | ICOSLG | -0.515 | -1.105 | 0.073 | 0.131 | No |
| Celiac disease | rs17035378 | T | n/a | 0.0715 | 23 | 4819 | 2 | PLEK | 0.544 | -0.186 | 1.273 | 0.131 | No |
| Celiac disease | rs13003464 | G | n/a | 0.139 | 23 | 4819 | 2 | REL | 0.317 | -0.261 | 0.896 | 0.140 | No |
| Celiac disease | rs13010713 | G | n/a | 0.161 | 23 | 4819 | 2 | ITGA4 | 0.294 | -0.288 | 0.875 | 0.122 | No |
| Celiac disease | rs4675374 | T | n/a | 0.191 | 23 | 4819 | 2 | CTLA4 | 0.290 | -0.357 | 0.932 | 0.131 | No |
| Celiac disease | rs13314993 | G | n/a | 0.653 | 23 | 4819 | 3 | CCR4 | -0.118 | -0.703 | 0.462 | 0.122 | No |
| Celiac disease | rs11712165 | G | n/a | 0.189 | 23 | 4819 | 3 | CD80 | 0.262 | -0.322 | 0.844 | 0.122 | No |
| Celiac disease | rs17810546 | G | n/a | 0.344 | 23 | 4819 | 3 | IL12A | 0.177 | -0.693 | 1.037 | 0.307 | No |
| Celiac disease | rs1464510 | A | rs13076312 | 0.444 | 23 | 4819 | 3 | LPP | 0.042 | -0.545 | 0.626 | 0.255 | No |
| Celiac disease | rs6822844 | G | n/a | 0.945 | 23 | 4819 | 4 | KIAA1109 | -0.550 | -1.230 | 0.128 | 0.365 | No |
| Celiac disease | rs1033180 | T | n/a | 0.314 | 23 | 4819 | 6 | IRF4 | 0.253 | -0.777 | 1.281 | 0.191 | No |
| Celiac disease | rs10806425 | A | n/a | 0.217 | 23 | 4819 | 6 | BACH2 | 0.232 | -0.351 | 0.821 | 0.122 | No |
| Celiac disease | rs2327832 | G | n/a | 0.679 | 23 | 4819 | 6 | LOC391040 | -0.182 | -0.942 | 0.582 | 0.207 | No |
| Celiac disease | rs1738074 | T | n/a | 0.318 | 23 | 4819 | 6 | TAGAP | 0.140 | -0.446 | 0.723 | 0.148 | No |
| Celiac disease | rs9792269 | A | n/a | 0.835 | 23 | 4819 | 8 | Intergenic | -0.312 | -0.940 | 0.315 | 0.131 | No |
| Celiac disease | rs5979785 | T | n/a | 0.698 | 23 | 4819 | X | TLR7 | -0.209 | -0.844 | 0.419 | 0.131 | No |
| Colorectal cancer | rs10795668 | A | n/a | 0.768 | 77 | 4640 | 10 | Intergenic | -0.131 | -0.482 | 0.223 | 0.113 | No |
| Colorectal cancer | rs3802842 | C | n/a | 0.109 | 77 | 4640 | 11 | Intergenic | 0.211 | -0.128 | 0.548 | 0.104 | No |
| Colorectal cancer | rs4444235 | C | n/a | 0.354 | 77 | 4640 | 14 | BMP4 | 0.061 | -0.255 | 0.386 | 0.104 | No |
| Colorectal cancer | rs9929218 | A | n/a | 0.3 | 77 | 4640 | 16 | CDH1 | 0.091 | -0.247 | 0.431 | 0.095 | No |
| Colorectal cancer | rs4939827 | T | n/a | 0.538 | 77 | 4640 | 18 | SMAD7 | -0.016 | -0.329 | 0.300 | 0.182 | No |
| Colorectal cancer | rs10411210 | C | n/a | 0.416 | 77 | 4640 | 19 | RHPN2 | 0.056 | -0.464 | 0.580 | 0.140 | No |
| Colorectal cancer | rs961253 | A | n/a | 0.759 | 77 | 4640 | 20 | Intergenic | -0.122 | -0.464 | 0.223 | 0.113 | No |
| Colorectal cancer | rs16892766 | A | n/a | 0.226 | 77 | 4640 | 8 | EIF3H | 0.234 | -0.385 | 0.844 | 0.239 | No |
| Colorectal cancer | rs6983267 | G | n/a | 0.183 | 77 | 4640 | 8 | Intergenic | 0.149 | -0.174 | 0.462 | 0.215 | No |
| COPD | rs13180 | T | n/a | 0.26 | 403 | 2306 | 15 | CHRNA3 | 0.051 | -0.105 | 0.207 | 0.262 | No |
| COPD | rs7671167 | T | n/a | 0.0968 | 403 | 2306 | 4 | FAM13A | 0.101 | -0.051 | 0.247 | 0.278 | No |
| COPD | rs1828591 | A | n/a | 0.489 | 403 | 2306 | 4 | HHIP | 0.002 | -0.148 | 0.151 | 0.322 | No |
| Coronary artery disease | rs1333049 | C | n/a | 0.0214 | 498 | 1275 | 9 | Intergenic | 0.150 | 0.000 | 0.301 | 0.307 | Yes |
| Coronary artery disease | rs501120 | T | n/a | 0.206 | 498 | 1275 | 10 | CXCL12 | 0.091 | -0.128 | 0.307 | 0.285 | No |
| Coronary artery disease | rs599839 | A | n/a | 0.654 | 498 | 1275 | 1 | PSRC1 | -0.034 | -0.207 | 0.139 | 0.255 | No |
| Coronary artery disease | rs9818870 | T | rs2306374 | 0.401 | 498 | 1275 | 3 | MRAS | 0.026 | -0.174 | 0.223 | 0.140 | No |
| Coronary artery disease | rs6922269 | A | n/a | 0.331 | 498 | 1275 | 6 | MTHFD1L | 0.037 | -0.131 | 0.211 | 0.207 | No |
| Crohn's disease | rs224136 | C | n/a | 0.043 | 84 | 13288 | 10 | Intergenic | 0.429 | -0.058 | 0.916 | 0.513 | Yes |
| Crohn's disease | rs11190140 | T | n/a | 0.0112 | 84 | 13288 | 10 | NKX2-3 | 0.354 | 0.049 | 0.663 | 0.182 | Yes |
| Crohn's disease | rs11805303 | T | n/a | 0.0139 | 84 | 13288 | 1 | IL23R | 0.351 | 0.039 | 0.663 | 0.329 | Yes |
| Crohn's disease | rs11465804 | T | n/a | 0.0299 | 84 | 13288 | 1 | IL23R | 0.829 | -0.062 | 1.721 | 0.916 | Yes |
| Crohn's disease | rs762421 | G | n/a | 0.0379 | 84 | 13288 | 21 | ICOSLG | 0.276 | -0.030 | 0.582 | 0.122 | Yes |
| Crohn's disease | rs3197999 | A | n/a | 0.0347 | 84 | 13288 | 3 | MST1 | 0.290 | -0.020 | 0.598 | 0.182 | Yes |
| Crohn's disease | rs4613763 | C | n/a | 0.0453 | 84 | 13288 | 5 | PTGER4 | 0.345 | -0.058 | 0.755 | 0.278 | Yes |
| Crohn's disease | rs10758669 | C | n/a | 0.000175 | 84 | 13288 | 9 | JAK2 | 0.547 | 0.247 | 0.850 | 0.113 | Yes |
| Crohn's disease | rs17582416 | G | rs7923172 | 0.703 | 84 | 13288 | 10 | Intergenic | -0.088 | -0.412 | 0.236 | 0.148 | No |
| Crohn's disease | rs10995271 | C | n/a | 0.437 | 84 | 13288 | 10 | ZNF365 | 0.025 | -0.285 | 0.342 | 0.223 | No |
| Crohn's disease | rs7927894 | T | n/a | 0.0843 | 84 | 13288 | 11 | C11orf30 | 0.216 | -0.094 | 0.525 | 0.148 | No |
| Crohn's disease | rs11175593 | T | n/a | 0.0644 | 84 | 13288 | 12 | LRRK2 | 0.585 | -0.174 | 1.345 | 0.432 | No |
| Crohn's disease | rs3764147 | G | n/a | 0.0621 | 84 | 13288 | 13 | C13orf31 | 0.261 | -0.073 | 0.593 | 0.223 | No |
| Crohn's disease | rs2066847 | C | n/a | 0.151 | 84 | 13288 | 16 | NOD2 | 0.430 | -0.386 | 1.250 | 1.384 | No |
| Crohn's disease | rs2872507 | A | n/a | 0.923 | 84 | 13288 | 17 | ORMDL3 | -0.224 | -0.536 | 0.083 | 0.113 | No |
| Crohn's disease | rs744166 | A | n/a | 0.528 | 84 | 13288 | 17 | STAT3 | -0.011 | -0.322 | 0.301 | 0.166 | No |
| Crohn's disease | rs2542151 | G | n/a | 0.246 | 84 | 13288 | 18 | PTPN2 | 0.143 | -0.262 | 0.545 | 0.300 | No |
| Crohn's disease | rs2476601 | G | n/a | 0.843 | 84 | 13288 | 1 | PTPN22 | -0.246 | -0.734 | 0.231 | 0.270 | No |
| Crohn's disease | rs2274910 | C | n/a | 0.184 | 84 | 13288 | 1 | ITLN1 | 0.153 | -0.182 | 0.494 | 0.131 | No |
| Crohn's disease | rs9286879 | G | n/a | 0.786 | 84 | 13288 | 1 | Intergenic | -0.148 | -0.511 | 0.215 | 0.174 | No |
| Crohn's disease | rs10801047 | A | n/a | 0.887 | 84 | 13288 | 1 | Intergenic | -0.435 | -1.147 | 0.274 | 0.385 | No |
| Crohn's disease | rs11584383 | T | n/a | 0.545 | 84 | 13288 | 1 | Intergenic | -0.020 | -0.357 | 0.315 | 0.166 | No |
| Crohn's disease | rs1736135 | T | rs1736148 | 0.687 | 84 | 13288 | 21 | Intergenic | -0.076 | -0.386 | 0.231 | 0.166 | No |
| Crohn's disease | rs3828309 | G | rs2289472 | 0.169 | 84 | 13288 | 2 | ATG16L1 | 0.149 | -0.151 | 0.457 | 0.223 | No |
| Crohn's disease | rs9292777 | C | n/a | 0.495 | 84 | 13288 | 5 | Intergenic | 0.002 | -0.307 | 0.315 | 0.293 | No |
| Crohn's disease | rs2188962 | T | n/a | 0.154 | 84 | 13288 | 5 | Intergenic | 0.159 | -0.151 | 0.464 | 0.223 | No |
| Crohn's disease | rs11747270 | G | n/a | 0.172 | 84 | 13288 | 5 | IRGM | 0.232 | -0.248 | 0.713 | 0.285 | No |
| Crohn's disease | rs10045431 | C | n/a | 0.132 | 84 | 13288 | 5 | IL12B | 0.197 | -0.151 | 0.542 | 0.104 | No |
| Crohn's disease | rs6908425 | C | n/a | 0.422 | 84 | 13288 | 6 | CDKAL1 | 0.038 | -0.336 | 0.416 | 0.191 | No |
| Crohn's disease | rs7746082 | C | rs6938089 | 0.605 | 84 | 13288 | 6 | PRDM1 | -0.045 | -0.372 | 0.288 | 0.157 | No |
| Crohn's disease | rs2301436 | T | n/a | 0.751 | 84 | 13288 | 6 | CCR6 | -0.106 | -0.416 | 0.199 | 0.191 | No |
| Crohn's disease | rs1456893 | A | n/a | 0.541 | 84 | 13288 | 7 | Intergenic | -0.017 | -0.344 | 0.315 | 0.182 | No |
| Crohn's disease | rs1551398 | A | n/a | 0.96 | 84 | 13288 | 8 | Intergenic | -0.273 | -0.577 | 0.030 | 0.077 | No |
| Crohn's disease | rs4263839 | G | n/a | 0.236 | 84 | 13288 | 9 | TNFSF15 | 0.123 | -0.211 | 0.464 | 0.199 | No |
| Early onset heart attack | rs1746048 | C | n/a | 0.328 | 71 | 4389 | 10 | CXCL12 | 0.115 | -0.385 | 0.616 | 0.157 | No |
| Early onset heart attack | rs1122608 | G | rs8102273 | 0.777 | 71 | 4389 | 19 | LDLR | -0.138 | -0.494 | 0.215 | 0.140 | No |
| Early onset heart attack | rs646776 | T | n/a | 0.575 | 71 | 4389 | 1 | CELSR2 | -0.038 | -0.431 | 0.358 | 0.174 | No |
| Early onset heart attack | rs11206510 | T | n/a | 0.434 | 71 | 4389 | 1 | PCSK9 | 0.036 | -0.386 | 0.457 | 0.140 | No |
| Early onset heart attack | rs17465637 | C | n/a | 0.65 | 71 | 4389 | 1 | MIA3 | -0.073 | -0.446 | 0.293 | 0.131 | No |
| Early onset heart attack | rs9982601 | T | rs973754 | 0.524 | 71 | 4389 | 21 | SLC5A3 | -0.015 | -0.494 | 0.470 | 0.182 | No |
| Early onset heart attack | rs6725887 | C | n/a | 0.645 | 71 | 4389 | 2 | WDR12 | -0.100 | -0.626 | 0.431 | 0.157 | No |
| Early onset heart attack | rs12526453 | C | rs4714990 | 0.061 | 71 | 4389 | 6 | PHACTR1 | 0.278 | -0.073 | 0.631 | 0.113 | No |
| Early onset heart attack | rs4977574 | G | n/a | 0.34 | 71 | 4389 | 9 | CDKN2A | 0.070 | -0.261 | 0.399 | 0.255 | No |
| Early onset prostate cancer | rs2735839 | G | n/a | 0.0122 | 88 | 1064 | 19 | KLK3 | 0.604 | 0.077 | 1.131 | 0.182 | Yes |
| Early onset prostate cancer | rs9364554 | T | n/a | 0.00775 | 88 | 1064 | 6 | SLC22A3 | 0.412 | 0.086 | 0.737 | 0.157 | Yes |
| Early onset prostate cancer | rs1016343 | T | n/a | 0.0438 | 88 | 1064 | 8 | Intergenic | 0.316 | -0.041 | 0.678 | 0.315 | Yes |
| Early onset prostate cancer | rs721048 | A | rs2710646 | 0.382 | 88 | 1064 | 2 | EHBP1 | 0.062 | -0.336 | 0.462 | 0.140 | No |
| Early onset prostate cancer | rs2660753 | T | n/a | 0.0727 | 88 | 1064 | 3 | SLC22A3 | 0.320 | -0.117 | 0.756 | 0.166 | No |
| Early onset prostate cancer | rs6465657 | C | n/a | 0.315 | 88 | 1064 | 7 | LMTK2 | 0.077 | -0.231 | 0.386 | 0.113 | No |
| Early onset prostate cancer | rs5945572 | A | n/a | 0.541 | 88 | 1064 | X | NUDT10 | -0.024 | -0.351 | 0.301 | 0.207 | No |
| Eye color (blue/brown) | rs1667394 | T | n/a | 0.00E-01 | 1678 | 1170 | 15 | OCA2 | 3.869 | 3.586 | 4.153 | 3.382 | Yes |
| Eye color (blue/green) | rs1393350 | A | n/a | 4.28E-25 | 2125 | 1663 | 11 | TYR | 0.531 | 0.432 | 0.631 | 0.419 | Yes |
| Eye color (blue/green) | rs12896399 | T | n/a | 4.18E-34 | 2125 | 1663 | 14 | SLC24A4 | 0.569 | 0.478 | 0.654 | 0.723 | Yes |
| Eye color (blue/green) | rs1667394 | T | n/a | 6.16E-37 | 2125 | 1663 | 15 | OCA2 | 1.715 | 1.427 | 2.040 | 1.908 | Yes |
| Eye color (blue/green) | rs1408799 | C | rs927869 | 1.71E-07 | 2125 | 1663 | 9 | TYRP1 | 0.245 | 0.151 | 0.342 | 0.336 | Yes |
| Freckling | rs1042602 | C | n/a | 9.69E-09 | 2182 | 4136 | 11 | TYR | 0.221 | 0.148 | 0.300 | 0.278 | Yes |
| Freckling | rs1805007 | T | n/a | 3.76E-62 | 2182 | 4136 | 16 | MC1R | 1.113 | 0.978 | 1.247 | 1.475 | Yes |
| Freckling | rs1540771 | T | n/a | 3.93E-28 | 2182 | 4136 | 6 | SEC5L1 | 0.409 | 0.336 | 0.482 | 0.336 | Yes |
| Haircolor (blond/brown) | rs12821256 | C | n/a | 5.48E-09 | 1700 | 2058 | 12 | KITLG | 0.435 | 0.288 | 0.580 | 0.842 | Yes |
| Haircolor (blond/brown) | rs12896399 | T | n/a | 5.69E-19 | 1700 | 2058 | 14 | SLC24A4 | 0.420 | 0.329 | 0.513 | 0.940 | Yes |
| Haircolor (blond/brown) | rs1667394 | T | n/a | 1.43E-65 | 1700 | 2058 | 15 | OCA2 | 1.120 | 0.990 | 1.250 | 1.597 | Yes |
| Haircolor (blond/brown) | rs1805007 | T | n/a | 9.42E-15 | 1700 | 2058 | 16 | MC1R | 0.725 | 0.531 | 0.916 | 0.850 | Yes |
| Haircolor (red) | rs1805007 | T | n/a | 1.28E-30 | 1745 | 4556 | 16 | MC1R | 0.779 | 0.647 | 0.912 | 2.523 | Yes |
| IBD | rs7517847 | G | n/a | 0.954 | 250 | 12808 | 1 | IL23R | -0.156 | -0.336 | 0.030 | 0.476 | No |
| Juvenile allergic asthma | rs7216389 | T | n/a | 0.00572 | 641 | 6584 | 17 | ORMDL3 | 0.148 | 0.030 | 0.262 | 0.372 | Yes |
| Juvenile allergic asthma | rs2786098 | G | n/a | 0.181 | 641 | 6584 | 1 | DENND1B | 0.067 | -0.077 | 0.211 | 0.358 | No |
| Juvenile allergic asthma | rs1588265 | G | n/a | 0.556 | 641 | 6584 | 5 | PDE4D | -0.009 | -0.139 | 0.113 | 0.166 | No |
| Juvenile autism | rs4307059 | T | n/a | 0.351 | 24 | 11096 | 5 | CDH10 | 0.116 | -0.478 | 0.708 | 0.174 | No |
| Kidney stones | rs219780 | C | rs219784 | 0.0151 | 1154 | 12110 | 21 | CLDN14 | 0.116 | 0.010 | 0.223 | 0.223 | Yes |
| Lung cancer | rs8034191 | C | n/a | 0.279 | 19 | 10902 | 15 | CHRNA3 | 0.196 | -0.457 | 0.844 | 0.255 | No |
| Lung cancer | rs401681 | C | n/a | 0.557 | 19 | 10902 | 5 | CLPTM1L | -0.047 | -0.688 | 0.598 | 0.140 | No |
| Lung cancer | rs3117582 | G | rs3132450 | 0.267 | 19 | 10902 | 6 | BAT3 | 0.299 | -0.635 | 1.241 | 0.215 | No |
| Lupus | rs4963128 | C | n/a | 0.296 | 52 | 11675 | 11 | KIAA1542 | 0.114 | -0.300 | 0.528 | 0.247 | No |
| Lupus | rs9888739 | T | n/a | 0.102 | 52 | 11675 | 16 | ITGAM | 0.331 | -0.174 | 0.842 | 0.482 | No |
| Lupus | rs10798269 | G | n/a | 0.763 | 52 | 11675 | 1 | Intergenic | -0.145 | -0.545 | 0.255 | 0.199 | No |
| Lupus | rs3821236 | A | n/a | 0.288 | 52 | 11675 | 2 | STAT4 | 0.133 | -0.329 | 0.598 | 0.399 | No |
| Lupus | rs6445975 | G | n/a | 0.169 | 52 | 11675 | 3 | PXK | 0.200 | -0.207 | 0.616 | 0.223 | No |
| Lupus | rs10516487 | G | n/a | 0.618 | 52 | 11675 | 4 | BANK1 | -0.064 | -0.478 | 0.351 | 0.322 | No |
| Lupus | rs3131379 | A | n/a | 0.133 | 52 | 11675 | 6 | HLA | 0.322 | -0.247 | 0.892 | 0.859 | No |
| Lupus | rs12537284 | A | n/a | 0.206 | 52 | 11675 | 7 | IRF5 | 0.218 | -0.300 | 0.734 | 0.432 | No |
| Lupus | rs13277113 | A | n/a | 0.329 | 52 | 11675 | 8 | C8orf13 | 0.100 | -0.344 | 0.545 | 0.329 | No |
| Male pattern baldness | rs1160312 | A | rs201594 | 2.53E-10 | 3786 | 746 | 20 | PAX1 | 0.353 | 0.239 | 0.464 | 0.470 | Yes |
| Male pattern baldness | rs6625163 | A | n/a | 1.34E-42 | 3786 | 746 | X | AR | 1.219 | 1.079 | 1.347 | 1.194 | Yes |
| Melanoma | rs1393350 | A | n/a | 7.12E-05 | 225 | 10992 | 11 | TYR | 0.381 | 0.186 | 0.580 | 0.255 | Yes |
| Melanoma | rs4785763 | A | n/a | 0.00731 | 225 | 10992 | 16 | MC1R | 0.238 | 0.041 | 0.431 | 0.307 | Yes |
| Melanoma | rs258322 | A | n/a | 0.0185 | 225 | 10992 | 16 | MC1R | 0.306 | 0.020 | 0.598 | 0.513 | Yes |
| Melanoma | rs910873 | A | rs4911442 | 0.00156 | 225 | 10992 | 20 | CDC91L1 | 0.387 | 0.131 | 0.642 | 0.560 | Yes |
| Melanoma | rs2284063 | A | n/a | 0.334 | 225 | 10992 | 22 | Intergenic | 0.043 | -0.148 | 0.236 | 0.182 | No |
| Multiple sclerosis | rs2104286 | T | n/a | 0.00721 | 72 | 13287 | 10 | IL2RA | 0.536 | 0.104 | 0.967 | 0.140 | Yes |
| Multiple sclerosis | rs703842 | A | n/a | 0.00793 | 72 | 13287 | 12 | METTL1 | 0.480 | 0.083 | 0.868 | 0.207 | Yes |
| Multiple sclerosis | rs744166 | G | n/a | 0.017 | 72 | 13287 | 17 | STAT3 | 0.352 | 0.020 | 0.678 | 0.140 | Yes |
| Multiple sclerosis | rs3135388 | A | n/a | 1.09E-05 | 72 | 13287 | 6 | HLA | 0.799 | 0.416 | 1.171 | 1.012 | Yes |
| Multiple sclerosis | rs17824933 | G | rs929230 | 0.122 | 72 | 13287 | 11 | CD6 | 0.221 | -0.140 | 0.580 | 0.166 | No |
| Multiple sclerosis | rs1800693 | C | n/a | 0.311 | 72 | 13287 | 12 | TNFRSF1A | 0.084 | -0.247 | 0.416 | 0.182 | No |
| Multiple sclerosis | rs17445836 | G | rs11642873 | 0.453 | 72 | 13287 | 16 | IRF8 | 0.025 | -0.392 | 0.446 | 0.223 | No |
| Multiple sclerosis | rs2300747 | A | rs12044852 | 0.414 | 72 | 13287 | 1 | CD58 | 0.061 | -0.494 | 0.615 | 0.262 | No |
| Multiple sclerosis | rs10492972 | C | rs12136376 | 0.645 | 72 | 13287 | 1 | KIF1B | -0.070 | -0.431 | 0.293 | 0.293 | No |
| Multiple sclerosis | rs6074022 | C | n/a | 0.195 | 72 | 13287 | 20 | CD40 | 0.159 | -0.207 | 0.528 | 0.182 | No |
| Multiple sclerosis | rs882300 | C | n/a | 0.0922 | 72 | 13287 | 2 | CXCR4 | 0.236 | -0.104 | 0.580 | 0.174 | No |
| Multiple sclerosis | rs2523393 | A | rs2523395 | 0.998 | 72 | 13287 | 6 | HLA | -0.513 | -0.863 | -0.166 | 0.247 | No |
| Osteoarthritis | rs3815148 | C | n/a | 0.664 | 1685 | 659 | 7 | 7q22.3 | -0.033 | -0.186 | 0.122 | 0.131 | No |
| Ovarian cancer | rs3814113 | T | n/a | 0.0189 | 16 | 4935 | 9 | BNC2 | 0.970 | 0.010 | 1.926 | 0.199 | Yes |
| Pancreatic cancer | rs9543325 | C | n/a | 0.639 | 9 | 10911 | 13 | KLF5 | -0.179 | -1.160 | 0.799 | 0.231 | No |
| Pancreatic cancer | rs3790844 | A | n/a | 0.114 | 9 | 10911 | 1 | NR5A2 | 0.878 | -0.593 | 2.303 | 0.262 | No |
| Pancreatic cancer | rs505922 | C | n/a | 0.866 | 9 | 10911 | 9 | ABO | -0.621 | -1.733 | 0.494 | 0.182 | No |
| Parkinson's disease | rs199533 | G | n/a | 2.46E-10 | 2274 | 5336 | 17 | NSF | 0.291 | 0.199 | 0.385 | 0.247 | Yes |
| Parkinson's disease | rs393152 | A | n/a | 9.88E-10 | 2274 | 5336 | 17 | MAPT | 0.271 | 0.186 | 0.357 | 0.262 | Yes |
| Parkinson's disease | rs2736990 | G | n/a | 2.15E-12 | 2274 | 5336 | 4 | SNCA | 0.245 | 0.174 | 0.315 | 0.207 | Yes |
| Parkinson's disease | rs6532197 | G | n/a | 0.00392 | 2274 | 5336 | 4 | MMRN1 | 0.169 | 0.049 | 0.293 | 0.278 | Yes |
| Parkinson's disease | rs17115100 | G | n/a | 0.555 | 2274 | 5336 | 10 | CYP17A1 | -0.008 | -0.122 | 0.105 | 0.223 | No |
| Parkinson's disease | rs823128 | A | n/a | 0.0531 | 2274 | 5336 | 1 | PARK16 | 0.155 | -0.030 | 0.342 | 0.419 | No |
| Juvenile IBD | rs1250550 | C | n/a | 0.0798 | 25 | 12816 | 10 | ZMIZ1 | 0.466 | -0.186 | 1.115 | 0.148 | No |
| Juvenile IBD | rs8049439 | C | n/a | 0.612 | 25 | 12816 | 16 | IL27 | -0.083 | -0.663 | 0.494 | 0.131 | No |
| Juvenile IBD | rs5743289 | T | n/a | 0.19 | 25 | 12816 | 16 | NOD2 | 0.301 | -0.371 | 0.971 | 0.378 | No |
| Juvenile IBD | rs10500264 | A | n/a | 0.198 | 25 | 12816 | 19 | Intergenic | 0.290 | -0.378 | 0.968 | 0.191 | No |
| Juvenile IBD | rs11209026 | G | n/a | 0.411 | 25 | 12816 | 1 | IL23R | 0.134 | -1.022 | 1.303 | 0.940 | No |
| Juvenile IBD | rs2315008 | G | n/a | 0.316 | 25 | 12816 | 20 | TNFRSF6B | 0.152 | -0.464 | 0.777 | 0.307 | No |
| Juvenile IBD | rs2836878 | G | n/a | 0.675 | 25 | 12816 | 21 | PSMG1 | -0.140 | -0.755 | 0.464 | 0.344 | No |
| Juvenile IBD | rs2412973 | A | n/a | 0.722 | 25 | 12816 | 22 | HORMAD2 | -0.169 | -0.732 | 0.400 | 0.140 | No |
| Juvenile IBD | rs6478109 | G | n/a | 0.427 | 25 | 12816 | 9 | TNFSF15 | 0.057 | -0.545 | 0.663 | 0.307 | No |
| Prostate cancer | rs10993994 | T | n/a | 0.00805 | 249 | 1062 | 10 | MSMB | 0.246 | 0.049 | 0.445 | 0.148 | Yes |
| Prostate cancer | rs7127900 | A | n/a | 0.00552 | 249 | 1062 | 11 | IGF2 | 0.300 | 0.073 | 0.528 | 0.199 | Yes |
| Prostate cancer | rs11228565 | A | rs7130881 | 0.00364 | 249 | 1062 | 11 | Intergenic | 0.341 | 0.095 | 0.582 | 0.207 | Yes |
| Prostate cancer | rs4430796 | A | n/a | 0.00196 | 249 | 1062 | 17 | HNF1B | 0.289 | 0.094 | 0.478 | 0.166 | Yes |
| Prostate cancer | rs1859962 | G | n/a | 0.0025 | 249 | 1062 | 17 | Intergenic | 0.279 | 0.083 | 0.478 | 0.182 | Yes |
| Prostate cancer | rs17021918 | C | n/a | 0.00628 | 249 | 1062 | 4 | PDLIM5 | 0.267 | 0.051 | 0.478 | 0.104 | Yes |
| Prostate cancer | rs4242382 | A | n/a | 0.0394 | 249 | 1062 | 8 | Intergenic | 0.289 | -0.039 | 0.616 | 0.507 | Yes |
| Prostate cancer | rs1447295 | A | n/a | 0.0278 | 249 | 1062 | 8 | Intergenic | 0.314 | -0.010 | 0.635 | 0.457 | Yes |
| Prostate cancer | rs16901979 | A | n/a | 5.14E-05 | 249 | 1062 | 8 | Intergenic | 0.858 | 0.416 | 1.309 | 0.588 | Yes |
| Prostate cancer | rs8102476 | C | n/a | 0.0963 | 249 | 1062 | 19 | Intergenic | 0.129 | -0.068 | 0.329 | 0.113 | No |
| Prostate cancer | rs5759167 | T | n/a | 0.624 | 249 | 1062 | 22 | NR | -0.031 | -0.223 | 0.166 | 0.148 | No |
| Prostate cancer | rs1465618 | T | n/a | 0.574 | 249 | 1062 | 2 | THADA | -0.024 | -0.274 | 0.223 | 0.077 | No |
| Prostate cancer | rs12621278 | A | n/a | 0.168 | 249 | 1062 | 2 | ITGA6 | 0.209 | -0.223 | 0.635 | 0.285 | No |
| Prostate cancer | rs10934853 | A | n/a | 0.214 | 249 | 1062 | 3 | Intergenic | 0.089 | -0.131 | 0.301 | 0.113 | No |
| Prostate cancer | rs7679673 | A | rs1391438 | 0.964 | 249 | 1062 | 4 | TET2 | -0.191 | -0.400 | 0.020 | 0.095 | No |
| Prostate cancer | rs1512268 | T | n/a | 0.718 | 249 | 1062 | 8 | NKX3.1 | -0.058 | -0.261 | 0.140 | 0.166 | No |
| Prostate cancer | rs6983267 | G | n/a | 0.164 | 249 | 1062 | 8 | Intergenic | 0.098 | -0.095 | 0.288 | 0.247 | No |
| Prostate cancer | rs445114 | T | rs587948 | 0.1 | 249 | 1062 | 8 | Intergenic | 0.132 | -0.073 | 0.336 | 0.131 | No |
| Psoriasis | rs2201841 | G | n/a | 0.0451 | 833 | 4291 | 1 | IL23R | 0.098 | -0.010 | 0.207 | 0.122 | Yes |
| Psoriasis | rs17728338 | A | rs1024995 | 0.0303 | 833 | 4291 | 5 | TNIP1 | 0.151 | -0.010 | 0.315 | 0.464 | Yes |
| Psoriasis | rs2082412 | G | rs3213094 | 0.00966 | 833 | 4291 | 5 | IL12B | 0.163 | 0.020 | 0.301 | 0.365 | Yes |
| Psoriasis | rs12191877 | T | rs10484554 | 2.10E-17 | 833 | 4291 | 6 | HLA | 0.557 | 0.425 | 0.688 | 0.971 | Yes |
| Psoriasis | rs610604 | G | n/a | 0.036 | 833 | 4291 | 6 | TNFAIP3 | 0.101 | -0.010 | 0.211 | 0.174 | Yes |
| Psoriasis | rs2066808 | A | n/a | 0.0803 | 833 | 4291 | 12 | IL23A | 0.157 | -0.068 | 0.371 | 0.293 | No |
| Psoriasis | rs20541 | G | n/a | 0.0953 | 833 | 4291 | 5 | IL13 | 0.090 | -0.041 | 0.223 | 0.239 | No |
| Rheumatoid arthritis | rs6679677 | A | n/a | 0.000148 | 308 | 12845 | 1 | PTPN11 | 0.440 | 0.198 | 0.673 | 0.582 | Yes |
| Rheumatoid arthritis | rs4810485 | G | rs6074022 | 0.0372 | 308 | 12845 | 20 | CD40 | 0.175 | -0.020 | 0.365 | 0.140 | Yes |
| Rheumatoid arthritis | rs1678542 | C | rs11172254 | 0.171 | 308 | 12845 | 12 | KIF5A | 0.081 | -0.086 | 0.248 | 0.113 | No |
| Rheumatoid arthritis | rs3890745 | T | n/a | 0.236 | 308 | 12845 | 1 | MMEL1 | 0.064 | -0.105 | 0.239 | 0.113 | No |
| Rheumatoid arthritis | rs13017599 | A | n/a | 0.38 | 308 | 12845 | 2 | REL | 0.026 | -0.140 | 0.198 | 0.191 | No |
| Rheumatoid arthritis | rs231735 | T | n/a | 0.202 | 308 | 12845 | 2 | CTLA4 | 0.068 | -0.094 | 0.231 | 0.157 | No |
| Rheumatoid arthritis | rs10499194 | C | n/a | 0.797 | 308 | 12845 | 6 | TNFAIP3 | -0.076 | -0.255 | 0.105 | 0.285 | No |
| Rheumatoid arthritis | rs6920220 | A | n/a | 0.92 | 308 | 12845 | 6 | OLIG3 | -0.151 | -0.365 | 0.062 | 0.215 | No |
| Rheumatoid arthritis | rs2736340 | T | n/a | 0.534 | 308 | 12845 | 8 | BLK | -0.008 | -0.198 | 0.174 | 0.174 | No |
| Rheumatoid arthritis | rs2812378 | G | rs11574914 | 0.521 | 308 | 12845 | 9 | CCL21 | -0.005 | -0.174 | 0.163 | 0.113 | No |
| Rheumatoid arthritis | rs3761847 | G | n/a | 0.437 | 308 | 12845 | 9 | TRAF1-C5 | 0.013 | -0.151 | 0.174 | 0.278 | No |
| Schizophrenia | rs12807809 | T | n/a | 0.157 | 23 | 11166 | 11 | NRGN | 0.476 | -0.446 | 1.404 | 0.140 | No |
| Schizophrenia | rs9960767 | C | n/a | 0.0957 | 23 | 11166 | 18 | TCF4 | 0.612 | -0.315 | 1.541 | 0.207 | No |
| Schizophrenia | rs6782299 | T | rs9838229 | 0.574 | 23 | 11166 | 3 | FXR1 | -0.061 | -0.703 | 0.580 | 0.095 | No |
| Schizophrenia | rs3131296 | C | n/a | 0.967 | 23 | 11166 | 6 | MHC | -0.646 | -1.348 | 0.051 | 0.174 | No |
| Schizophrenia | rs9272219 | G | n/a | 0.66 | 23 | 11166 | 6 | HLA | -0.132 | -0.761 | 0.494 | 0.131 | No |
| Schizophrenia | rs6932590 | T | n/a | 0.927 | 23 | 11166 | 6 | MHC | -0.461 | -1.079 | 0.166 | 0.148 | No |
| Schizophrenia | rs13194053 | T | rs6904071 | 0.995 | 23 | 11166 | 6 | MHC | -0.805 | -1.427 | -0.174 | 0.199 | No |
| Scleroderma | rs10488631 | C | n/a | 0.000462 | 17 | 10922 | 7 | TNPO | 1.177 | 0.431 | 1.897 | 0.405 | Yes |
| Scleroderma | rs2056626 | T | n/a | 0.372 | 17 | 10922 | 1 | CD247 | 0.115 | -0.580 | 0.806 | 0.148 | No |
| Scleroderma | rs3821236 | A | n/a | 0.63 | 17 | 10922 | 2 | STAT4 | -0.150 | -1.033 | 0.734 | 0.262 | No |
| Scleroderma | rs6457617 | T | n/a | 0.96 | 17 | 10922 | 6 | HLA | -0.622 | -1.309 | 0.086 | 0.315 | No |
| Stroke | rs12425791 | A | n/a | 0.973 | 146 | 10972 | 12 | NINJ2 | -0.310 | -0.631 | 0.010 | 0.255 | No |
| Stroke | rs2200733 | T | n/a | 0.638 | 146 | 10972 | 4 | NR | -0.064 | -0.416 | 0.285 | 0.231 | No |
| Testicular cancer | rs4624820 | A | n/a | 0.0156 | 17 | 6209 | 5 | SPRY4 | 0.824 | 0.062 | 1.609 | 0.315 | Yes |
| Testicular cancer | rs210138 | G | n/a | 0.000414 | 17 | 6209 | 6 | BAK1 | 1.108 | 0.425 | 1.792 | 0.405 | Yes |
| Testicular cancer | rs995030 | G | n/a | 0.287 | 17 | 6209 | 12 | KITLG | 0.271 | -0.673 | 1.221 | 0.936 | No |
| Thyroid cancer | rs944289 | T | n/a | 0.572 | 52 | 11234 | 14 | NKX2-1 | -0.036 | -0.431 | 0.351 | 0.315 | No |
| Thyroid cancer | rs965513 | A | n/a | 0.0559 | 52 | 11234 | 9 | FOXE1 | 0.317 | -0.077 | 0.713 | 0.560 | No |
| Type 1 diabetes | rs2292239 | T | n/a | 0.0179 | 54 | 11284 | 12 | ERBB3 | 0.407 | 0.030 | 0.788 | 0.247 | Yes |
| Type 1 diabetes | rs9272346 | G | rs9273363 | 2.22E-09 | 54 | 11284 | 6 | MHC | 1.084 | 0.713 | 1.470 | 1.703 | Yes |
| Type 1 diabetes | rs2647044 | A | rs2187668 | 3.32E-06 | 54 | 11284 | 6 | HLA | 0.960 | 0.525 | 1.391 | 2.116 | Yes |
| Type 1 diabetes | rs7020673 | G | rs7041847 | 0.0307 | 54 | 11284 | 9 | GLIS3 | 0.368 | -0.020 | 0.755 | 0.131 | Yes |
| Type 1 diabetes | rs947474 | G | rs10796045 | 0.854 | 54 | 11284 | 10 | PRKCQ | -0.287 | -0.820 | 0.248 | 0.095 | No |
| Type 1 diabetes | rs10509540 | C | rs1035796 | 0.188 | 54 | 11284 | 10 | C10orf59 | 0.175 | -0.211 | 0.560 | 0.285 | No |
| Type 1 diabetes | rs1004446 | G | n/a | 0.515 | 54 | 11284 | 11 | INS | -0.008 | -0.400 | 0.385 | 0.476 | No |
| Type 1 diabetes | rs4763879 | A | n/a | 0.198 | 54 | 11284 | 12 | CD69 | 0.167 | -0.223 | 0.545 | 0.086 | No |
| Type 1 diabetes | rs17696736 | G | n/a | 0.113 | 54 | 11284 | 12 | SH2B3 | 0.233 | -0.151 | 0.610 | 0.199 | No |
| Type 1 diabetes | rs1465788 | C | n/a | 0.841 | 54 | 11284 | 14 | Intergenic | -0.207 | -0.615 | 0.198 | 0.148 | No |
| Type 1 diabetes | rs4900384 | G | n/a | 0.375 | 54 | 11284 | 14 | Intergenic | 0.067 | -0.342 | 0.476 | 0.086 | No |
| Type 1 diabetes | rs3825932 | T | rs2870085 | 0.595 | 54 | 11284 | 15 | CTSH | -0.051 | -0.462 | 0.358 | 0.148 | No |
| Type 1 diabetes | rs4788084 | C | n/a | 0.221 | 54 | 11284 | 16 | IL27 | 0.154 | -0.239 | 0.545 | 0.086 | No |
| Type 1 diabetes | rs12708716 | A | n/a | 0.78 | 54 | 11284 | 16 | KIAA0350 | -0.152 | -0.536 | 0.236 | 0.207 | No |
| Type 1 diabetes | rs7202877 | G | n/a | 0.808 | 54 | 11284 | 16 | Intergenic | -0.319 | -1.040 | 0.400 | 0.247 | No |
| Type 1 diabetes | rs2290400 | C | n/a | 0.367 | 54 | 11284 | 17 | ORMDL3 | 0.065 | -0.315 | 0.446 | 0.140 | No |
| Type 1 diabetes | rs7221109 | C | rs757411 | 0.246 | 54 | 11284 | 17 | Intergenic | 0.138 | -0.261 | 0.531 | 0.049 | No |
| Type 1 diabetes | rs2542151 | G | n/a | 0.975 | 54 | 11284 | 18 | PTPN2 | -0.671 | -1.353 | 0.010 | 0.262 | No |
| Type 1 diabetes | rs763361 | T | n/a | 0.126 | 54 | 11284 | 18 | CD226 | 0.220 | -0.163 | 0.599 | 0.148 | No |
| Type 1 diabetes | rs425105 | T | n/a | 0.489 | 54 | 11284 | 19 | Intergenic | 0.007 | -0.511 | 0.525 | 0.148 | No |
| Type 1 diabetes | rs6679677 | A | n/a | 0.0827 | 54 | 11284 | 1 | PTPN11 | 0.396 | -0.166 | 0.968 | 0.637 | No |
| Type 1 diabetes | rs3024505 | G | n/a | 0.0621 | 54 | 11284 | 1 | IL10 | 0.488 | -0.139 | 1.112 | 0.174 | No |
| Type 1 diabetes | rs2281808 | C | rs1535883 | 0.195 | 54 | 11284 | 20 | Intergenic | 0.181 | -0.236 | 0.593 | 0.104 | No |
| Type 1 diabetes | rs9976767 | G | n/a | 0.971 | 54 | 11284 | 21 | UBASH3A | -0.381 | -0.777 | 0.020 | 0.148 | No |
| Type 1 diabetes | rs5753037 | T | n/a | 0.197 | 54 | 11284 | 22 | Intergenic | 0.167 | -0.211 | 0.548 | 0.095 | No |
| Type 1 diabetes | rs229541 | A | rs229527 | 0.246 | 54 | 11284 | 22 | C1QTNF6 | 0.132 | -0.247 | 0.511 | 0.104 | No |
| Type 1 diabetes | rs1990760 | T | n/a | 0.261 | 54 | 11284 | 2 | IFIH1 | 0.127 | -0.261 | 0.519 | 0.166 | No |
| Type 1 diabetes | rs10517086 | A | n/a | 0.438 | 54 | 11284 | 4 | Intergenic | 0.032 | -0.378 | 0.446 | 0.086 | No |
| Type 1 diabetes | rs11755527 | G | rs3757247 | 0.147 | 54 | 11284 | 6 | BACH2 | 0.202 | -0.174 | 0.582 | 0.122 | No |
| Type 1 diabetes | rs9388489 | G | n/a | 0.663 | 54 | 11284 | 6 | C6orf173 | -0.081 | -0.462 | 0.300 | 0.157 | No |
| Type 1 diabetes | rs7804356 | T | n/a | 0.0946 | 54 | 11284 | 7 | Intergenic | 0.325 | -0.163 | 0.811 | 0.131 | No |
| Type 1 diabetes | rs2664170 | G | n/a | 0.674 | 54 | 11284 | X | Intergenic | -0.119 | -0.528 | 0.293 | 0.148 | No |
| Type 2 diabetes | rs7903146 | T | n/a | 8.27E-07 | 778 | 3273 | 10 | TCF7L2 | 0.285 | 0.166 | 0.399 | 0.315 | Yes |
| Type 2 diabetes | rs5219 | T | n/a | 0.00573 | 778 | 3273 | 11 | KCNJ11 | 0.146 | 0.030 | 0.262 | 0.140 | Yes |
| Type 2 diabetes | rs5215 | C | n/a | 0.00515 | 778 | 3273 | 11 | KCNJ11 | 0.148 | 0.030 | 0.261 | 0.131 | Yes |
| Type 2 diabetes | rs8050136 | A | n/a | 0.00205 | 778 | 3273 | 16 | FTO | 0.164 | 0.051 | 0.274 | 0.207 | Yes |
| Type 2 diabetes | rs10923931 | T | rs2793831 | 0.00493 | 778 | 3273 | 1 | NOTCH2 | 0.228 | 0.051 | 0.400 | 0.122 | Yes |
| Type 2 diabetes | rs7578597 | T | n/a | 0.0349 | 778 | 3273 | 2 | THADA | 0.173 | -0.010 | 0.358 | 0.140 | Yes |
| Type 2 diabetes | rs4689388 | A | n/a | 0.0176 | 778 | 3273 | 4 | WFS1 | 0.122 | 0.010 | 0.236 | 0.148 | Yes |
| Type 2 diabetes | rs6931514 | G | rs7756992 | 0.0131 | 778 | 3273 | 6 | CDKAL1 | 0.138 | 0.020 | 0.262 | 0.223 | Yes |
| Type 2 diabetes | rs4712523 | G | n/a | 0.00858 | 778 | 3273 | 6 | CDKAL1 | 0.143 | 0.030 | 0.262 | 0.182 | Yes |
| Type 2 diabetes | rs864745 | T | rs1635852 | 0.048 | 778 | 3273 | 7 | JAZF1 | 0.093 | -0.020 | 0.207 | 0.095 | Yes |
| Type 2 diabetes | rs12779790 | G | rs11257655 | 0.52 | 778 | 3273 | 10 | CDC123 | -0.004 | -0.139 | 0.131 | 0.104 | No |
| Type 2 diabetes | rs5015480 | C | n/a | 0.36 | 778 | 3273 | 10 | HHEX | 0.021 | -0.095 | 0.128 | 0.157 | No |
| Type 2 diabetes | rs9300039 | C | n/a | 0.595 | 778 | 3273 | 11 | Intergenic | -0.024 | -0.223 | 0.174 | 0.392 | No |
| Type 2 diabetes | rs7961581 | C | rs1353362 | 0.393 | 778 | 3273 | 12 | TSPAN8 | 0.017 | -0.104 | 0.139 | 0.086 | No |
| Type 2 diabetes | rs2943641 | C | n/a | 0.328 | 778 | 3273 | 2 | LOC64673 | 0.027 | -0.086 | 0.139 | 0.174 | No |
| Type 2 diabetes | rs4607103 | C | rs4411878 | 0.466 | 778 | 3273 | 3 | ADAMTS9 | 0.005 | -0.122 | 0.128 | 0.086 | No |
| Type 2 diabetes | rs4402960 | T | n/a | 0.0885 | 778 | 3273 | 3 | IGF2BP2 | 0.081 | -0.041 | 0.199 | 0.157 | No |
| Type 2 diabetes | rs13266634 | C | n/a | 0.956 | 778 | 3273 | 8 | SLC30A8 | -0.104 | -0.223 | 0.020 | 0.113 | No |
| Type 2 diabetes | rs10811661 | T | n/a | 0.134 | 778 | 3273 | 9 | CDKN2A | 0.084 | -0.062 | 0.231 | 0.182 | No |
| Ulcerative colitis | rs11190140 | T | n/a | 0.0469 | 181 | 13100 | 10 | NKX2-3 | 0.177 | -0.030 | 0.385 | 0.182 | Yes |
| Ulcerative colitis | rs1317209 | A | n/a | 0.00442 | 181 | 13100 | 1 | RNF186 | 0.325 | 0.083 | 0.562 | 0.157 | Yes |
| Ulcerative colitis | rs3197999 | A | n/a | 0.015 | 181 | 13100 | 3 | MST1 | 0.239 | 0.020 | 0.462 | 0.182 | Yes |
| Ulcerative colitis | rs9268923 | C | rs2395185 | 0.00172 | 181 | 13100 | 6 | HLA | 0.352 | 0.117 | 0.598 | 0.372 | Yes |
| Ulcerative colitis | rs1558744 | A | n/a | 0.276 | 181 | 13100 | 12 | IFNG | 0.064 | -0.148 | 0.274 | 0.148 | No |
| Ulcerative colitis | rs1728785 | C | rs1645928 | 0.803 | 181 | 13100 | 16 | CDH1 | -0.106 | -0.351 | 0.139 | 0.157 | No |
| Ulcerative colitis | rs8067378 | A | n/a | 0.946 | 181 | 13100 | 17 | GSDMB | -0.169 | -0.378 | 0.041 | 0.113 | No |
| Ulcerative colitis | rs11209026 | G | n/a | 0.0577 | 181 | 13100 | 1 | IL23R | 0.389 | -0.105 | 0.880 | 0.582 | No |
| Ulcerative colitis | rs2201841 | A | n/a | 0.661 | 181 | 13100 | 1 | IL23R | -0.047 | -0.270 | 0.174 | 0.239 | No |
| Ulcerative colitis | rs3806308 | C | n/a | 0.214 | 181 | 13100 | 1 | RNF186 | 0.089 | -0.131 | 0.301 | 0.247 | No |
| Ulcerative colitis | rs6426833 | A | n/a | 0.118 | 181 | 13100 | 1 | RNF186 | 0.127 | -0.086 | 0.342 | 0.262 | No |
| Ulcerative colitis | rs10800309 | A | n/a | 0.243 | 181 | 13100 | 1 | FCGR2A | 0.077 | -0.140 | 0.301 | 0.182 | No |
| Ulcerative colitis | rs3024505 | A | n/a | 0.523 | 181 | 13100 | 1 | IL10 | -0.009 | -0.293 | 0.274 | 0.207 | No |
| Ulcerative colitis | rs6017342 | C | n/a | 0.345 | 181 | 13100 | 20 | HNF4A | 0.042 | -0.163 | 0.255 | 0.157 | No |
| Ulcerative colitis | rs5771069 | G | n/a | 0.0521 | 181 | 13100 | 22 | IL17REL | 0.173 | -0.041 | 0.378 | 0.157 | No |
| Ulcerative colitis | rs13003464 | G | n/a | 0.19 | 181 | 13100 | 2 | REL | 0.094 | -0.117 | 0.307 | 0.122 | No |
| Ulcerative colitis | rs4957048 | G | n/a | 0.634 | 181 | 13100 | 5 | CEP72 | -0.044 | -0.301 | 0.207 | 0.262 | No |
| Ulcerative colitis | rs7809799 | G | rs7788750 | 0.306 | 181 | 13100 | 7 | SMURF1 | 0.124 | -0.357 | 0.604 | 0.445 | No |
| Ulcerative colitis | rs4598195 | A | n/a | 0.374 | 181 | 13100 | 7 | DLD | 0.034 | -0.174 | 0.248 | 0.086 | No |
| Ulcerative colitis | rs4077515 | C | n/a | 0.571 | 181 | 13100 | 9 | CARD9 | -0.019 | -0.231 | 0.186 | 0.131 | No |
