# Supplementary material for: Efficient Replication of over 180 Genetic Associations with Self-Reported Medical Data
Source: PLoS One. 2011 Aug 17;6(8):e23473. doi: 10.1371/journal.pone.0023473 (PMC3157390; doi:10.1371/journal.pone.0023473)
Supplement: Table S2 — All quantitative replications attempted. Risk = risk allele for original SNP. Size = number of participants. Chr = chromosome. Beta = 23andMe beta (effect size). Rep = replicated. (DOCX) [file pone.0023473.s004.docx]

**Table S2**

| **Phenotype** | **SNP** | **Risk** | **Proxy SNP** | **P-value** | **Size** | **Chr** | **Region** | **Beta** | **Rep** |
| --- | --- | --- | --- | --- | --- | --- | --- | --- | --- |
| Birth weight | rs9883204 | C | rs6798189 | 0.078 | 7427 | 3 | ADCY5 | -0.598 (-2.28 - 1.09) | No |
| Birth weight | rs900400 | C | rs900399 | 0.000458 | 7427 | 3 | CCNL1 | -1.23 (-3.77 - 1.32) | Yes |
| BMI | rs10838738 | G | n/a | 0.231 | 10548 | 11 | MTCH2 | 0.0569 (-0.47 - 0.59) | No |
| BMI | rs925946 | T | n/a | 2.68E-06 | 10548 | 11 | BDNF | 0.367 (-0.22 - 0.95) | Yes |
| BMI | rs6265 | C | n/a | 9.58E-06 | 10548 | 11 | BDNF | 0.404 (-0.37 - 1.17) | Yes |
| BMI | rs7138803 | A | n/a | 0.0733 | 10548 | 12 | BCDIN3D | 0.111 (-0.25 - 0.47) | No |
| BMI | rs7498665 | G | rs4788102 | 0.00973 | 10548 | 16 | SH2B1 | 0.179 (-0.18 - 0.54) | Yes |
| BMI | rs8050136 | A | n/a | 1.26E-05 | 10548 | 16 | FTO | 0.317 (-0.05 - 0.68) | Yes |
| BMI | rs12970134 | A | n/a | 0.0162 | 10548 | 18 | MC4R | 0.18 (-0.14 - 0.5) | Yes |
| BMI | rs29941 | G | n/a | 0.00808 | 10548 | 19 | KCTD15 | 0.192 (-0.14 - 0.53) | Yes |
| BMI | rs2568958 | A | n/a | 0.79 | 10548 | 1 | NEGR1 | -0.062 (-0.58 - 0.45) | No |
| BMI | rs10913469 | C | n/a | 0.00232 | 10548 | 1 | SEC16B | 0.268 (-0.03 - 0.56) | Yes |
| BMI | rs7561317 | G | n/a | 2.51E-07 | 10548 | 2 | TMEM18 | 0.48 (0.18 - 0.78) | Yes |
| BMI | rs7647305 | C | n/a | 0.315 | 10548 | 3 | SFRS10 | 0.0435 (-0.67 - 0.76) | No |
| BMI | rs10938397 | G | rs13130484 | 0.106 | 10548 | 4 | GNPDA2 | 0.0926 (-0.37 - 0.55) | No |
| Hair curl | rs11803731 | A | rs17646946 | 2.76E-113 | 10644 | 1 | TCHH | 0.368 (0.35 - 0.38) | Yes |
| Hair color | rs4778138 | G | n/a | 5.47E-54 | 6301 | 15 | None | 0.756 (0.55 - 0.96) | Yes |
| Hair color | rs12913832 | A | n/a | 2.67E-203 | 6301 | 15 | HERC2 | 1.13 (1.06 - 1.2) | Yes |
| Hair color | rs258322 | A | n/a | 1.14E-42 | 6301 | 16 | MC1R | -0.786 (-0.85 - -0.72) | Yes |
| Hair color | rs28777 | C | rs16899932 | 0.2 | 6301 | 5 | MATP | 0.828 (0.77 - 0.89) | No |
| Hair color | rs12203592 | T | n/a | 4.07E-45 | 6301 | 6 | IRF4 | 0.622 (0.44 - 0.8) | Yes |
| Hair color (no red) | rs12896399 | G | n/a | 1.68E-24 | 4599 | 14 | SLC24A4 | 0.374 (0.29 - 0.46) | Yes |
| Hair color (no red) | rs4778138 | G | n/a | 5.91E-47 | 4599 | 15 | None | 0.741 (0.58 - 0.9) | Yes |
| Hair color (no red) | rs12913832 | A | n/a | 4.48E-194 | 4599 | 15 | HERC2 | 1.14 (1.08 - 1.2) | Yes |
| Hair color (no red) | rs28777 | C | rs16899932 | 0.331 | 4599 | 5 | MATP | 0.391 (0.34 - 0.44) | No |
| Hair color (no red) | rs6918152 | A | n/a | 5.56E-07 | 4599 | 6 | EXOC2 | -0.187 (-0.25 - -0.12) | Yes |
| Hair color (no red) | rs12203592 | T | n/a | 7.39E-50 | 4599 | 6 | IRF4 | 0.688 (0.55 - 0.83) | Yes |
| Height | rs8756 | C | n/a | 4.19E-07 | 10751 | 12 | HMGA2 | 0.315 (-0.79 - 1.42) | Yes |
| Height | rs11107116 | G | n/a | 0.00943 | 10751 | 12 | SOCS2 | -0.183 (-1.98 - 1.62) | Yes |
| Height | rs3116602 | G | rs3118914 | 0.0274 | 10751 | 13 | DLEU7 | -0.148 (-1.93 - 1.63) | Yes |
| Height | rs7153027 | A | n/a | 0.000897 | 10751 | 14 | TRIP11 | 0.2 (-0.96 - 1.36) | Yes |
| Height | rs2562784 | G | n/a | 0.0339 | 10751 | 15 | SH3GL3 | 0.137 (-1.56 - 1.84) | Yes |
| Height | rs4533267 | A | n/a | 0.17 | 10751 | 15 | ADAMTS17 | 0.0668 (-0.76 - 0.89) | No |
| Height | rs3760318 | G | n/a | 0.0109 | 10751 | 17 | CRLF3 | 0.152 (-0.75 - 1.06) | Yes |
| Height | rs4794665 | A | n/a | 0.0934 | 10751 | 17 | NOG | 0.084 (-0.95 - 1.12) | No |
| Height | rs757608 | A | n/a | 0.00436 | 10751 | 17 | BCAS3 | 0.177 (-0.69 - 1.05) | Yes |
| Height | rs4800148 | A | n/a | 0.00305 | 10751 | 18 | CABLES1 | 0.216 (-1.61 - 2.04) | Yes |
| Height | rs12735613 | A | rs17038164 | 0.00561 | 10751 | 1 | SPAG17 | -0.187 (-0.99 - 0.62) | Yes |
| Height | rs6686842 | C | rs6663565 | 0.437 | 10751 | 1 | SCMH1 | -0.0102 (-0.97 - 0.95) | No |
| Height | rs11809207 | A | rs4659415 | 0.13 | 10751 | 1 | CATSPER4 | 0.0874 (-1.71 - 1.89) | No |
| Height | rs678962 | G | n/a | 0.453 | 10751 | 1 | DNM3 | 0.00906 (-0.77 - 0.78) | No |
| Height | rs2274432 | A | rs1046934 | 0.0263 | 10751 | 1 | C1orf19 | 0.13 (-1.22 - 1.48) | Yes |
| Height | rs1390401 | A | rs6656661 | 0.544 | 10751 | 1 | ZNF678 | -0.00911 (-2.01 - 1.99) | No |
| Height | rs967417 | G | n/a | 0.00588 | 10751 | 20 | BMP2 | 0.161 (-0.83 - 1.15) | Yes |
| Height | rs6060369 | C | rs6088813 | 0.335 | 10751 | 20 | GDF5 | 0.028 (-1.21 - 1.27) | No |
| Height | rs3791679 | A | n/a | 0.000282 | 10751 | 2 | EFEMP1 | 0.258 (-1.43 - 1.94) | Yes |
| Height | rs6724465 | A | rs7600987 | 0.0753 | 10751 | 2 | IHH | -0.154 (-2.94 - 2.63) | No |
| Height | rs6717918 | T | rs6718438 | 0.165 | 10751 | 2 | DIS3L2 | 0.0759 (-0.7 - 0.85) | No |
| Height | rs10935120 | A | rs9841212 | 0.052 | 10751 | 3 | ANAPC13 | -0.11 (-0.98 - 0.76) | No |
| Height | rs6763931 | A | n/a | 0.00141 | 10751 | 3 | ZBTB38 | 0.192 (-0.78 - 1.16) | Yes |
| Height | rs6830062 | T | n/a | 0.000277 | 10751 | 4 | LCORL | 0.295 (-0.44 - 1.03) | Yes |
| Height | rs710841 | T | rs1443537 | 0.0877 | 10751 | 4 | PRKG2 | 0.0997 (-0.7 - 0.9) | No |
| Height | rs1812175 | G | n/a | 0.0149 | 10751 | 4 | HHIP | 0.186 (-0.56 - 0.93) | Yes |
| Height | rs4713858 | G | n/a | 0.0015 | 10751 | 6 | ANKS1A | 0.26 (-0.47 - 0.99) | Yes |
| Height | rs1776897 | G | n/a | 0.0222 | 10751 | 6 | HMGA1 | 0.22 (-0.48 - 0.92) | Yes |
| Height | rs2814993 | A | n/a | 0.0343 | 10751 | 6 | C6orf106 | 0.162 (-0.57 - 0.89) | Yes |
| Height | rs185819 | T | n/a | 0.117 | 10751 | 6 | HLA | 0.0754 (-0.99 - 1.14) | No |
| Height | rs13437082 | T | n/a | 0.0247 | 10751 | 6 | HLA | -0.139 (-1.68 - 1.4) | Yes |
| Height | rs10946808 | A | n/a | 0.000101 | 10751 | 6 | HIST1H1D | 0.255 (-1.18 - 1.69) | Yes |
| Height | rs12198986 | A | n/a | 0.017 | 10751 | 6 | BMP6 | 0.136 (-0.87 - 1.14) | Yes |
| Height | rs314277 | A | n/a | 0.0649 | 10751 | 6 | LIN28B | 0.134 (-0.6 - 0.87) | No |
| Height | rs4549631 | C | rs9401888 | 0.109 | 10751 | 6 | LOC387103 | 0.0782 (-0.96 - 1.12) | No |
| Height | rs3748069 | A | n/a | 0.0213 | 10751 | 6 | GPR126 | 0.142 (-1.34 - 1.62) | Yes |
| Height | rs1635852 | T | n/a | 0.118 | 10751 | 7 | JAZF1 | 0.0758 (-0.99 - 1.14) | No |
| Height | rs798544 | C | n/a | 0.0121 | 10751 | 7 | GNA12 | 0.158 (-1.34 - 1.66) | Yes |
| Height | rs2282978 | C | n/a | 0.0849 | 10751 | 7 | CDK6 | 0.0913 (-0.79 - 0.98) | No |
| Height | rs10958476 | C | n/a | 0.0392 | 10751 | 8 | PLAG1 | 0.138 (-0.63 - 0.91) | Yes |
| Height | rs7846385 | C | n/a | 0.357 | 10751 | 8 | PXMP3 | 0.0258 (-0.8 - 0.85) | No |
| Height | rs10512248 | G | rs4448343 | 0.00353 | 10751 | 9 | PTCH1 | 0.181 (-1.17 - 1.53) | Yes |
| Height | rs4743034 | A | n/a | 0.112 | 10751 | 9 | ZNF462 | 0.0923 (-0.69 - 0.87) | No |
| Tanning | rs10831496 | G | n/a | 7.08E-06 | 4611 | 11 | GRM5 | 0.35 (0.08 - 0.62) | Yes |
| Tanning | rs1393350 | A | n/a | 6.59E-09 | 4611 | 11 | TYR | -0.478 (-0.63 - -0.33) | Yes |
| Tanning | rs17094273 | A | n/a | 0.0246 | 4611 | 14 | Intergenic | 0.246 (0.12 - 0.38) | Yes |
| Tanning | rs11648785 | T | n/a | 2.56E-15 | 4611 | 16 | MC1R | 0.634 (0.37 - 0.9) | Yes |
| Tanning | rs154659 | C | n/a | 1.35E-08 | 4611 | 16 | MC1R | -0.473 (-0.62 - -0.33) | Yes |
| Tanning | rs35391 | T | n/a | 3.02E-33 | 4611 | 5 | MATP | 2.69 (1.71 - 3.68) | Yes |
| Tanning | rs12210050 | T | n/a | 7.56E-15 | 4611 | 6 | EXOC2 | -0.741 (-1.11 - -0.38) | Yes |
