# Supplementary material for: Efficient Replication of over 180 Genetic Associations with Self-Reported Medical Data
Source: PLoS One. 2011 Aug 17;6(8):e23473. doi: 10.1371/journal.pone.0023473 (PMC3157390; doi:10.1371/journal.pone.0023473)
Supplement: Table S3 — All successful replications without strictly matching phenotypes. Phenotype = 23andMe phenotype. Published = published phenotype. OR = 23andMe odds ratio. a Note that this replication is in the opposite direction from that reported in the GWAS catalog. However, a close reading of the original report shows that the direction of the effect was misreported in the GWAS catalog. (DOCX) [file pone.0023473.s005.docx]

**Table S3**

| **Phenotype** | **Published** | **Rsid** | **Region** | **Cases** | **Controls** | **P-value** | **OR** |
| --- | --- | --- | --- | --- | --- | --- | --- |
| Gall bladder removal | Gallstones | rs11887534 | ABCG8 | 733 | 10175 | 2.05E-18 | 2.08 (1.75 - 2.44) |
| High blood pressure | Blood pressure | rs3184504 | SH2B3 | 2878 | 8191 | 0.0205 | 1.06 (1 - 1.13) |
| High blood pressure | Blood pressure | rs3934103 | ULK4 | 2878 | 8191 | 0.0223 | 1.08 (1 - 1.18) |
| High blood pressure | Blood pressure | rs2681472 | ATP2B1 | 2878 | 8191 | 0.013 | 1.1 (1.01 - 1.19) |
| High blood pressure | Blood pressure | rs17637472 | ZNF652 | 2878 | 8191 | 0.0137 | 1.07 (1.01 - 1.14) |
| High blood pressure | Blood pressure | rs1378942 | CYP1A1 | 2878 | 8191 | 0.0111 | 1.08 (1.01 - 1.15) |
| High blood pressure | Blood pressure | rs1458038 | FGF5 | 2878 | 8191 | 0.000276 | 1.12 (1.05 - 1.2) |
| High blood pressure | Blood pressure | rs1004467 | CYP17A1 | 2878 | 8191 | 0.0012 | 1.18 (1.06 - 1.3) |
| High blood pressure | Blood pressure | rs12413409 | CYP17A1 | 2878 | 8191 | 0.00196 | 1.18 (1.05 - 1.31) |
| High cholesterol | Cholesterol levels | rs10889353 | DOCK7 | 3621 | 7394 | 0.00268 | 0.917 (0.86 - 0.97) |
| High cholesterol | Cholesterol levels | rs2228671 | LDLR | 3621 | 7394 | 1.11E-09 | 1.32 (1.2 - 1.45) |
| High cholesterol | Cholesterol levels | rs1800588 | LIPC | 3621 | 7394 | 0.000828 | 1.12 (1.04 - 1.19) |
| High cholesterol | Cholesterol levels | rs10468017 | LIPC | 3621 | 7394 | 0.0226 | 1.07 (1 - 1.13) |
| High cholesterol | Cholesterol levels | rs11206510 | PCSK9 | 3621 | 7394 | 0.00854 | 1.09 (1.02 - 1.17) |
| High cholesterol | Cholesterol levels | rs12610185 | CILP2 | 3621 | 7394 | 4.81E-05 | 1.24 (1.11 - 1.38) |
| High cholesterol | Cholesterol levels | rs11591147 | PCSK9 | 3621 | 7394 | 0.000693 | 0.664 (0.52 - 0.85) |
| High cholesterol | Cholesterol levels | rs2228603 | NCAN | 3621 | 7394 | 0.00235 | 0.853 (0.76 - 0.95) |
| High cholesterol | Cholesterol levels | rs6102051 | MAFB | 3621 | 7394 | 0.0206 | 0.938 (0.88 - 1) |
| High cholesterol | Cholesterol levels | rs1169288 | HNF1A | 3621 | 7394 | 0.00225 | 1.09 (1.03 - 1.16) |
| High cholesterol | Cholesterol levels | rs6544713 | ABCG8 | 3621 | 7394 | 0.0427 | 1.05 (0.99 - 1.12) |
| High cholesterol | Cholesterol levels | rs541041 | APOB | 3621 | 7394 | 8.39E-08 | 0.82 (0.76 - 0.88) |
| High cholesterol | Cholesterol levels | rs646776 | CELSR2 | 3621 | 7394 | 3.83E-11 | 0.794 (0.741 - 0.855) |
| High cholesterol | Cholesterol levels | rs174546 | FADS1 | 3621 | 7394 | 0.0129 | 0.934 (0.88 - 0.99) |
| High cholesterol | Cholesterol levels | rs157580 | TOMM40 | 3621 | 7394 | 1.39E-05 | 0.884 (0.83 - 0.94) |
| High cholesterol | Cholesterol levels | rs12670798 | DNAH11 | 3621 | 7394 | 0.00589 | 1.09 (1.02 - 1.16) |
| High cholesterol | Cholesterol levels | rs12272004 | APOA1 | 3621 | 7394 | 0.0137 | 0.886 (0.8 - 0.99) |
| High cholesterol | Cholesterol levels | rs6982636 | TRIB1 | 3621 | 7394 | 2.85E-07 | 1.15 (1.09 - 1.22) |
| High cholesterol | Cholesterol levels | rs1260326 | GCKR | 3621 | 7394 | 1.38E-05 | 1.13 (1.07 - 1.2) |
| Liver test | Bilirubin levels | rs6519520 | GGT1 | 913 | 10079 | 0.00111 | 1.17 (1.06 - 1.3) |
| Liver test | Bilirubin levels | rs926633 | PNPLA3 | 913 | 10079 | 0.00495 | 1.18 (1.04 - 1.33) |
| Liver test | Bilirubin levels | rs3790567 | IL12RB2 | 913 | 10079 | 0.044 | 1.1 (0.99 - 1.23) |
| Liver test | Bilirubin levels | rs887829 | UGT1A1 | 913 | 10079 | 1.22E-06 | 1.27 (1.15 - 1.4) ^a^ |
| Macular degeneration | Advanced AMD | rs2230199 | C3 | 221 | 4106 | 0.0234 | 1.26 (1 - 1.56) |
| Macular degeneration | Advanced AMD | rs429608 | C2 | 221 | 4106 | 0.0175 | 1.38 (1.02 - 1.86) |
| Nicotine abuse | Nicotine dependence | rs1051730 | CHRNA3 | 1944 | 10685 | 0.000986 | 0.89 (0.83 – 0.96) |
| Osteoporosis | Bone density | rs1366594 | MEF2C | 651 | 10294 | 0.00365 | 1.17 (1.04 - 1.31) |
| Osteoporosis | Bone density | rs1385162 | SOX6 | 651 | 10294 | 0.0212 | 0.861 (0.746 - 0.99) |
| Osteoporosis | Bone density | rs10876432 | OSX | 651 | 10294 | 0.0387 | 1.12 (0.99 - 1.28) |
| Osteoporosis | Bone density | rs11898505 | SPTBN1 | 651 | 10294 | 0.0331 | 0.895 (0.794 - 1.01) |
| Osteoporosis | Bone density | rs1471403 | MEPE | 651 | 10294 | 0.013 | 0.871 (0.77 - 0.98) |
