# Supplementary material for: Efficient Replication of over 180 Genetic Associations with Self-Reported Medical Data
Source: PLoS One. 2011 Aug 17;6(8):e23473. doi: 10.1371/journal.pone.0023473 (PMC3157390; doi:10.1371/journal.pone.0023473)
Supplement: Table S4 — Effect of viewing genetic risk data on reported disease status. Because one aspect of 23andMe's Personal Genome Service involves returning genetic data to our customers, it is possible that viewing a result of elevated risk for a certain disease may make it more likely for an individual to recall a previous diagnosis of that disease, thus potentially skewing the results towards replication. To address this, we investigated the impact of seeing genetic risk results before versus after answering survey questions on self-reported disease status for a set of 20 conditions for which participants were able to view a personal risk prediction. Only for psoriasis was there a statistically significant impact of seeing one's results on self-report of disease status, and this impact was no longer observed once the direction of the estimated risk (increased or decreased) was taken into account, suggesting that in general, the nature of the genetic risk result did not have a consistent or significant effect on the way questions were answered. Estimated Risk = p-value for association of estimated risk with reported disease status. We expect to see an association with any risk model that is reasonably predictive. Saw Data First = p-value for association of viewing genetic risk results before answering survey questions with reported disease status. Estimated Risk * Saw Data First = p-value for association of interaction between estimated risk and viewing this risk before answering survey questions with reported disease status. (DOCX) [file pone.0023473.s006.docx]

**Table S4**

| **Phenotype** | **Estimated Risk** | **Saw Data First** | **Estimated Risk * Saw Data First** |
| --- | --- | --- | --- |
| Heart attack | 0.00178 | 0.0665 | 0.039 |
| Breast cancer | 4.00E-05 | 0.305 | 0.121 |
| Parkinson's disease | 0.55 | 0.179 | 0.787 |
| Bipolar disorder | 0.811 | 0.174 | 0.183 |
| Crohn's disease | 0.77 | 0.175 | 0.199 |
| Rheumatoid arthritis | 0.0388 | 0.819 | 0.199 |
| Obesity | 6.51E-05 | 0.169 | 0.216 |
| Type 1 diabetes | 0.000349 | 0.318 | 0.23 |
| Psoriasis | 2.71E-08 | 0.209 | 0.261 |
| Lung cancer | 0.758 | 0.403 | 0.324 |
| Macular degeneration | 0.000133 | 0.0486 | 0.328 |
| Prostate cancer | 6.73E-08 | 0.0783 | 0.411 |
| Ulcerative colitis | 0.156 | 0.795 | 0.51 |
| Type 1 diabetes | 1.37E-05 | 0.851 | 0.562 |
| Colorectal cancer | 0.837 | 0.864 | 0.743 |
| Celiac disease | 0.000161 | 0.103 | 0.873 |
| Lupus | 0.335 | 0.447 | 0.9 |
| Glaucoma | 0.589 | 0.791 | 0.912 |
