# Supplementary material for: Efficient Replication of over 180 Genetic Associations with Self-Reported Medical Data
Source: PLoS One. 2011 Aug 17;6(8):e23473. doi: 10.1371/journal.pone.0023473 (PMC3157390; doi:10.1371/journal.pone.0023473)
Supplement: Table S5 — Success rate by replication p-value threshold. Alpha = p-value threshold for replication. Replications = number of associations successfully replicated. Expected = number of associations we expected to replicate. Ratio = expected / replications. These calculations do not include the nine associations for which our power may be substantially overestimated. (DOCX) [file pone.0023473.s007.docx]

**Table S5**

| **Alpha** | **Replications** | **Expected** | **Ratio** |
| --- | --- | --- | --- |
| 0.5 | 219 | 258 | 0.848 |
| 0.25 | 164 | 208 | 0.79 |
| 0.1 | 114 | 154 | 0.74 |
| 0.05 | 93 | 124 | 0.75 |
| 0.01 | 57 | 79.8 | 0.714 |
| 0.001 | 35 | 50.2 | 0.697 |
| 0.0001 | 30 | 37 | 0.812 |
| 1.00E-05 | 24 | 29.9 | 0.802 |
| 1.00E-06 | 22 | 25.6 | 0.861 |
| 1.00E-07 | 19 | 22.5 | 0.845 |
| 1.00E-08 | 19 | 20.2 | 0.94 |
| 1.00E-09 | 16 | 18.5 | 0.865 |
