# Supplementary material for: Efficient Replication of over 180 Genetic Associations with Self-Reported Medical Data
Source: PLoS One. 2011 Aug 17;6(8):e23473. doi: 10.1371/journal.pone.0023473 (PMC3157390; doi:10.1371/journal.pone.0023473)
Supplement: Table S6 — Additional parameters for phenotype classification. (DOCX) [file pone.0023473.s008.docx]

**Table S6**

| **Phenotype** | **Additional parameters** |
| --- | --- |
| Alcohol abuse | Controls: age >= 34 |
| Alzheimer's disease | Controls: age >= 85 |
| Basal cell carcinoma | Controls: age >= 54 |
| Breast cancer | Controls: age >= 56; All subjects: sex = female |
| Coronary artery disease | Controls: age >= 70 |
| Colorectal cancer | Controls: age >= 60 |
| Chronic obstructive pulmonary disease (COPD) | All subjects: history of smoking |
| Heart attack, early onset | Cases: age <= 50 if male, age <= 60 if female; Controls: age >= 59 |
| Prostate cancer, early onset | Cases: age <= 60; Controls: age >= 59; All subjects: sex = M |
| Autism | Cases: age <= 18 |
| Macular degeneration | Controls: age >= 60 |
| Male pattern baldness | Controls: age >= 40; All subjects: sex = male |
| Osteoarthritis | Controls: age >= 73 |
| Ovarian cancer | All subjects: sex = female |
| Parkinson's disease | Controls: age >= 50 |
| Inflammatory bowel disease (IBD), juvenile onset | Cases: age <= 18 |
| Prostate cancer | Controls: age >= 68; All subjects: sex = male |
| Psoriasis | Controls: age >= 60 |
| Type 2 diabetes | Controls: age > = 62 and no gestational diabetes |
| Testicular cancer | All subjects: sex = male |
