# Supplementary material for: Efficient Replication of over 180 Genetic Associations with Self-Reported Medical Data
Source: PLoS One. 2011 Aug 17;6(8):e23473. doi: 10.1371/journal.pone.0023473 (PMC3157390; doi:10.1371/journal.pone.0023473)
Supplement: Table S7 — Data from GWAS catalog for associations with strictly matching phenotypes. PMID: PubMed ID. RAF = risk allele frequency. OR/Beta = odds ratio or beta (effect size). CI = confidence interval. (DOCX) [file pone.0023473.s009.docx]

**Table S7**

| **PMID** | **Phenotype** | **Region** | **SNP-Risk allele** | **RAF** | **P-value** | **OR/Beta** | **CI** |
| --- | --- | --- | --- | --- | --- | --- | --- |
| 19581569 | Alcohol dependence | PECR | rs7590720-G | 0.29 | 1.00E-08 | 1.35 | [1.22-1.49] |
| 19734902 | Alzheimer's disease | PICALM | rs3851179-? | 0.63 | 1.00E-09 | 1.16 | [1.11-1.22] |
| 19734902 | Alzheimer's disease | APOE, TOMM40 | rs2075650-? | 0.15 | 2.00E-157 | 2.53 | [2.37-2.71] |
| 17611496 | Asthma | ORMDL3 | rs7216389-T | 0.52 | 9.00E-11 | 1.45 | [1.17-1.81] |
| 20032318 | Asthma | DENND1B, CRB1 | rs2786098-? | 0.85 | 2.00E-13 | 1.43 | [NR] (European ancestry) |
| 19426955 | Asthma | PDE4D | rs1588265-C | 0.29 | 3.00E-08 | 1.18 | [1.08-1.30] |
| 19404256 | Autism | CDH10,CDH9 | rs4307059-T | 0.61 | 2.00E-10 | 1.19 | [NR] |
| 18849993 | Basal cell carcinoma (cutaneous) | PADI4, PADI6,RCC2, ARHGEF10L | rs7538876-A | 0.35 | 4.00E-12 | 1.28 | [1.19-1.37] |
| 18849993 | Basal cell carcinoma (cutaneous) | RHOU | rs801114-G | 0.33 | 6.00E-12 | 1.28 | [1.19-1.37] |
| 18711365 | Bipolar disorder | ANK3 | rs10994336-T | 0.05 | 9.00E-09 | 1.45 | [NR] |
| 18711365 | Bipolar disorder | CACNA1C | rs1006737-A | 0.32 | 7.00E-08 | 1.18 | [NR] |
| 17486107 | Bipolar disorder | DGKH | rs1012053-A | 0.84 | 2.00E-08 | 1.59 | [1.35-1.87] |
| 17554300 | Bipolar disorder | PALB2,NDUFAB1,DCTN5 | rs420259-A | 0.72 | 6.00E-08 | 2.08 | [1.60-2.71] |
| 19416921 | Bipolar disorder | MCTP1 | rs17418283-C | 0.28 | 1.00E-07 | 1.21 | [1.13-1.30] |
| 20372150 | Birth weight | ADCY5 | rs9883204-C | NR | 7.00E-15 | 0.06 | [0.047-0.079] sd decrease |
| 20372150 | Birth weight | CCNL1, LEKR1 | rs900400-C | NR | 2.00E-35 | 0.09 | [0.073-0.10] sd decrease |
| 18483556 | Black vs. blond hair color | SLC24A4 | rs12896399-G | NR | 8.00E-21 | 0.18 | [0.14-0.22] increase (WRONG: DECREASE) in hair color score |
| 18483556 | Black vs. blond hair color | OCA2 | rs11855019-G | NR | 2.00E-24 | 0.28 | [0.22-0.34] decrease in hair color score |
| 18483556 | Black vs. blond hair color | HERC2 | rs12913832-A | NR | 4.00E-103 | 0.44 | [0.40-0.48] decrease in hair color score |
| 18483556 | Black vs. blond hair color | MATP | rs28777-C | NR | 1.00E-17 | 0.46 | [0.36-0.56] decrease in hair color score |
| 18483556 | Black vs. blond hair color | EXOC2 | rs6918152-A | NR | 6.00E-08 | 0.11 | [0.07-0.15] increase in hair color score |
| 18483556 | Black vs. blond hair color | IRF4 | rs12203592-T | NR | 7.00E-127 | 0.35 | [0.33-0.37] decrease in hair color score |
| 18483556 | Black vs. red hair color | OCA2 | rs11855019-G | NR | 6.00E-20 | 0.29 | [0.23-0.35] decrease in hair color score |
| 18483556 | Black vs. red hair color | HERC2 | rs12913832-A | NR | 1.00E-77 | 0.44 | [0.40-0.48] decrease in hair color score |
| 18483556 | Black vs. red hair color | MC1R | rs258322-T | NR | 2.00E-23 | 0.36 | [0.28-0.44] increase in hair color score |
| 18483556 | Black vs. red hair color | MATP | rs28777-C | NR | 9.00E-14 | 0.46 | [0.34-0.58] decrease in hair color score |
| 18483556 | Black vs. red hair color | IRF4 | rs12203592-T | NR | 9.00E-28 | 0.31 | [0.25-0.36] decrease in hair color score |
| 19648920 | Bladder cancer | PSCA | rs2294008-T | 0.46 | 2.00E-10 | 1.15 | [1.10-1.20] |
| 17952075 | Blond vs. brown hair color | KITLG | rs12821256-C | 0.14 | 4.00E-30 | 2.32 | [1.86-2.92] |
| 17952075 | Blond vs. brown hair color | SLC24A4 | rs12896399-T | 0.44 | 1.00E-48 | 2.56 | [2.12-3.09] |
| 17952075 | Blond vs. brown hair color | OCA2 | rs1667394-A | 0.88 | 6.00E-35 | 4.94 | [3.16-7.71] |
| 17952075 | Blond vs. brown hair color | MC1R | rs1805007-T | 0.08 | 2.00E-13 | 2.34 | [1.69-3.24] |
| 17952075 | Blue vs. brown eyes | OCA2 | rs1667394-A | 0.88 | 1.00E-241 | 29.43 | [21.47-40.35] |
| 17952075 | Blue vs. green eyes | TYR | rs1393350-A | 0.23 | 3.00E-12 | 1.52 | [1.28-1.81] |
| 17952075 | Blue vs. green eyes | SLC24A4 | rs12896399-T | 0.4 | 4.00E-38 | 2.06 | [1.76-2.42] |
| 17952075 | Blue vs. green eyes | OCA2 | rs1667394-A | 0.98 | 2.00E-53 | 6.74 | [4.61-9.83] |
| 18488028 | Blue vs. green eyes | TYRP1 | rs1408799-C | 0.75 | 6.00E-17 | 1.4 | [1.25-1.57] |
| 19079261 | Body mass index | MTCH2 | rs10838738-G | 0.34 | 5.00E-09 | 0.07 | [0.01-0.13] kg/m2 increase |
| 19079260 | Body mass index | BDNF | rs925946-T | 0.34 | 9.00E-10 | 3.85 | [2.62-5.08] % SD |
| 19079260 | Body mass index | BDNF | rs6265-G | 0.85 | 5.00E-10 | 4.58 | [3.07-6.09] % SD |
| 19079260 | Body mass index | BCDIN3D, FAIM2 | rs7138803-A | 0.37 | 1.00E-07 | 3.28 | [2.06-4.50] % SD |
| 19079260 | Body mass index | SH2B1, ATP2A1 | rs7498665-G | 0.44 | 3.00E-10 | 3.63 | [2.49-4.77] % SD |
| 19079260 | Body mass index | FTO | rs8050136-A | 0.41 | 1.00E-47 | 8.04 | [6.96-9.12] % SD |
| 19079260 | Body mass index | MC4R | rs12970134-A | 0.3 | 1.00E-12 | 4.38 | [3.16-5.60] % SD |
| 19079260 | Body mass index | KCTD15, CHST8 | rs29941-C | 0.69 | 7.00E-12 | 4.18 | [2.98-5.38] % SD |
| 19079260 | Body mass index | NEGR1 | rs2568958-A | 0.58 | 1.00E-11 | 3.77 | [2.67-4.87] % SD |
| 19079260 | Body mass index | SEC16B, RASAL2 | rs10913469-C | 0.2 | 6.00E-08 | 3.36 | [2.14-4.58] % SD |
| 19079260 | Body mass index | TMEM18 | rs7561317-G | 0.84 | 4.00E-17 | 6.12 | [4.69-7.55] % SD |
| 19079260 | Body mass index | SFRS10, ETV5, DGKG | rs7647305-C | 0.77 | 7.00E-11 | 4.42 | [3.09-5.75] % SD |
| 19079261 | Body mass index | GNPDA2 | rs10938397-G | 0.45 | 3.00E-16 | 0.19 | [0.13-0.25] kg/m2 |
| 17529967 | Breast cancer | FGFR2 | rs2981582-G | 0.38 | 2.00E-76 | 1.26 | [1.23-1.30] |
| 17529967 | Breast cancer | LSP1 | rs3817198-C | 0.3 | 3.00E-09 | 1.07 | [1.04-1.11] |
| 17529967 | Breast cancer | TNRC9, LOC643714 | rs3803662-T | 0.25 | 1.00E-36 | 1.2 | [1.16-1.24] |
| 19330030 | Breast cancer | Intergenic | rs11249433-C | 0.39 | 7.00E-10 | 1.16 | [1.09-1.24] |
| 19330030 | Breast cancer | Intergenic | rs13387042-A | 0.51 | 2.00E-08 | 1.25 | [1.15-1.37] |
| 17529967 | Breast cancer | MAP3K1 | rs889312-A | 0.28 | 7.00E-20 | 1.13 | [1.10-1.16] |
| 17529967 | Breast cancer | Intergenic | rs13281615-T | 0.4 | 5.00E-12 | 1.08 | [1.05-1.11] |
| 20190752 | Celiac disease | ZMIZ1 | rs1250552-? | 0.53 | 9.00E-10 | 1.12 | [1.09-1.16] |
| 20190752 | Celiac disease | ETS1 | rs11221332-A | 0.24 | 5.00E-16 | 1.21 | [1.16-1.27] |
| 20190752 | Celiac disease | SH2B3 | rs653178-G | 0.5 | 7.00E-21 | 1.2 | [1.15-1.24] |
| 20190752 | Celiac disease | CIITA, SOCS1, CLEC16A | rs12928822-? | 0.84 | 3.00E-08 | 1.16 | [1.10-1.22] |
| 20190752 | Celiac disease | PTPN2 | rs1893217-G | 0.17 | 3.00E-10 | 1.17 | [1.12-1.23] |
| 20190752 | Celiac disease | NFIA | rs6691768-? | 0.62 | 1.00E-07 | 1.11 | [1.06-1.15] |
| 20190752 | Celiac disease | RUNX3 | rs10903122-? | 0.52 | 2.00E-10 | 1.12 | [1.09-1.18] |
| 20190752 | Celiac disease | PARK7, TNFRSF9 | rs12727642-A | 0.19 | 9.00E-08 | 1.14 | [1.09-1.20] |
| 20190752 | Celiac disease | TNFRSF14, MMEL1 | rs3748816-? | 0.66 | 3.00E-09 | 1.12 | [1.09-1.18] |
| 20190752 | Celiac disease | RGS1 | rs2816316-? | 0.84 | 2.00E-17 | 1.25 | [1.19-1.32] |
| 20190752 | Celiac disease | Intergenic | rs296547-? | 0.64 | 4.00E-09 | 1.12 | [1.09-1.16] |
| 20190752 | Celiac disease | ICOSLG | rs4819388-? | 0.72 | 2.00E-09 | 1.14 | [1.09-1.19] |
| 20190752 | Celiac disease | PLEK | rs17035378-? | 0.72 | 8.00E-09 | 1.14 | [1.09-1.19] |
| 20190752 | Celiac disease | REL, AHSA2 | rs13003464-G | 0.4 | 4.00E-13 | 1.15 | [1.11-1.20] |
| 20190752 | Celiac disease | IL18RAP, IL18R1, IL1RL1, IL1RL2 | rs917997-A | 0.24 | 1.00E-15 | 1.19 | [1.14-1.25] |
| 20190752 | Celiac disease | ITGA4, UBE2E3 | rs13010713-G | 0.45 | 5.00E-11 | 1.13 | [1.09-1.18] |
| 20190752 | Celiac disease | CTLA4, ICOS, CD28 | rs4675374-A | 0.22 | 6.00E-09 | 1.14 | [1.09-1.19] |
| 20190752 | Celiac disease | CCR1, CCR2, CCRL2, CCR3, CCR5, CCR9 | rs13098911-A | 0.1 | 3.00E-17 | 1.3 | [1.23-1.39] |
| 20190752 | Celiac disease | CCR4 | rs13314993-C | 0.46 | 3.00E-09 | 1.13 | [1.08-1.17] |
| 20190752 | Celiac disease | CD80, KTELC1 | rs11712165-C | 0.39 | 8.00E-09 | 1.13 | [1.08-1.17] |
| 20190752 | Celiac disease | IL12A | rs17810546-G | 0.13 | 4.00E-28 | 1.36 | [1.29-1.44] |
| 20190752 | Celiac disease | LPP | rs1464510-A | 0.49 | 3.00E-40 | 1.29 | [1.25-1.34] |
| 18311140 | Celiac disease | KIAA1109, ADAD1, IL2, IL21 | rs6822844-C | 0.81 | 3.00E-13 | 1.44 | [1.30-1.58] |
| 20190752 | Celiac disease | HLA-DQA1, HLA-DQB1 | rs2187668-A | 0.26 | 1.00E-50 | 6.23 | [5.95-6.52] |
| 20190752 | Celiac disease | IRF4 | rs1033180-A | 0.08 | 6.00E-08 | 1.21 | [1.13-1.29] |
| 20190752 | Celiac disease | BACH2, MAP3K7 | rs10806425-A | 0.4 | 4.00E-10 | 1.13 | [1.09-1.17] |
| 20190752 | Celiac disease | PTPRK, THEMIS | rs802734-G | 0.31 | 3.00E-14 | 1.17 | [1.12-1.22] |
| 20190752 | Celiac disease | TNFAIP3 | rs2327832-G | 0.22 | 4.00E-19 | 1.23 | [1.17-1.28] |
| 20190752 | Celiac disease | TAGAP | rs1738074-A | 0.43 | 3.00E-15 | 1.16 | [1.12-1.21] |
| 20190752 | Celiac disease | Intergenic | rs9792269-? | 0.76 | 3.00E-09 | 1.14 | [1.10-1.19] |
| 20190752 | Celiac disease | TLR7, TLR8 | rs5979785-? | 0.74 | 6.00E-08 | 1.14 | [1.09-1.19] |
| 20173748 | Chronic obstructive pulmonary disease | CHRNA3, CHRNA5, IREB2 | rs13180-? | 0.36 | 2.00E-08 | 1.3 | [1.18-1.43] |
| 20173748 | Chronic obstructive pulmonary disease | FAM13A | rs7671167-? | 0.48 | 1.00E-11 | 1.32 | [1.19-1.47] |
| 19300482 | Chronic obstructive pulmonary disease | HHIP | rs1828591-? | NR | 1.00E-07 | 1.38 | [1.17-1.63] |
| 18372905 | Colorectal cancer | Intergenic | rs10795668-A | 0.67 | 3.00E-13 | 1.12 | [1.10-1.16] |
| 18372901 | Colorectal cancer | Intergenic | rs3802842-C | 0.43 | 6.00E-10 | 1.11 | [1.08-1.15] |
| 19011631 | Colorectal cancer | BMP4 | rs4444235-C | 0.46 | 8.00E-10 | 1.11 | [1.08-1.15] |
| 19011631 | Colorectal cancer | CDH1 | rs9929218-A | 0.29 | 1.00E-08 | 1.1 | [1.06-1.12] |
| 18372901 | Colorectal cancer | SMAD7 | rs4939827-T | 0.52 | 8.00E-28 | 1.2 | [1.16-1.24] |
| 19011631 | Colorectal cancer | RHPN2 | rs10411210-C | 0.9 | 5.00E-09 | 1.15 | [1.10-1.20] |
| 19011631 | Colorectal cancer | Intergenic | rs961253-A | 0.36 | 2.00E-10 | 1.12 | [1.08-1.16] |
| 18372905 | Colorectal cancer | EIF3H | rs16892766-A | 0.07 | 3.00E-18 | 1.27 | [1.20-1.34] |
| 18372905 | Colorectal cancer | Intergenic | rs6983267-? | 0.48 | 7.00E-11 | 1.24 | [1.17-1.33] |
| 19198612 | Coronary artery disease | MRAS | rs9818870-T | 0.15 | 7.00E-13 | 1.15 | [1.11-1.19] |
| 17634449 | Coronary disease | CXCL12 | rs501120-T | 0.87 | 9.00E-08 | 1.33 | [1.20-1.48] |
| 17634449 | Coronary disease | PSRC1 | rs599839-A | 0.77 | 4.00E-09 | 1.29 | [1.18-1.40] |
| 17634449 | Coronary disease | MTHFD1L | rs6922269-A | 0.25 | 3.00E-08 | 1.23 | [1.15-1.33] |
| 17634449 | Coronary disease | Intergenic | rs1333049-C | 0.47 | 3.00E-19 | 1.36 | [1.27-1.46] |
| 18587394 | Crohn's disease | Intergenic | rs17582416-G | 0.35 | 2.00E-09 | 1.16 | [NR] |
| 17435756 | Crohn's disease | Intergenic | rs224136-? | 0.81 | 1.00E-10 | 1.67 | [NR] |
| 18587394 | Crohn's disease | ZNF365 | rs10995271-C | 0.39 | 4.00E-20 | 1.25 | [NR] |
| 18587394 | Crohn's disease | NKX2-3 | rs11190140-T | 0.48 | 3.00E-16 | 1.2 | [NR] |
| 18587394 | Crohn's disease | C11orf30 | rs7927894-T | 0.39 | 1.00E-09 | 1.16 | [NR] |
| 18587394 | Crohn's disease | LRRK2, MUC19 | rs11175593-T | 0.02 | 3.00E-10 | 1.54 | [NR] |
| 18587394 | Crohn's disease | Unknown | rs3764147-G | 0.22 | 2.00E-13 | 1.25 | [NR] |
| 18587394 | Crohn's disease | NOD2 | rs2066847-C | 0.02 | 3.00E-24 | 3.99 | [NR] |
| 18587394 | Crohn's disease | ORMDL3 | rs2872507-A | 0.47 | 5.00E-09 | 1.12 | [NR] |
| 18587394 | Crohn's disease | STAT3 | rs744166-A | 0.57 | 7.00E-12 | 1.18 | [NR] |
| 18587394 | Crohn's disease | PTPN2 | rs2542151-G | 0.15 | 5.00E-17 | 1.35 | [NR] |
| 18587394 | Crohn's disease | PTPN22 | rs2476601-G | 0.9 | 1.00E-08 | 1.31 | [NR] |
| 17554300 | Crohn's disease | IL23R | rs11805303-T | 0.32 | 6.00E-12 | 1.39 | [1.22-1.58] |
| 18587394 | Crohn's disease | IL23R | rs11465804-T | 0.93 | 7.00E-63 | 2.5 | [NR] |
| 18587394 | Crohn's disease | ITLN1 | rs2274910-C | 0.68 | 1.00E-09 | 1.14 | [NR] |
| 18587394 | Crohn's disease | Intergenic | rs9286879-G | 0.24 | 2.00E-09 | 1.19 | [NR] |
| 17554261 | Crohn's disease | Intergenic | rs10801047-? | 0.08 | 3.00E-08 | 1.47 | [1.22-1.76] |
| 18587394 | Crohn's disease | Intergenic | rs11584383-T | 0.7 | 1.00E-11 | 1.18 | [NR] |
| 18587394 | Crohn's disease | Intergenic | rs1736135-T | 0.57 | 7.00E-09 | 1.18 | [NR] |
| 18587394 | Crohn's disease | ICOSLG | rs762421-G | 0.39 | 1.00E-09 | 1.13 | [NR] |
| 18587394 | Crohn's disease | ATG16L1 | rs3828309-G | 0.53 | 2.00E-32 | 1.25 | [NR] |
| 18587394 | Crohn's disease | MST1 | rs3197999-A | 0.27 | 1.00E-12 | 1.2 | [NR] |
| 17554261 | Crohn's disease | Intergenic | rs9292777-? | 0.4 | 3.00E-18 | 1.34 | [1.20-1.50] |
| 18587394 | Crohn's disease | PTGER4 | rs4613763-C | 0.13 | 7.00E-27 | 1.32 | [NR] |
| 18587394 | Crohn's disease | Intergenic | rs2188962-T | 0.43 | 2.00E-18 | 1.25 | [NR] |
| 18587394 | Crohn's disease | IRGM | rs11747270-G | 0.09 | 3.00E-16 | 1.33 | [NR] |
| 18587394 | Crohn's disease | IL12B | rs10045431-C | 0.71 | 4.00E-13 | 1.11 | [NR] |
| 18587394 | Crohn's disease | CDKAL1 | rs6908425-C | 0.78 | 9.00E-10 | 1.21 | [NR] |
| 18587394 | Crohn's disease | Unknown | rs7746082-C | 0.29 | 2.00E-10 | 1.17 | [NR] |
| 18587394 | Crohn's disease | CCR6 | rs2301436-T | 0.46 | 1.00E-12 | 1.21 | [NR] |
| 18587394 | Crohn's disease | Intergenic | rs1456893-A | 0.68 | 5.00E-09 | 1.2 | [NR] |
| 18587394 | Crohn's disease | Intergenic | rs1551398-A | 0.62 | 5.00E-09 | 1.08 | [NR] |
| 18587394 | Crohn's disease | JAK2 | rs10758669-C | 0.35 | 3.00E-09 | 1.12 | [NR] |
| 18587394 | Crohn's disease | TNFSF15 | rs4263839-G | 0.68 | 3.00E-10 | 1.22 | [NR] |
| 17952075 | Freckles | TYR | rs1042602-C | 0.67 | 2.00E-11 | 1.32 | [1.17-1.49] |
| 17952075 | Freckles | MC1R | rs1805007-T | 0.05 | 1.00E-96 | 4.37 | [3.56-5.37] |
| 17952075 | Freckles | SEC5L1,IRF4 | rs1540771-A | 0.42 | 4.00E-18 | 1.4 | [1.26-1.57] |
| 19896111 | Hair morphology | TCHH | rs11803731-A | 0.82 | 3.00E-31 | 6.11 | [NR] % variance |
| 18391951 | Height | HMGA2 | rs8756-C | 0.52 | 2.00E-16 | 6.6 | [5.03-8.17] % SD taller |
| 18391952 | Height | SOCS2 | rs11107116-G | 0.77 | 6.00E-10 | 0.04 | [0.01-0.07] SD shorter - among males |
| 18391952 | Height | DLEU7 | rs3116602-G | 0.21 | 7.00E-09 | 0.04 | [0.00-0.07] SD shorter - among males |
| 18391951 | Height | TRIP11, FBLN5, ATXN3, CPSF2 | rs7153027-A | 0.52 | 1.00E-10 | 5.7 | [3.94-7.46] % SD taller |
| 18391950 | Height | SH3GL3,ADAMTSL3 | rs2562784-G | 0.17 | 6.00E-08 | 0.34 | [0.21-0.48] cm taller |
| 18391951 | Height | ADAMTS17 | rs4533267-A | 0.28 | 3.00E-08 | 5.6 | [3.64-7.56] % SD taller % SD taller |
| 18391951 | Height | CRLF3, ATAD5, CENTA2, RNF135 | rs3760318-C | 0.63 | 2.00E-09 | 6 | [4.04-7.96] % SD taller % SD taller |
| 18391951 | Height | NOG, DGKE, TRIM25, COIL, RISK | rs4794665-A | 0.48 | 1.00E-07 | 3.6 | [2.23-4.97] % SD taller |
| 18391951 | Height | BCAS3, NACA2, TBX2, TBX4 | rs757608-T | 0.35 | 6.00E-08 | 4.4 | [2.83-5.97] % SD taller |
| 18391951 | Height | CABLES1, RBBP8, C18orf45 | rs4800148-A | 0.79 | 4.00E-09 | 6.4 | [4.24-8.56] % SD taller |
| 18391952 | Height | SPAG17 | rs12735613-A | 0.24 | 4.00E-11 | 0.08 | [0.05-0.11] SD shorter - among males |
| 18391952 | Height | SCMH1 | rs6686842-C | 0.56 | 2.00E-08 | 0.05 | [0.02-0.08] SD shorter - among males |
| 19343178 | Height | CATSPER4 | rs11809207-? | 0.23 | 6.00E-08 | 0.07 | [0.04-0.10] SD increase |
| 18391951 | Height | DNM3 | rs678962-G | 0.22 | 3.00E-08 | 5.4 | [3.44-7.36] % SD taller |
| 18391951 | Height | C1orf19,GLT25D2 | rs2274432-T | 0.37 | 8.00E-09 | 5.3 | [3.54-7.06] % SD taller |
| 18391952 | Height | ZNF678 | rs1390401-A | 0.82 | 5.00E-09 | 0.04 | [0.01-0.08] SD taller - among males |
| 18391951 | Height | BMP2 | rs967417-C | 0.53 | 2.00E-08 | 4.3 | [2.73-5.87] % SD taller |
| 18193045 | Height | BFZB | rs6060369-C | 0.44 | 2.00E-16 | 0.44 | [NR] cm taller |
| 18391951 | Height | EFEMP1, PNPT1 | rs3791679-T | 0.81 | 6.00E-11 | 5.8 | [4.04-7.56] % SD taller |
| 18391952 | Height | IHH | rs6724465-A | 0.1 | 2.00E-08 | 0.06 | [0.02-0.10] SD shorter - among males |
| 19570815 | Height | DIS3L2, ALPP, NPPC | rs6717918-T | 0.78 | 3.00E-09 | 0.44 | [0.20-0.68] cm increase |
| 18391952 | Height | ANAPC13,CEP63 | rs10935120-A | 0.33 | 7.00E-08 | 0.06 | [0.03-0.09] SD shorter - among males |
| 18391951 | Height | ZBTB38 | rs6763931-A | 0.45 | 1.00E-27 | 7.4 | [6.03-8.77] % SD taller |
| 18391951 | Height | LCORL, NCAPG | rs6830062-T | 0.89 | 1.00E-10 | 6.3 | [4.34-8.26] % SD taller |
| 19343178 | Height | PRKG2 | rs710841-? | 0.12 | 2.00E-08 | 0.07 | [0.04-0.10] SD increase |
| 18391951 | Height | HHIP | rs1812175-C | 0.86 | 1.00E-11 | 8.3 | [5.95-10.65] % SD taller |
| 18391951 | Height | ANKS1A, TCP11, ZNF76, DEF6,SCUBE3 | rs4713858-G | 0.86 | 4.00E-08 | 6.8 | [4.45-9.15] % SD taller |
| 18391951 | Height | HMGA1, LBH | rs1776897-C | 0.07 | 1.00E-08 | 8.8 | [5.66-11.94] % SD taller |
| 18391952 | Height | C6orf106 | rs2814993-A | 0.15 | 4.00E-12 | 0.09 | [0.05-0.13] SD taller - among males |
| 18391951 | Height | HLA class III | rs185819-T | 0.52 | 3.00E-08 | 5.2 | [3.44-6.96] % SD taller |
| 19343178 | Height | HLA-B | rs13437082-? | 0.13 | 5.00E-08 | 0.07 | [0.04-0.10] SD decrease |
| 18391951 | Height | Histone class 1,Butyrophilin genes | rs10946808-A | 0.7 | 6.00E-10 | 5.6 | [3.84-7.36] % SD taller |
| 18391951 | Height | BMP6 | rs12198986-A | 0.5 | 2.00E-11 | 6.8 | [4.84-8.76] % SD taller |
| 18391950 | Height | LIN28B | rs314277-A | 0.13 | 1.00E-08 | 0.41 | [0.26-0.59] cm taller |
| 18391952 | Height | LOC387103 | rs4549631-C | 0.5 | 5.00E-13 | 0.06 | [0.03-0.08] SD taller - among males |
| 18391951 | Height | GPR126 | rs3748069-A | 0.74 | 5.00E-14 | 6.5 | [5.44-9.36] % SD taller |
| 18952825 | Height | JAZF1 | rs1635852-A | NR | 9.00E-10 | 0.25 | [0.17-0.33] cm taller |
| 18391951 | Height | GNA12 | rs798544-G | 0.72 | 7.00E-15 | 5.9 | [6.03-8.77] % SD taller |
| 18391951 | Height | CDK6, PEX1, GATAD1, ERVWE1 | rs2282978-C | 0.29 | 1.00E-08 | 5.8 | [3.84-7.76] % SD taller |
| 18391951 | Height | PLAG1, MOS, CHCHD7,RDHE2, RPS20,LYN, TGS1, PENK | rs10958476-C | 0.23 | 7.00E-08 | 5.4 | [3.44-7.36] % SD taller |
| 18391951 | Height | PXMP3, ZFHX4 | rs7846385-C | 0.27 | 5.00E-08 | 5 | [3.24-6.76] % SD taller |
| 18391952 | Height | PTCH1 | rs10512248-G | 0.31 | 4.00E-11 | 0.05 | [0.02-0.07] SD taller - among males |
| 18391951 | Height | ZNF462 | rs4743034-A | 0.23 | 2.00E-08 | 5.3 | [3.54-7.06] % SD taller |
| 17068223 | Inflammatory bowel disease | IL23R | rs7517847-C | 0.56 | 4.00E-13 | 1.61 | [1.35-1.92] |
| 18758464 | Inflammatory bowel disease | NOD2 | rs5743289-T | 0.17 | 4.00E-10 | 1.46 | [1.29-1.64] |
| 18758464 | Inflammatory bowel disease | IL23R | rs11209026-? | 0.94 | 7.00E-11 | 2.56 | [1.92-3.45] |
| 18758464 | Inflammatory bowel disease | TNFRSF6B | rs2315008-G | 0.69 | 9.00E-15 | 1.36 | [1.05-1.76] |
| 18758464 | Inflammatory bowel disease | PSMG1 | rs2836878-? | 0.72 | 4.00E-12 | 1.41 | [1.08-1.84] |
| 18758464 | Inflammatory bowel disease | TNFSF15 | rs6478109-? | 0.69 | 3.00E-08 | 1.36 | [1.22-1.52] |
| 19915574 | Inflammatory bowel disease (early onset) | ZMIZ1 | rs1250550-? | 0.68 | 6.00E-09 | 1.16 | [1.09-1.25] |
| 19915574 | Inflammatory bowel disease (early onset) | IL27, CCDC101, CLN3, EIF3C, NUPR1, SULT1A1, SULT1A2 | rs8049439-G | 0.37 | 2.00E-09 | 1.14 | [1.00-1.30] |
| 19915574 | Inflammatory bowel disease (early onset) | Intergenic | rs10500264-? | 0.18 | 4.00E-10 | 1.21 | [1.11-1.31] |
| 19915574 | Inflammatory bowel disease (early onset) | HORMAD2, MTMR3, LIF | rs2412973-? | 0.46 | 2.00E-09 | 1.15 | [1.01-1.31] |
| 18991354 | Ischemic stroke | NR | rs2200733-T | 0.11 | 2.00E-10 | 1.26 | [1.17-1.35] |
| 19561606 | Kidney stones | CLDN14 | rs219780-C | 0.79 | 4.00E-12 | 1.25 | [1.17-1.33] |
| 19654303 | Lung cancer | CHRNA3 | rs8034191-? | NR | 3.00E-26 | 1.29 | [1.23-1.35] |
| 18978787 | Lung cancer | CLPTM1L | rs401681-G | NR | 8.00E-09 | 1.15 | [1.09-1.19] |
| 18978787 | Lung cancer | BAT3,MSH5 | rs3117582-C | NR | 5.00E-10 | 1.24 | [1.16-1.33] |
| 18849991 | Male-pattern baldness | PAX1 | rs1160312-A | 0.43 | 1.00E-14 | 1.6 | [1.42-1.80] (males) |
| 18849991 | Male-pattern baldness | AR | rs6625163-A | NR | 5.00E-11 | 3.3 | [2.31-4.71] |
| 19578364 | Melanoma | TYR | rs1393350-A | 0.27 | 2.00E-14 | 1.29 | [1.21-1.38] |
| 19578364 | Melanoma | MC1R | rs4785763-A | 0.32 | 6.00E-22 | 1.36 | [1.28-1.45] |
| 19578364 | Melanoma | MC1R | rs258322-A | 0.09 | 3.00E-27 | 1.67 | [1.52-1.83] |
| 18488026 | Melanoma | CDC91L1 | rs910873-T | 0.09 | 1.00E-15 | 1.75 | [1.53-2.01] |
| 19578364 | Melanoma | Intergenic | rs2284063-? | 0.37 | 2.00E-09 | 1.2 | [1.14-1.28] |
| 19525953 | Multiple sclerosis | IL2RA | rs2104286-T | 0.76 | 9.00E-08 | 1.15 | [1.04-1.27] |
| 19525953 | Multiple sclerosis | CD6 | rs17824933-G | 0.25 | 4.00E-09 | 1.18 | [1.07-1.30] |
| 19525953 | Multiple sclerosis | TNFRSF1A | rs1800693-C | 0.45 | 2.00E-11 | 1.2 | [1.10-1.31] |
| 19525955 | Multiple sclerosis | METTL1, CYP27B1 | rs703842-A | 0.67 | 5.00E-11 | 1.23 | [NR] |
| 19525953 | Multiple sclerosis | IRF8 | rs17445836-G | 0.81 | 4.00E-09 | 1.25 | [1.12-1.39] |
| 20159113 | Multiple sclerosis | STAT3 | rs744166-G | 0.41 | 3.00E-10 | 1.15 | [1.10-1.20] |
| 19525953 | Multiple sclerosis | CD58 | rs2300747-A | 0.88 | 3.00E-10 | 1.3 | [1.14-1.47] |
| 18997785 | Multiple sclerosis | KIF1B | rs10492972-C | 0.27 | 3.00E-10 | 1.34 | [1.23-1.48] |
| 19525955 | Multiple sclerosis | CD40 | rs6074022-G | 0.25 | 1.00E-07 | 1.2 | [NR] |
| 19525953 | Multiple sclerosis | CXCR4 | rs882300-C | 0.61 | 1.00E-07 | 1.19 | [1.09-1.30] |
| 19525953 | Multiple sclerosis | HLA-DRB1 | rs3135388-A | 0.22 | 4.00E-225 | 2.75 | [2.46-3.07] |
| 19525953 | Multiple sclerosis | HLA-B | rs2523393-A | 0.59 | 1.00E-17 | 1.28 | [1.18-1.39] |
| 19198609 | Myocardial infarction (early onset) | CXCL12 | rs1746048-C | 0.84 | 7.00E-09 | 1.17 | [1.11-1.24] |
| 19198609 | Myocardial infarction (early onset) | LDLR | rs1122608-G | 0.75 | 2.00E-09 | 1.15 | [1.10-1.20] |
| 19198609 | Myocardial infarction (early onset) | CELSR2, PSRC1, SORT1 | rs646776-T | 0.81 | 8.00E-12 | 1.19 | [1.13-1.26] |
| 19198609 | Myocardial infarction (early onset) | PCSK9 | rs11206510-T | 0.81 | 1.00E-08 | 1.15 | [1.10-1.21] |
| 19198609 | Myocardial infarction (early onset) | MIA3 | rs17465637-C | 0.72 | 1.00E-09 | 1.14 | [1.10-1.19] |
| 19198609 | Myocardial infarction (early onset) | SLC5A3, MRPS6, KCNE2 | rs9982601-T | 0.13 | 6.00E-11 | 1.2 | [1.14-1.27] |
| 19198609 | Myocardial infarction (early onset) | WDR12 | rs6725887-C | 0.14 | 1.00E-08 | 1.17 | [1.11-1.23] |
| 19198609 | Myocardial infarction (early onset) | PHACTR1 | rs12526453-C | 0.65 | 1.00E-09 | 1.12 | [1.08-1.17] |
| 19198609 | Myocardial infarction (early onset) | CDKN2A, CDKN2B | rs4977574-G | 0.56 | 3.00E-44 | 1.29 | [1.25-1.34] |
| 20112360 | Osteoarthritis | 7q22.3 | rs3815148-C | 0.23 | 8.00E-08 | 1.14 | [1.09-1.19] |
| 19648919 | Ovarian cancer | BNC2, LOC648570, CNTLN | rs3814113-T | 0.68 | 5.00E-19 | 1.22 | [1.16-1.27] |
| 20101243 | Pancreatic cancer | KLF5, KLF12 | rs9543325-C | 0.37 | 3.00E-11 | 1.26 | [1.18-1.35] |
| 20101243 | Pancreatic cancer | NR5A2 | rs3790844-T | 0.76 | 2.00E-10 | 1.3 | [1.19-1.41] |
| 19648918 | Pancreatic cancer | ABO | rs505922-C | 0.35 | 5.00E-08 | 1.2 | [1.12-1.28] |
| 19915575 | Parkinson's disease | CYP17A1, C10orf32, CNNM2, SFXN2 | rs17115100-G | 0.91 | 7.00E-08 | 1.25 | [NR] |
| 19915575 | Parkinson's disease | NSF | rs199533-C | 0.83 | 1.00E-14 | 1.28 | [NR] |
| 19915575 | Parkinson's disease | MAPT, C17orf69, KIAA1267, LOC644246, IMP5 | rs393152-A | 0.82 | 2.00E-16 | 1.3 | [NR] |
| 19915575 | Parkinson's disease | PARK16, NUCKS1 | rs823128-A | 0.97 | 7.00E-08 | 1.52 | [NR] |
| 19915575 | Parkinson's disease | SNCA | rs2736990-C | 0.51 | 2.00E-16 | 1.23 | [NR] |
| 19915575 | Parkinson's disease | MMRN1 | rs6532197-G | 0.09 | 1.00E-07 | 1.32 | [NR] |
| 18264097 | Prostate cancer | KLK3 | rs2735839-G | 0.85 | 2.00E-18 | 1.2 | [1.10-1.33] |
| 18264098 | Prostate cancer | EHBP1 | rs721048-A | 0.19 | 8.00E-09 | 1.15 | [1.10-1.21] |
| 18264097 | Prostate cancer | SLC22A3 | rs2660753-T | 0.11 | 3.00E-08 | 1.18 | [1.06-1.31] |
| 18264097 | Prostate cancer | SLC22A3 | rs9364554-T | 0.29 | 6.00E-10 | 1.17 | [1.08-1.26] |
| 18264097 | Prostate cancer | LMTK2 | rs6465657-C | 0.46 | 1.00E-09 | 1.12 | [1.05-1.20] |
| 18264097 | Prostate cancer | Intergenic | rs1016343-T | 0.18 | 1.00E-07 | 1.37 | [NR] |
| 18264098 | Prostate cancer | NUDT10, NUDT11, LOC340602, GSPT2, MAGED1 | rs5945572-A | 0.35 | 4.00E-13 | 1.23 | [1.16-1.30] |
| 18264096 | Prostate cancer | MSMB | rs10993994-T | 0.4 | 7.00E-13 | 1.16 | [1.04-1.29] |
| 19767753 | Prostate cancer | IGF2, IGF2AS, INS, TH | rs7127900-A | 0.2 | 3.00E-33 | 1.22 | [1.17-1.27] |
| 19767754 | Prostate cancer | Intergenic | rs11228565-A | 0.2 | 7.00E-12 | 1.23 | [1.16-1.31] |
| 18264096 | Prostate cancer | HNF1B | rs4430796-A | 0.54 | 1.00E-09 | 1.18 | [1.04-1.32] |
| 17603485 | Prostate cancer | Intergenic | rs1859962-G | 0.46 | 3.00E-10 | 1.2 | [1.14-1.27] |
| 19767754 | Prostate cancer | Intergenic | rs8102476-C | 0.54 | 2.00E-11 | 1.12 | [1.08-1.15] |
| 19767753 | Prostate cancer | NR | rs5759167-T | 0.53 | 6.00E-29 | 1.16 | [1.14-1.20] |
| 19767753 | Prostate cancer | THADA | rs1465618-A | 0.23 | 2.00E-08 | 1.08 | [1.03-1.12] |
| 19767753 | Prostate cancer | ITGA6 | rs12621278-A | 0.94 | 9.00E-23 | 1.33 | [1.25-1.43] |
| 19767754 | Prostate cancer | Intergenic | rs10934853-A | 0.28 | 3.00E-10 | 1.12 | [1.08-1.16] |
| 19767753 | Prostate cancer | PDLIM5 | rs17021918-C | 0.65 | 4.00E-15 | 1.11 | [1.08-1.15] |
| 19767753 | Prostate cancer | TET2 | rs7679673-A | 0.55 | 3.00E-14 | 1.1 | [1.06-1.14] |
| 19767753 | Prostate cancer | NKX3.1 | rs1512268-A | 0.45 | 3.00E-30 | 1.18 | [1.14-1.22] |
| 18264096 | Prostate cancer | Intergenic | rs6983267-G | 0.53 | 7.00E-12 | 1.28 | [1.15-1.45] |
| 18264096 | Prostate cancer | Intergenic | rs4242382-A | 0.12 | 3.00E-19 | 1.66 | [1.47-1.87] |
| 19767754 | Prostate cancer | Intergenic | rs445114-T | 0.64 | 5.00E-10 | 1.14 | [1.10-1.19] |
| 19767754 | Prostate cancer | Intergenic | rs1447295-A | 0.11 | 2.00E-19 | 1.58 | [1.43-1.74] |
| 19767754 | Prostate cancer | Intergenic | rs16901979-A | 0.04 | 3.00E-14 | 1.8 | [1.55-2.09] |
| 19169254 | Psoriasis | IL23A, STAT2 | rs2066808-A | 0.93 | 1.00E-09 | 1.34 | [NR] |
| 19169254 | Psoriasis | IL23R | rs2201841-G | 0.3 | 3.00E-08 | 1.13 | [NR] |
| 19169254 | Psoriasis | IL13 | rs20541-G | 0.79 | 5.00E-15 | 1.27 | [NR] |
| 19169254 | Psoriasis | TNIP1 | rs17728338-A | 0.054 | 1.00E-20 | 1.59 | [NR] |
| 19169254 | Psoriasis | IL12B | rs2082412-G | 0.8 | 2.00E-28 | 1.44 | [NR] |
| 19169254 | Psoriasis | HLA-C | rs12191877-T | 0.15 | 1.00E-100 | 2.64 | [NR] |
| 19169254 | Psoriasis | TNFAIP3 | rs610604-G | 0.32 | 9.00E-12 | 1.19 | [NR] |
| 17952075 | Red vs non-red hair color | MC1R | rs1805007-T | NR | 2.00E-142 | 12.47 | [9.37-16.60] |
| 18794853 | Rheumatoid arthritis | KIF5A,PIP4K2C | rs1678542-C | 0.37 | 9.00E-08 | 1.12 | [NR] |
| 18794853 | Rheumatoid arthritis | PTPN22 | rs6679677-? | 0.1 | 6.00E-42 | 1.79 | [1.65-1.94] |
| 18794853 | Rheumatoid arthritis | MMEL1,TNFRSF14 | rs3890745-T | 0.67 | 1.00E-07 | 1.12 | [NR] |
| 18794853 | Rheumatoid arthritis | CD40 | rs4810485-G | 0.75 | 8.00E-09 | 1.15 | [NR] |
| 19503088 | Rheumatoid arthritis | REL | rs13017599-A | 0.34 | 2.00E-12 | 1.21 | [1.15-1.28] |
| 19503088 | Rheumatoid arthritis | CTLA4 | rs231735-T | 0.51 | 6.00E-09 | 1.17 | [1.11-1.23] |
| 17982456 | Rheumatoid arthritis | TNFAIP3, OLIG3 | rs10499194-C | 0.71 | 1.00E-09 | 1.33 | [1.15-1.52] |
| 18794853 | Rheumatoid arthritis | OLIG3, TNFIP3 | rs6920220-? | 0.22 | 2.00E-09 | 1.24 | [1.16-1.32] |
| 19503088 | Rheumatoid arthritis | BLK | rs2736340-A | 0.24 | 6.00E-09 | 1.19 | [1.13-1.27] |
| 18794853 | Rheumatoid arthritis | CCL21 | rs2812378-G | 0.34 | 3.00E-08 | 1.12 | [NR] |
| 17804836 | Rheumatoid arthritis | TRAF1-C5 | rs3761847-G | 0.41 | 4.00E-14 | 1.32 | [1.23-1.42] |
| 19571808 | Schizophrenia | NRGN | rs12807809-T | 0.83 | 2.00E-09 | 1.15 | [NR] |
| 19571808 | Schizophrenia | TCF4 | rs9960767-C | 0.06 | 4.00E-09 | 1.23 | [NR] |
| 19571811 | Schizophrenia | FXR1 | rs6782299-T | 0.73 | 1.00E-07 | 1.1 | (empty) |
| 19571808 | Schizophrenia | MHC, NOTCH4 | rs3131296-G | 0.87 | 2.00E-10 | 1.19 | [NR] |
| 19571809 | Schizophrenia | HLA-DQA1 | rs9272219-G | 0.72 | 7.00E-08 | 1.14 | [NR] |
| 19571808 | Schizophrenia | MHC, PRSS16 | rs6932590-T | 0.78 | 1.00E-12 | 1.16 | [NR] |
| 19571811 | Schizophrenia | MHC | rs13194053-T | 0.86 | 1.00E-08 | 1.22 | (empty) |
| 19369658 | Stroke | NINJ2 | rs12425791-A | 0.19 | 1.00E-09 | 1.29 | [1.19-1.41] |
| 18204446 | Systemic lupus erythematosus | KIAA1542 | rs4963128-? | 0.34 | 3.00E-10 | 1.28 | [1.18-1.37] |
| 18204446 | Systemic lupus erythematosus | ITGAM | rs9888739-T | 0.13 | 2.00E-23 | 1.62 | [1.47-1.78] |
| 18204446 | Systemic lupus erythematosus | Intergenic | rs10798269-? | 0.64 | 1.00E-07 | 1.22 | [1.14-1.32] |
| 19165918 | Systemic lupus erythematosus | STAT4 | rs3821236-? | 0.19 | 8.00E-11 | 1.49 | [NR] |
| 18204446 | Systemic lupus erythematosus | PXK | rs6445975-C | 0.28 | 7.00E-09 | 1.25 | [1.16-1.35] |
| 18204447 | Systemic lupus erythematosus | BANK1 | rs10516487-G | 0.77 | 4.00E-10 | 1.38 | [1.25-1.53] |
| 18204446 | Systemic lupus erythematosus | HLA region | rs3131379-A | 0.1 | 2.00E-52 | 2.36 | [2.11-2.64] |
| 18204446 | Systemic lupus erythematosus | HLA region | rs3131379-A | 0.1 | 2.00E-52 | 2.36 | [2.11-2.64] |
| 18204098 | Systemic lupus erythematosus | C8orf13, BLK | rs13277113-A | 0.23 | 1.00E-10 | 1.39 | [1.28-1.51] |
| 20383147 | Systemic sclerosis | CD247 | rs2056626-? | 0.59 | 3.00E-09 | 1.16 | [1.11-1.23] |
| 20383147 | Systemic sclerosis | STAT4 | rs3821236-A | 0.2 | 3.00E-09 | 1.3 | [1.19-1.41] |
| 20383147 | Systemic sclerosis | HLADQB1 | rs6457617-? | 0.53 | 4.00E-17 | 1.37 | [1.28-1.47] |
| 20383147 | Systemic sclerosis | TNPO, IRF5 | rs10488631-C | NR | 2.00E-13 | 1.5 | [1.35-1.67] |
| 19340012 | Tanning | GRM5 | rs10831496-G | NR | 5.00E-09 | 0.14 | [0.10-0.18] decrease in tanning ability score |
| 19340012 | Tanning | TYR | rs1393350-A | NR | 2.00E-13 | 0.19 | [0.13-0.25] increase in tanning ability score |
| 19340012 | Tanning | Intergenic | rs17094273-A | NR | 9.00E-08 | 0.2 | [0.12-0.28] increase (ERROR - RISK WRONG - thus no flip) in tanning ability score |
| 19340012 | Tanning | MC1R | rs11648785-T | NR | 3.00E-09 | 0.14 | [0.10-0.18] decrease in tanning ability score |
| 19340012 | Tanning | MC1R | rs154659-C | NR | 7.00E-08 | 0.14 | [0.08-0.20] increase in tanning ability score |
| 19340012 | Tanning | MATP | rs35391-T | NR | 3.00E-10 | 0.44 | [0.30-0.58] decrease in tanning ability score |
| 19340012 | Tanning | EXOC2 | rs12210050-T | NR | 5.00E-14 | 0.22 | [0.16-0.28] increase in tanning ability score |
| 19483681 | Testicular germ cell tumor | KITLG | rs995030-G | 0.8 | 1.00E-31 | 2.55 | [2.05-3.19] |
| 19483681 | Testicular germ cell tumor | SPRY4 | rs4624820-A | 0.54 | 3.00E-13 | 1.37 | [1.19-1.58] |
| 19483681 | Testicular germ cell tumor | BAK1 | rs210138-G | 0.2 | 1.00E-13 | 1.5 | [1.28-1.75] |
| 19198613 | Thyroid cancer | NKX2-1 | rs944289-T | 0.57 | 2.00E-09 | 1.37 | [1.24-1.52] |
| 19198613 | Thyroid cancer | FOXE1 | rs965513-A | 0.34 | 2.00E-27 | 1.75 | [1.59-1.94] |
| 18978792 | Type 1 diabetes | PRKCQ | rs947474-G | 0.19 | 4.00E-09 | 1.1 | [1.03-1.18] |
| 19430480 | Type 1 diabetes | C10orf59 | rs10509540-? | 0.71 | 1.00E-28 | 1.33 | [1.25-1.43] |
| 17632545 | Type 1 diabetes | INS | rs1004446-C | 0.65 | 4.00E-09 | 1.61 | [1.37-1.89] |
| 19430480 | Type 1 diabetes | CD69 | rs4763879-A | 0.37 | 2.00E-11 | 1.09 | [1.02-1.16] |
| 17554260 | Type 1 diabetes | ERBB3 | rs2292239-A | 0.34 | 2.00E-20 | 1.28 | [1.21-1.35] |
| 17554260 | Type 1 diabetes | C12orf30 | rs17696736-G | 0.42 | 2.00E-16 | 1.22 | [1.15-1.28] |
| 19430480 | Type 1 diabetes | Intergenic | rs1465788-? | 0.71 | 2.00E-12 | 1.16 | [1.10-1.25] |
| 19430480 | Type 1 diabetes | Intergenic | rs4900384-G | 0.29 | 4.00E-09 | 1.09 | [1.02-1.16] |
| 18978792 | Type 1 diabetes | CTSH | rs3825932-T | 0.68 | 3.00E-15 | 1.16 | [1.10-1.22] |
| 19430480 | Type 1 diabetes | IL27 | rs4788084-G | 0.42 | 3.00E-13 | 1.09 | [1.02-1.16] |
| 17554260 | Type 1 diabetes | KIAA0350 | rs12708716-A | 0.68 | 3.00E-18 | 1.23 | [1.16-1.30] |
| 19430480 | Type 1 diabetes | Intergenic | rs7202877-G | 0.1 | 3.00E-15 | 1.28 | [1.17-1.41] |
| 19430480 | Type 1 diabetes | ORMDL3 | rs2290400-? | 0.5 | 6.00E-13 | 1.15 | [1.08-1.22] |
| 19430480 | Type 1 diabetes | Intergenic | rs7221109-? | 0.65 | 1.00E-09 | 1.05 | [0.99-1.12] |
| 17554260 | Type 1 diabetes | PTPN2 | rs2542151-C | 0.16 | 1.00E-14 | 1.3 | [1.22-1.40] |
| 17554260 | Type 1 diabetes | CD226 | rs763361-A | 0.47 | 1.00E-08 | 1.16 | [1.10-1.22] |
| 19430480 | Type 1 diabetes | Intergenic | rs425105-? | 0.84 | 3.00E-11 | 1.16 | [1.08-1.27] |
| 17554260 | Type 1 diabetes | PHTF1, PTPN22 | rs6679677-A | 0.1 | 8.00E-24 | 1.89 | [1.67-2.13] |
| 19430480 | Type 1 diabetes | IL10 | rs3024505-? | 0.83 | 2.00E-09 | 1.19 | [1.10-1.30] |
| 19430480 | Type 1 diabetes | Intergenic | rs2281808-? | 0.64 | 1.00E-11 | 1.11 | [1.05-1.19] |
| 18840781 | Type 1 diabetes | UBASH3A | rs9976767-C | NR | 2.00E-08 | 1.16 | [1.10-1.22] |
| 19430480 | Type 1 diabetes | Intergenic | rs5753037-T | 0.39 | 3.00E-16 | 1.1 | [1.04-1.17] |
| 18978792 | Type 1 diabetes | C1QTNF6 | rs229541-T | 0.43 | 2.00E-08 | 1.11 | [1.05-1.16] |
| 17554260 | Type 1 diabetes | IFIH1 | rs1990760-A | 0.6 | 2.00E-11 | 1.18 | [1.11-1.23] |
| 19430480 | Type 1 diabetes | Intergenic | rs10517086-A | 0.3 | 5.00E-10 | 1.09 | [1.02-1.17] |
| 17554300 | Type 1 diabetes | MHC | rs9272346-G | 0.61 | 5.00E-134 | 5.49 | [4.83-6.24] |
| 17632545 | Type 1 diabetes | HLA-DRB1 | rs2647044-A | 0.13 | 1.00E-16 | 8.3 | [6.97-9.89] |
| 18978792 | Type 1 diabetes | BACH2 | rs11755527-G | 0.47 | 5.00E-12 | 1.13 | [1.08-1.19] |
| 19430480 | Type 1 diabetes | C6orf173 | rs9388489-G | 0.45 | 4.00E-13 | 1.17 | [1.10-1.24] |
| 19430480 | Type 1 diabetes | Intergenic | rs7804356-? | 0.76 | 5.00E-09 | 1.14 | [1.06-1.22] |
| 19430480 | Type 1 diabetes | GLIS3 | rs7020673-? | 0.5 | 5.00E-12 | 1.14 | [1.08-1.20] |
| 19430480 | Type 1 diabetes | Intergenic | rs2664170-G | 0.32 | 8.00E-09 | 1.16 | [1.07-1.24] |
| 18372903 | Type 2 diabetes | CDC123,CAMK1D | rs12779790-G | 0.18 | 1.00E-10 | 1.11 | [1.07-1.14] |
| 18372903 | Type 2 diabetes | HHEX | rs5015480-C | NR | 7.00E-08 | 1.17 | [1.11-1.24] |
| 18372903 | Type 2 diabetes | TCF7L2 | rs7903146-T | NR | 3.00E-23 | 1.37 | [1.28-1.47] |
| 17463248 | Type 2 diabetes | Intergenic | rs9300039-C | 0.89 | 6.00E-08 | 1.48 | [1.28-1.71] |
| 17463246 | Type 2 diabetes | KCNJ11 | rs5219-T | 0.47 | 1.00E-07 | 1.15 | [1.09-1.21] |
| 17463249 | Type 2 diabetes | KCNJ11 | rs5215-C | NR | 5.00E-11 | 1.14 | [1.10-1.19] |
| 18372903 | Type 2 diabetes | TSPAN8,LGR5 | rs7961581-C | 0.27 | 1.00E-09 | 1.09 | [1.06-1.12] |
| 17463249 | Type 2 diabetes | FTO | rs8050136-A | 0.4 | 7.00E-14 | 1.23 | [1.18-1.32] |
| 18372903 | Type 2 diabetes | NOTCH2, ADAM30 | rs10923931-T | 0.11 | 4.00E-08 | 1.13 | [1.08-1.17] |
| 18372903 | Type 2 diabetes | THADA | rs7578597-T | 0.9 | 1.00E-09 | 1.15 | [1.10-1.20] |
| 18372903 | Type 2 diabetes | ADAMTS9 | rs4607103-C | 0.76 | 1.00E-08 | 1.09 | [1.06-1.12] |
| 18372903 | Type 2 diabetes | IGF2BP2 | rs4402960-T | NR | 8.00E-08 | 1.17 | [1.10-1.25] |
| 18372903 | Type 2 diabetes | CDKAL1 | rs6931514-G | NR | 1.00E-11 | 1.25 | [1.17-1.33] |
| 18372903 | Type 2 diabetes | JAZF1 | rs864745-T | 0.5 | 5.00E-14 | 1.1 | [1.07-1.13] |
| 17463246 | Type 2 diabetes | SLC30A8 | rs13266634-C | 0.65 | 5.00E-08 | 1.12 | [1.07-1.16] |
| 17463246 | Type 2 diabetes | CDKN2A,CDKN2B | rs10811661-T | 0.83 | 5.00E-08 | 1.2 | [1.12-1.28] |
| 19734900 | Type 2 diabetes and other traits | LOC64673, IRS1 | rs2943641-C | 0.63 | 9.00E-12 | 1.19 | [1.13-1.25] |
| 19734900 | Type 2 diabetes and other traits | WFS1, PPP2R2C | rs4689388-T | 0.57 | 1.00E-08 | 1.16 | [1.10-1.21] |
| 19734900 | Type 2 diabetes and other traits | CDKAL1 | rs4712523-G | 0.32 | 2.00E-12 | 1.2 | [1.14-1.26] |
| 20228799 | Ulcerative colitis | NKX2-3 | rs11190140-T | NR | 1.00E-08 | 1.2 | [NR] |
| 20228799 | Ulcerative colitis | IFNG,IL26 | rs1558744-A | NR | 4.00E-12 | 1.16 | [NR] |
| 19915572 | Ulcerative colitis | CDH1 | rs1728785-G | 0.76 | 3.00E-08 | 1.17 | [1.07-1.27] |
| 20228799 | Ulcerative colitis | GSDMB | rs8067378-A | NR | 1.00E-07 | 1.12 | [NR] |
| 19122664 | Ulcerative colitis | IL23R | rs11209026-? | 0.93 | 1.00E-08 | 1.79 | (empty) |
| 20228799 | Ulcerative colitis | IL23R | rs2201841-A | NR | 1.00E-13 | 1.27 | [NR] |
| 19122664 | Ulcerative colitis | RNF186 | rs3806308-? | 0.63 | 7.00E-09 | 1.28 | (empty) |
| 20228799 | Ulcerative colitis | RNF186,OTUD3,PLA2G2E | rs1317209-T | NR | 2.00E-10 | 1.17 | [NR] |
| 20228799 | Ulcerative colitis | RNF186,OTUD3,PLA2G2E | rs6426833-A | NR | 2.00E-21 | 1.3 | [NR] |
| 20228799 | Ulcerative colitis | FCGR2A,FCGR2C | rs10800309-A | NR | 3.00E-09 | 1.2 | [NR] |
| 20228799 | Ulcerative colitis | IL10,IL19 | rs3024505-T | NR | 1.00E-08 | 1.23 | [NR] |
| 19915572 | Ulcerative colitis | HNF4A | rs6017342-C | 0.52 | 9.00E-17 | 1.17 | [1.09-1.26] |
| 20228798 | Ulcerative colitis | IL17REL | rs5771069-G | 0.49 | 4.00E-08 | 1.17 | [1.11-1.25] |
| 20228799 | Ulcerative colitis | REL,CCDC139,PUS10 | rs13003464-G | NR | 7.00E-09 | 1.13 | [NR] |
| 20228799 | Ulcerative colitis | MST1 | rs3197999-T | NR | 4.00E-09 | 1.2 | [NR] |
| 20228799 | Ulcerative colitis | CEP72,TPPP | rs4957048-C | NR | 1.00E-09 | 1.3 | [NR] |
| 20228798 | Ulcerative colitis | HLA-DRA | rs9268923-C | 0.67 | 4.00E-15 | 1.45 | [1.33-1.59] |
| 20228798 | Ulcerative colitis | SMURF1,KPNA7 | rs7809799-G | 0.04 | 9.00E-11 | 1.56 | [1.36-1.78] |
| 20228799 | Ulcerative colitis | DLD,LAMB1 | rs4598195-A | NR | 8.00E-08 | 1.09 | [NR] |
| 20228799 | Ulcerative colitis | CARD9 | rs4077515-C | NR | 5.00E-08 | 1.14 | [NR] |
| 18794855 | Urinary bladder cancer | TP63 | rs710521-A | 0.73 | 1.00E-07 | 1.19 | [1.12-1.27] |
| 20348956 | Urinary bladder cancer | TACC3,TMEM129,SLBP,FGFR3 | rs798766-T | 0.19 | 1.00E-11 | 1.24 | [1.17-1.32] |
| 18794855 | Urinary bladder cancer | MYC, BC042052 | rs9642880-T | 0.45 | 9.00E-12 | 1.22 | [1.15-1.29] |
| 19278955 | Venous thromboembolism | ABO | rs505922-C | 0.35 | 4.00E-15 | 1.81 | [1.56-2.11] |
