# Supplementary material for: Efficient Replication of over 180 Genetic Associations with Self-Reported Medical Data
Source: PLoS One. 2011 Aug 17;6(8):e23473. doi: 10.1371/journal.pone.0023473 (PMC3157390; doi:10.1371/journal.pone.0023473)
Supplement: Table S8 — Data from GWAS catalog for successful associations without strictly matching phenotypes. PMID: PubMed ID. RAF = risk allele frequency. OR/Beta = odds ratio or beta (effect size). CI = confidence interval. (DOCX) [file pone.0023473.s010.docx]

**Table S8**

| **PMID** | **Disease** | **Region** | **SNP-Risk allele** | **RAF** | **P-value** | **OR/Beta** | **CI** |
| --- | --- | --- | --- | --- | --- | --- | --- |
| 20385819 | Age-related macular degeneration | C3 | rs2230199-C | 0.163 | 1.00E-09 | 1.74 | [1.47-2.06] |
| 20385819 | Age-related macular degeneration | C2 | rs429608-G | 0.012 | 2.00E-08 | 2.16 | [1.84-2.53] |
| 19419973 | Bilirubin levels | UGT1A1 | rs887829-T | 0.3 | 1.00E-69 | 0.57 | [0.50-0.63] SD decrease |
| 19801982 | Bone mineral density (hip) | MEF2C | rs1366594-C | 0.45 | 1.00E-13 | 0.09 | [0.07-0.11] sd decrease |
| 19801982 | Bone mineral density (hip) | SOX6 | rs7117858-G | 0.2 | 6.00E-10 | 0.09 | [0.06-0.12] sd increase |
| 19079262 | Bone mineral density (spine) | OSX | rs10876432-A | 0.73 | 1.00E-07 | 0.08 | [0.05-0.11] SD decrease |
| 19801982 | Bone mineral density (spine) | SPTBN1 | rs11898505-A | 0.34 | 2.00E-08 | 0.07 | [0.05-0.09] sd increase |
| 19801982 | Bone mineral density (spine) | MEPE | rs1471403-T | 0.34 | 8.00E-07 | 0.07 | [0.05-0.09] sd increase |
| 19060911 | Cholesterol, total | DOCK7 | rs10889353-C | 0.32 | 4.00E-12 | 0.08 | [NR] SD decrease |
| 19060911 | Cholesterol, total | LDLR | rs2228671-C | 0.88 | 9.00E-24 | 0.16 | [NR] SD increase |
| 19430483 | Diastolic blood pressure | CYP1A1 | rs1378942-C | 0.36 | 1.00E-23 | 0.43 | [0.35-0.51] mm Hg increase |
| 19430483 | Diastolic blood pressure | FGF5 | rs16998073-T | 0.21 | 1.00E-21 | 0.5 | [0.40-0.60] mm Hg increase |
| 19430483 | Diastolic blood pressure | ZNF652 | rs16948048-G | 0.39 | 5.00E-09 | 0.31 | [0.21-0.41] mm Hg increase |
| 19430479 | Diastolic blood pressure | ATP2B1 | rs2681472-A | 0.83 | 1.00E-09 | 0.5 | [0.34-0.66] mm Hg increase |
| 19430479 | Diastolic blood pressure | SH2B3 | rs3184504-T | 0.48 | 3.00E-14 | 0.48 | [0.36-0.60] mm Hg increase |
| 19430479 | Diastolic blood pressure | ULK4 | rs9815354-A | 0.17 | 3.00E-09 | 0.49 | [0.33-0.65] mm Hg increase |
| 17632509 | Gallstones | ABCG8 | rs11887534-C | 0.1 | 1.00E-14 | 2.2 | [1.80-2.60] |
| 19060906 | HDL cholesterol | LIPC | rs10468017-T | 0.3 | 8.00E-23 | 0.1 | [0.06-0.14] SD increase |
| 18193044 | HDL cholesterol | LIPC | rs1800588-T | 0.21 | 2.00E-32 | 0.14 | [0.12-0.16]% SD higher |
| 19060906 | LDL cholesterol | PCSK9 | rs11206510-C | 0.19 | 4.00E-08 | 0.09 | [0.05-0.13] SD decrease |
| 18193044 | LDL cholesterol | PCSK9 | rs11591147-T | 0.01 | 2.00E-44 | 0.47 | [0.41-0.53] % SD lower |
| 19060906 | LDL cholesterol | HNF1A | rs2650000-A | 0.36 | 2.00E-08 | 0.07 | [0.03-0.11] SD increase |
| 19060911 | LDL cholesterol | APOA1 | rs12272004-C | 0.93 | 5.00E-13 | 0.18 | [NR] SD decrease |
| 18193043 | LDL cholesterol | CILP2 | rs16996148-G | 0.89 | 3.00E-09 | 3.32 | [NR] mg/dl higher |
| 19060911 | LDL cholesterol | DNAH11 | rs12670798-C | 0.24 | 6.00E-09 | 0.09 | [NR] SD increase |
| 19060910 | LDL cholesterol | TOMM40 | rs157580-G | 0.29 | 5.00E-08 | 0.11 | [0.07-0.15] mmol/l decrease |
| 19060910 | LDL cholesterol | FADS1 | rs174546-T | 0.44 | 1.00E-07 | 0.1 | [0.06-0.13] mmol/l decrease |
| 19060906 | LDL cholesterol | NCAN | rs10401969-C | 0.06 | 2.00E-08 | 0.05 | [-0.03-0.13] SD decrease |
| 19060906 | LDL cholesterol | APOB | rs515135-T | 0.2 | 5.00E-29 | 0.16 | [0.12-0.20] SD decrease |
| 19060906 | LDL cholesterol | MAFB | rs6102059-T | 0.32 | 4.00E-09 | 0.06 | [0.03-0.10] SD decrease |
| 19060906 | LDL cholesterol | CELSR2 | rs12740374-T | 0.21 | 2.00E-42 | 0.23 | [0.19-0.27] SD decrease |
| 19060906 | LDL cholesterol | ABCG8 | rs6544713-T | 0.32 | 2.00E-20 | 0.15 | [0.11-0.19] SD increase |
| 18385739 | Nicotine dependence | CHRNA3 | rs1051730-T | 0.35 | 6.00E-20 | 0.1 | [0.08-0.12] increase in cigarettes per day |
| 18940312 | Plasma levels of liver enzymes | GGT1 | rs4820599-G | 0.31 | 4.00E-11 | 0.01 | [0.005-0.009] U/L increase |
| 18940312 | Plasma levels of liver enzymes | PNPLA3 | rs2281135-A | 0.18 | 8.00E-16 | 0.06 | [0.046-0.074] U/L increase |
| 19458352 | Primary biliary cirrhosis | IL12RB2 | rs3790567-A | 0.24 | 3.00E-11 | 1.51 | [1.33-1.70] |
| 19430479 | Systolic blood pressure | CYP17A1 | rs1004467-A | 0.9 | 1.00E-10 | 1.05 | [0.74-1.36] mm Hg increase |
| 19430483 | Systolic blood pressure | CYP17A1 | rs11191548-T | 0.91 | 7.00E-24 | 1.16 | [0.92-1.40] mm Hg increase |
| 19060906 | Triglycerides | GCKR | rs1260326-T | 0.45 | 2.00E-31 | 0.12 | [0.08-0.16] SD increase |
| 18193043 | Triglycerides | TRIB1 | rs17321515-A | 0.56 | 7.00E-13 | 6.42 | [NR] mg/dl higher |
